# Optimization of the Composite Formulation for Rotational Molding Based on Recycled Polyethylene and Antioxidants

**DOI:** 10.3390/polym18192332

**Published:** 2026-09-24

**Authors:** Vitaliy Tyukanko, Roman Tarunin, Alexandr Demyanenko, Vladislav Semenyuk, Antonina Dyuryagina, Yerik Merkibayev, Abdigali Bakibaev, Alisher Kussainov, Roman Krasilnikov, Dmitriy Alyoshin

**Affiliations:** 1 Department of Chemistry and Chemical Technology, M. Kozybayev North Kazakhstan University, Petropavlovsk 150000, Kazakhstan; vyutyukanko@ku.edu.kz (V.T.); demianenkoav@mail.ru (A.D.); evdimid@mail.ru (V.S.); adyuryagina@inbox.ru (A.D.); alisher2909@gmail.com (A.K.); krasilnikov.roman.01@mail.ru (R.K.); dvaleshin@ku.edu.kz (D.A.); 2 Department of Metallurgy and Mineral Processing, Kazakh National Research Technical University Named After K.I. Satpayev, Almaty 050013, Kazakhstan; y.merkibayev@satbayev.university; 3 Department of Organic Chemistry, Tomsk State University, Tomsk 634050, Russia; bakibaev@mail.ru

**Keywords:** rotomolding, polyethylene recycling, rPE, internal stresses in plastics, ultrasound in plastics, higher harmonics, antioxidants, stabilizers, probabilistic–deterministic planning, carbon black

## Abstract

In this work, the content of recycled polyethylene (rPE) and pigment (Cp), the antioxidant content (CAO) and ratio (RAO), and the peak internal air temperature (PIAT) of the rotational-molding cycle were optimized. Their combined influence on the mechanical, thermal, acoustic, and surface properties of the composites was studied by the probabilistic–deterministic planning method, which reduced the number of experiments from 3125 to 25. Internal stresses were monitored non-destructively through the third-harmonic amplitude (β) of the ultrasonic signal (mirror-shadow method), supported by mechanical testing, thermogravimetric analysis, infrared spectroscopy, and porosity characterization; weather resistance was assessed by accelerated ultraviolet (UV)-B aging for 333 and 666 h, treated as conditional 5- and 10-year service horizons for Northern Kazakhstan rather than as a literal calendar equivalence. The third-harmonic β was shown to be a sensitive correlational marker of internal stresses (R = 0.81 for the mean failure energy (MFE), Fisher-criterion validated), correlating with the elastic modulus, the impact strength, and the environmental stress cracking resistance (ESCR), and tracking the transition between incomplete (ICS), normal (NS), and thermo-oxidative degradation (TDS) sintering; the correlational nature of this evidence is discussed. For products without UV exposure, the optimal formulation (rPE = 25%, PIAT ≈ 200 °C, Cp = 0.1–0.2%, CAO = 0.25–0.5%, RAO = 0.5–1.0) gave a minimum β (0.076–0.09), MFE ≈ 2.6 J/mm, and 2.5–3% porosity; for sun-exposed products, rPE = 25%, Cp = 0.5%, CAO = 0.5–0.75%, and RAO = 1.0 are recommended. An rPE content of up to 50% did not significantly impair thermal stability or surface hydrophobicity, and a nomogram CAO = f(β,rPE) was developed for rapid formulation selection and quality control.

## 1. Introduction

Today, the efficient use and recycling of plastic waste remain among the most pressing global challenges. Since the beginning of the twentieth century, plastics have been an integral part of industrial production and everyday life, and the global volume of their production has grown continuously, rising from 395.3 million tonnes in 2018 to 459.3 million tonnes in 2024 [1]. According to OECD estimates, only about 9% of plastic waste is recycled worldwide [2,3]. In Kazakhstan, the utilization of polymer waste is being developed both through its incorporation into construction products [4] and through the improvement of production- and consumption-waste management practices [5]. According to analytical reviews, the share of polyethylene in the structure of plastic materials and waste may exceed 70% [3,6,7,8,9,10,11].

One of the promising methods for recycling plastic waste is rotational molding. Rotational molding is a polymer processing method based on the heating of a powdered material inside a closed rotating mold [12,13]. Carrying out the process without pronounced shear deformation ensures a low level of residual stresses and the formation of large hollow products with uniform wall thickness and high operational reliability [13]. Industry analytical reviews forecast steady growth of the recycled-polyethylene market and of the rotational molding sector, reflecting the expanding use of recyclates in the production of tank and engineering products [14,15]. About 95–97% of the materials used in rotational molding are various grades of polyethylene (PE) [13,16], owing to its low melting temperature, good processability at low pressure, and ability to form strong, seamless products [17]. In recent years, interest in the use of recycled polyethylene in rotational molding has been increasing, driven by economic and environmental factors [18,19]. Despite the growth of global plastics production and the accumulation of large volumes of waste, their recycling rate remains low [18,20], which stimulates the introduction of post-consumer recycled (PCR) polyethylene within the framework of the circular-economy concept [18,19]. Rotational molding has been shown to accommodate substantial volumes of recyclate, yielding large products with a recycled-PE content of 20–50% without a critical deterioration of the performance properties [15,18,21,22,23,24,25].

However, under the prolonged thermal exposure typical of rotational molding, PE is subject to thermo-oxidative degradation [13,26]. This necessitates the use of stabilized PE grades and imposes increased requirements on the thermal stability of the material even for virgin polyethylene, while for recycled polyethylene these factors are even more significant [13,27]. Thermo-oxidative degradation leads to the shortening of molecular chains, an increase in the melt flow rate [28,29], and the deterioration of mechanical characteristics, including a decrease in impact strength and a transition to brittle failure of the products [13,30,31]. Insufficient mechanical characteristics of the principal rotational molding products—large agricultural tanks for the storage of liquid mineral fertilizers and tank mixes containing toxic fungicides, herbicides, and insecticides—may lead to irreversible environmental damage through the ingress of toxic substances into the soil and groundwater upon product failure.

To suppress oxidative processes, antioxidants are introduced into industrial grades of rotational PE, usually as a combination of primary (phenolic) and secondary (phosphite) stabilizers with a pronounced synergistic effect [18,26,27,31]. A typical composition of PE compounds for rotational molding based on virgin polyethylene includes 0.05–10% phenolic antioxidant [30,32,33,34,35], 0.01–1% phosphite stabilizer [33,36,37], and 0.05–10% auxiliary stabilizing additives [30,31,34,38]; for recycled polyethylene, the development of even more flexible stabilizer packages is required, taking into account the degree of recyclate degradation, the presence of residual impurities, and the increased tendency toward thermo-oxidative degradation [18,24]. For products intended for long-term outdoor service, light stabilizers are additionally applied, and in industrial practice, combined stabilizer packages are used, often supplemented with carbon black acting as a pigment, which preserves the mechanical properties and tightness of the products [8,31,39,40].

Recycled polyethylene has a heterogeneous composition, the presence of impurities, an altered rheology, and, as a rule, a depleted stabilizer package [25,41,42], leading to reduced thermo-oxidative stability, an increase in the melt flow rate, and a deterioration of the impact strength of the products [25,43]. It has been shown that, without additional stabilization, even small fractions of recyclate may substantially impair the properties of rotational composites, whereas the introduction of a standard antioxidant package preserves the performance characteristics at a recycled-PE content of up to ~20% [18,25]. Thus, the use of recyclates in rotational molding is feasible provided that mandatory restabilization and thorough preparation of the material are carried out [18,42,43].

The stabilization of polyethylene with antioxidants is a key factor determining the durability of rotational molding products. For virgin PE, the use of stabilizing additives is mandatory [13,27], whereas recycled PE requires additional restabilization [9,44]. Antioxidants at optimal concentrations give rPE-based compositions mechanical properties and a service life comparable to those of virgin PE [36,45], and the efficiency of stabilization is assessed by thermogravimetric analysis (TGA), differential scanning calorimetry (DSC), the oxidation induction time (OIT), infrared (IR) spectroscopy [20,27,46], ultrasonic flaw detection [47], the melt flow rate (MFR), and mechanical testing [26,27,28,29]. The application of ultrasonic testing is based on the nonlinear acoustic properties of polymer materials [48,49,50,51,52,53,54,55,56]. In contrast to linear ultrasonic methods, which rely on measuring the wave propagation velocity and the attenuation coefficient, nonlinear ultrasonic spectroscopy is substantially more sensitive to the microstructural changes that arise at the early stages of polyethylene degradation and that form during powder sintering and the subsequent cooling of the product [48,49]. Real polymer materials are nonlinear elastic media in which the interatomic and intermolecular interactions show anharmonicity of the bond potential energy, which distorts the shape of the propagating ultrasonic wave and gives rise to higher harmonics in its spectrum [50,53]. It has been shown experimentally that the parameters of the third harmonic are especially sensitive to symmetric changes in the shape of the potential well, which reflect changes in bond stiffness and the redistribution of stresses in the material [51]. Gomes et al. showed that higher-harmonic analysis distinguishes the stages of incomplete sintering, optimal structure formation, and thermo-oxidative degradation of polyethylene, which are practically undetectable by linear ultrasonic flaw-detection methods [48,49,56]. Upon the overheating and oxidation of polyethylene, chemical and structural changes occur, including the formation of crosslinks and oxidized functional groups, increasing the anharmonicity of the elastic response and producing a pronounced rise in the amplitude of the third harmonic of the ultrasonic signal [48,51]. In addition, rotationally molded products carry internal stresses caused by the non-uniform cooling and shrinkage of the material, which alter the local state of the crystal lattice and affect the acoustic response through the acoustoelastic effect [49,52,53,54].

In our previously published works, a correlation was established between the level of internal stresses in the material, its resistance to environmental stress cracking (ESCR), and the height of the third harmonic of the ultrasonic signal at a recycled-polyethylene content of up to 50%. In the present study, the behavior of this parameter is examined as the share of the recycled component is increased to 100% [24].

The aim of the present work is to develop an industrial recycled-polyethylene composite for rotational molding, resistant to outdoor service conditions, by optimizing its composition through the introduction of a package of stabilizing additives [30,39]: a phenolic antioxidant, a phosphite stabilizer, and carbon black as an ultraviolet (UV) absorber. This aim is consistent with the priorities of the industry and cross-industry associations of the Republic of Kazakhstan, including the KazWaste Association, the Union of the Chemical Industry of Kazakhstan, and the relevant committees of the Atameken National Chamber of Entrepreneurs, with respect to the development of secondary polymer recycling, the creation of sustainable composite materials, and the expansion of the industrial application of recycled polyethylene in products for outdoor service. The developed composite material is intended for trial use by the largest manufacturer of agricultural tanks in the Republic of Kazakhstan, AVAGRO LLP.

The combined effect of the rPE content, the pigment concentration, the peak internal air temperature (PIAT), and the stabilizing additives on the properties of the products has a complex character; therefore, the probabilistic–deterministic planning (PDP) method was applied. This approach substantially reduced the number of experiments required and accounted for the interaction of factors, ensuring the targeted optimization of the composite with respect to the threshold values of the product characteristics [57]. In the present work, the impact of five factors varying on five levels was studied. The use of the PDP method (plan of experiments based on an orthogonal Greco–Latin square) reduced the number of experiments from 3125 to 25—that is, by a factor of 125.

## 2. Materials and Methods

### 2.1. Materials

For this study, virgin rotational polyethylene of the Lupolen 4021 KRM grade (hereinafter vPE) with a density of 939.5 kg/m^3^ and a melt flow index (MFI) (190 °C/2.16 kg) of 4 g/10 min from LyondellBasell (Frankfurt am Main, Germany) was used. Recycled polyethylene of the same Lupolen 4021 KRM grade (hereinafter rPE) was obtained from tank lids by crushing without heating, followed by milling at a temperature not exceeding 35 °C. This rPE originated from in-plant process scrap and, therefore, belongs to the post-industrial waste category. It contained no post-consumer recyclate recovered from tanks or other articles that had been in service and had thus been exposed to ultraviolet radiation and chemically aggressive media.

Carbon black (in powder form) of the P803 grade, according to GOST 7885-86 (Tuymazytekhuglerod, Tuymazy, Russia), was used as the pigment. The product ROTOW9042 (MARBO, Pogliano Milanese, Italy) was used as the mold-release agent for sample preparation.

To increase the thermo-oxidative stability of the rotational composites, two types of antioxidants were used:

(1) Phenolic antioxidant of the 1010 grade (CAS No.: 6683-19-8), belonging to the class of sterically hindered phenols—pentaerythritol tetrakis[β-(3,5-di-tert-butyl-4-hydroxyphenyl)propionate], with the formula C_73_H_108_O_12_ and a molecular weight of 1177.65 g/mol. The chemical structural formula is shown in Figure 1a [58].
(2) Phosphite antioxidant of the 168 grade (CAS No.: 31570-04-4)—tris(2,4-di-tert-butylphenyl) phosphite, with the formula C_42_H_63_O_3_P and a molecular weight of 646.922 g/mol. The chemical structural formula is shown in Figure 1b. This 168-grade antioxidant functions as a secondary antioxidant and a processing stabilizer [58].

The antioxidants were introduced into the compositions at the specified concentrations during mixture preparation. The combined use of the phenolic and phosphite antioxidants provided a synergistic stabilization effect through the inhibition of radical processes and the decomposition of hydroperoxides [31,59]. Before processing, all components were subjected to preliminary dosing and homogenization.

### 2.2. Samples Preparation

The composite samples were prepared on a uniaxial rotational machine of the Roto Rocket grade (manufacturer 493K, Ballyclare, UK), using a hexagonal cylindrical metal mold of 130 × 45 mm.

A charge of 80 g of the mixture containing polyethylene (vPE and rPE), pigment, and functional additives in the ratios specified in the experimental plan (Section 2.10) was loaded into the mold, which was then rotated at the maximum heating level available for this model of laboratory machine. Sintering lasted 30–40 min; the internal air temperature of the mold (IAT) was monitored by a thermocouple throughout the entire rotational molding cycle, and its maximum value reached during the heating stage is referred to below as the peak internal air temperature (PIAT), which is used as the technological factor of the experimental design.

After the internal air temperature (IAT) had decreased to 70 °C, the samples were removed from the mold. During rotational molding (heating and cooling), the rotational speed about the main axis (arm speed) was 30 rpm.

The degrees of sintering of rotational polyethylene are classified by a combination of the mechanical and density characteristics of the samples, following the approach proposed in [23,24]:

– Incomplete Sintering (ICS)—Samples obtained at the minimum PIAT (≈170 °C) show insufficient powder coalescence with the retention of microbubbles; the specific mean failure energy MFEsp. < 35 J/mm, and the density < 0.9395 g/cm^3^ (0.928–0.933 g/cm^3^ for rPE composites). The ultrasonic spectrum does not contain a pronounced third harmonic—broadband noise is recorded at a level of ≤ 0.15 ± 0.02 rel. u., and the amplitude of the transmitted signal is minimal.
– Normal Sintering (NS)—Samples obtained at the optimal PIAT (≈195–205 °C, optimum ≈197 °C) exhibit MFEsp. > 40 J/mm and a density ≥ 0.9395 g/cm^3^; the internal stresses are minimal: β = 0.1 ± 0.02 rel. u. at 20–40 °C.
– Thermo-Oxidative Degradation Stage (TDS)—Samples obtained at the maximum PIAT (≈220–240 °C) exhibit thermo-oxidative degradation with the formation of surface carbonyl groups (the 1740 cm^−1^ band), crosslinks, and a sharp increase in internal stresses; MFEsp. drops by ~15% and β rises by ~36% relative to the NS region. The density in this case exceeds 0.9395 g/cm^3^ owing to chemicrystallization following the oxidative breakdown of the amorphous phase.

### 2.3. Ultrasonic Inspection Methods

Ultrasonic measurements were carried out by the mirror-shadow method in accordance with the scheme shown in Figure 2.

This configuration provides for the registration of the transmitted signal after its reflection from the opposite surface of the sample, which ensures the formation of a stable interference pattern and the possibility of spectral analysis of higher harmonics at a fixed geometry of the acoustic path. A USD-60KIM ultrasonic flaw-detection system for the inspection of composites and materials, together with a UCD 60H ultrasonic flaw detector (Kropus, Moscow, Russia), was used as the measuring equipment. Excitation was performed by applying an electrical signal with an amplitude of 200 V, a fill frequency of 3.75 MHz, and a repetition frequency of 20 Hz to the piezoelectric transducer. The ultrasonic beam incidence angle was 65° in order to create the wave propagation and reflection conditions characteristic of the mirror-shadow method in the PE samples. All measurements were carried out at 20 °C, without additional thermal stabilization of the samples.

In contrast to the previously published work [23], where the gain of the receiving path was 60 dB, in the present study the gain was increased to 70 dB. This change was due to the reduction in the thickness of the plastic samples under study from 7.5 mm to 2 mm. According to the relationship for the third-harmonic amplitude [60], given by Equation (1), the third-harmonic amplitude *U*_3_ depends linearly on the propagation length *z*, which in this geometry is determined by the effective thickness of the acoustic path in the material.

(1) $$U_{3}\approx \;\gamma k^{3}U_{1}z$$

where *U*_3_ is the amplitude of the third harmonic of the ultrasonic signal; *U*_1_ is the amplitude of the fundamental (first) harmonic; *k* is the wavenumber, defined as *k* = 2π*f*/*c*, where *f* is the excitation frequency and c is the longitudinal-wave velocity in the material; *z* is the effective wave-propagation length in the sample, determined by the geometry of the mirror-shadow transmission and the actual thickness of the layer under inspection; and *γ* is the cubic-nonlinearity parameter of the medium, characterizing the anharmonicity of the intermolecular potential of the polymer.

As the thickness decreases, *z* decreases, which leads to a proportional decrease in *U*_3_. Given the cubic dependence on *U*_1_ and the initially low level of the nonlinear component, a decrease in z causes a substantial drop in the registered level of *U*_3_ relative to the noise floor. Increasing the gain to 70 dB is necessary to compensate for this decrease and to ensure stable registration of the spectral maxima of the higher harmonics at the new material thickness.

Within the ultrasonic studies, three parameters were determined: the amplitude of the transmitted signal A in the time domain (not a spectral acoustic parameter), the amplitude coefficient of the third harmonic (*β*), and the amplitude coefficient of the fifth harmonic (*λ*). The spectrum was registered over the built-in frequency range of the flaw detector, and the amplitudes of the harmonic components were estimated using the coordinate grid of the display in relative units of amplitude (rel. u.). Along the amplitude axis Oy, 10 cells are shown on the screen; each cell contains 5 divisions, and the step of one division is 0.02 rel. u. To unify the measurement conditions, the gain of the receiving path was set so that the amplitude of the fundamental harmonic *U*_1_ stably occupied 10 cells (50 divisions) of the spectrometric scale. The amplitudes of the nonlinear components were then determined by directly counting the number of divisions along the vertical scale within the corresponding frequency windows: *U*_3_ was recorded as the maximum of the spectral peak in the 7.8–13.5 MHz range, and *U*_5_ as the maximum of the spectral peak in the 16.5–20.5 MHz range. The parameter *β* was determined by Equation (2):

(2) $$\beta =\frac{U_{3}}{U_{1}}$$

In a similar manner, the parameter *λ* was calculated in accordance with Equation (3):

(3) $$\lambda =\frac{U_{5}}{U_{1}}$$

The use of small-step amplitude sampling is fundamentally important for the correct estimation of the weak nonlinear components, since *β* and especially *λ* have a level significantly lower than that of the fundamental component.

### 2.4. Mechanical Testing Method

The hexagonal cylindrical samples manufactured on the Roto Rocket laboratory rotational machine were cut into flat plates, from which dumbbell specimens of ISO 527-2 type 1B [61] (overall length 150 mm, gauge width 10 mm) were machined for tensile testing. The specimens were used to determine the tensile modulus of elasticity (E, MPa), the tensile yield stress (σyield, MPa), and the tensile strength (σbreak, MPa) in accordance with ISO 527-2:2012 at (20 ± 1) °C and a crosshead speed of 1 mm/min on a Haida International Equipment HD-B617-S machine (Dongguan, China). Compared with our earlier study on the same equipment [24], the crosshead speed was reduced so that the tensile modulus was determined in the low-strain-rate region prescribed by ISO 527-1 [62] and the yield region was resolved with better resolution. Before testing, the specimens were conditioned for 24 h at (20 ± 1) °C—that is, at the test temperature—in a temperature-controlled cabinet placed in a climate-controlled room. The reported modulus and strength values are based on the average of no fewer than 18 samples.

Similar plates were used to assess the impact strength of the material. The mean failure energy (MFE) was determined in accordance with the Association of Rotational Molders (ARM) International Low-Temperature Impact Test [63] at (20 ± 1) °C. The specimens for the determination of MFE were conditioned under the same regime: 24 h at (20 ± 1) °C. The reported *MFE_sp_*. values are based on the average of no fewer than ten samples, are expressed in J/mm, and were calculated by Equation (4):

(4) $$MFE_{sp}=\;\frac{MFE}{h}$$

where *h* is the sample thickness, in mm.

*MFE_sp_*—specific mean failure energy, J/mm;

*MFE*—mean failure energy, J.

Sample density was measured based on the Archimedes method as described in International Organization for Standardization (ISO) 1183 on an MD-300S electronic densimeter (Osaka, Japan) (at room temperature in distilled water).

### 2.5. Contact Angle Measurement Method

The contact angle of wettability θ° was determined on an ACAM instrument (manufacturer Apex Instruments, Kolkata, India) using the standard software “Apex Acam Software”, version 2.026.088.1. The composite sample under test was leveled with the baseline in the field of view of the camera. A drop of distilled water was carefully formed with a dispenser above the sample surface and deposited onto the substrate, avoiding inertial spreading. After the drop shape had stabilized, the side profile was photographed. The calculation of θ was carried out by the “elliptical method”: in the program interface, reference points were placed manually on the contour of the drop profile (an arbitrary number, but no fewer than eight), after which the software approximated the profile with an ellipse and automatically calculated the contact angles as the angles of the tangents to the approximating curve at the points of the three-phase line. For each position, the left and right angles were recorded, and the program output the average value for the given drop imprint. Measurements were carried out at three different locations on each sample; at each point, three independent drop depositions and angle calculations were performed. The values reported are averaged over three measurements at three points (nine determinations per sample in total). The approach used is the sessile-drop method, with elliptical approximation of the profile and calculation of θ from the tangent at the point of triple contact.

### 2.6. Fourier-Transform Infrared Spectroscopy Method

Fourier-transform infrared (FTIR) spectra were obtained using a spectrometer based on the SpiritX model (manufacturer Shimadzu, Kyoto, Japan), operating in the 400–4000 cm^−1^ range, with 25 scans and a resolution of 4 cm^−1^ [64]. The IR spectra were recorded at M. Kozybayev North Kazakhstan University in the “Water Quality Assessment” laboratory.

All indices, including the comparative ones, were calculated from the IR spectrum on the absorbance scale A(v), where v is the wavenumber (cm^−1^).

The carbonyl index (CI, denoted CIS in Equation (5)), the hydroxyl index (OI, OHh in Equation (6)), and the crystallinity index (CRYST, CR730/718 in Equation (7)) were selected because together they resolve three chemically distinct consequences of polyethylene oxidation—the formation of chain-scission carbonyl products, the accumulation of hydroxyl and hydroperoxide species, and the redistribution between the crystalline and amorphous phases—none of which can be separated by a single index [64,65]. For recyclate-containing systems, this combination is essential: rPE enters the process with a finite initial oxidation level, so that the informative quantity is the increment of each index; the hydroperoxide functionality accumulated in previous service governs the re-initiation of oxidation and is invisible to CI; and CRYST resolves chemicrystallization, thereby distinguishing embrittlement by chain scission from embrittlement by densification. All three indices are derived from a single ATR-FTIR spectrum—that is, from an identical probed volume of 1–2 µm depth, which corresponds to the surface-localized character of photo-oxidation in a 2 mm rotationally molded wall. The spectral windows used were 1650–1850 cm^−1^ (ν(C=O)), 3100–3600 cm^−1^ (ν(O–H)), and the 730/718 cm^−1^ ρ(CH_2_) doublet, referenced to the 1420–1500 cm^−1^ δ(CH_2_) band.

#### 2.6.1. Carbonyl Index (CI)

The carbonyl index quantifies the relative content of C=O carbonyl groups—products of the oxidative degradation of the polyolefin (ketones, aldehydes, carboxylic acids, esters, lactones) [64,65]. It is calculated by the formula

(5) $${CI}_{S}=\frac{\int_{1650}^{1850}A(\nu )\;d\nu }{\int_{1420}^{1500}A(\nu )\;d\nu }$$

where $\int_{1650}^{1850}A(\nu )\;d\nu$ is the area under the C=O stretching-vibration band (1650–1850 cm^−1^). It captures all types of carbonyls formed upon oxidation.

$\int_{1420}^{1500}A(\nu )\;d\nu$ is the area of the δ(CH_2_) scissoring-deformation band (1420–1500 cm^−1^). It is used as an internal standard: it is proportional to the mass of the polymer matrix in the probed zone and compensates for differences in sample thickness and in contact with the attenuated-total-reflectance (ATR) crystal.

The integration was performed by the trapezoidal method over the experimental points after baseline correction.

In interpretation, an increase in CI indicates progressing thermo-oxidative degradation.

#### 2.6.2. Hydroxyl Index (OI)

The hydroxyl index quantifies the content of OH groups: alcohols, hydroperoxides, carboxylic acids, and sorbed water—all polar products that give O–H stretching vibrations.

It is calculated by the formula

(6) $$OI=\frac{\max_{3100\;\leq \nu \leq \;3600}A(\nu )}{A(1465)}$$

where $\max_{3100\;\leq \nu \leq \;3600}A(\nu )$ is the absorption maximum in the region of O–H stretching vibrations (3100–3600 cm^−1^). The maximum, rather than the integral, is taken in order to remove the contribution of the broad low-intensity “tails” from hydrogen bonds.

*A*(1465) is the height of the δ(CH_2_) scissoring-deformation peak at 1465 cm^−1^ relative to the local baseline.

In interpretation, an increase in OI indicates the accumulation of OH-containing oxidation products and/or an increase in water absorption.

#### 2.6.3. Crystallinity Index (${CR}_{730/718}$)

This represents the ratio of the crystalline and amorphous phases of polyethylene. The ρ(CH_2_) rocking vibrations in polyethylene, in the crystalline orthorhombic lattice, split into a 730/720 cm^−1^ doublet owing to the intermolecular interaction of the two macromolecules in the unit cell; in the amorphous phase, the splitting is absent and a single component near 718–720 cm^−1^ is retained.

It is calculated by the formula

(7) $${CR}_{730/718}=\frac{A(730)}{A(718)}$$

where *A*(730) is the height of the 730 cm^−1^ peak relative to the local baseline (a ±4 cm^−1^ window for noise robustness). It is the “crystalline” component of the doublet, which appears only upon the ordered packing of chains.

*A*(718) is the height of the 718 cm^−1^ peak in a similar window. It contains the contribution of both the crystalline component of the doublet and the amorphous band; in purely amorphous PE, only the latter remains.

In interpretation, an increase in ${CR}_{730/718}$ indicates an increase in the degree of crystallinity (for example, upon annealing or chemicrystallization following the oxidative breakdown of the binding components of the amorphous phase) [66].

CRYST is a relative spectroscopic measure of the crystalline-to-amorphous ratio within this surface layer and not an absolute degree of crystallinity; absolute values would require X-ray diffraction (XRD), whose conventional Bragg–Brentano penetration depth of tens to hundreds of micrometers would be dominated by the essentially unmodified bulk of the wall, and depth-resolved XRD is therefore identified in Section 4 as the independent structural verification of the present findings.

### 2.7. Method of Thermal Analysis

Thermal analysis was performed using a Synchronous Thermal Analyzer thermogravimetric analyzer, model DZ-STA200, Ser. No. 2025112902 (manufacturer Dongguan ZONHOW Test Equipment Co., Ltd., Dongguan, China).

The measurements were carried out in the standard differential thermal analysis–thermogravimetry (DTA–TG) mode. A ceramic container with high chemical and thermal resistance was used as the crucible. The experiment was carried out in an air atmosphere. The thermogravimetric analysis was performed in accordance with ASTM E1131 [67] at a heating rate of 20 °C/min over the range from 20 °C to 900 °C, using samples with a mass of 11.92–21.42 mg (on average, 17.29 ± 2.69 mg).

The following characteristics were determined from the results of the thermal analysis:

– The onset temperature of thermal degradation T_b_ (°C);
– The temperature of maximum mass loss (T_max_, °C);
– The mass loss of the sample (M, %).

### 2.8. Method of UV Testing of PE Samples for Weather Resistance

Accelerated weather-resistance testing of the rotational PE samples was carried out in a UV-aging chamber, model DZZY-D (manufacturer—Dongguan Zonhow Test Equipment Co., Ltd., Dongguan, China), equipped with eight fluorescent UV lamps of the UVB-313EL/59A type (Q-Lab, Westlake, OH, USA; spectral class per Q-Lab Technical Bulletin LU-8160 [68]) (peak 313 nm, range 280–315 nm), each with a power of 40 W (lamp service life—2000 h). The chamber is intended to simulate the effect of solar ultraviolet radiation, temperature, and moisture on polymer materials and complies with the requirements of the ASTM G154, ISO 4892-3, ASTM D4329 [69], ASTM D4587 [70], and SAE J2020 [71] standards.

Test samples—plates of 130 × 45 mm in size, with a thickness of 2–3 mm—were placed inside the working chamber. Before exposure, the specimens were washed with distilled water and then held for 24 h at (20 ± 1) °C in the same temperature-controlled cabinet described in Section 2.4. This step removes residual moisture after washing and brings all specimens to the same initial surface state, so that the reference infrared spectra recorded before exposure are not affected by uneven drying. The samples were placed in the chamber facing the lamps with the outer surface of the rotationally molded product (the side facing the sun during service).

The tests were carried out in accordance with ASTM G154 (“Standard Practice for Operating Fluorescent Ultraviolet (UV) Lamp Apparatus for Exposure of Materials”) [72] at an irradiance of 0.71 W/m^2^ and a control wavelength of 313 nm, following the recommendations of Q-Lab Corporation, with the black-panel temperature controlled at 60 ± 3 °C during UV irradiation and 50 ± 3 °C during condensation (see Table 1), with repeating cycles alternating two different stages.

Each test cycle consisted of two stages:

The cycles were repeated continuously in automatic mode until the specified test duration was reached. The irradiance was monitored by the built-in radiometer of the chamber.

The test duration and the sampling intervals for the assessment of degradation were chosen on the basis of the effective acceleration factor $K_{eff}$, calculated for the climatic conditions of Northern Kazakhstan within a conditional kinetic model that takes into account the photochemical component of the UVB exposure and the temperature acceleration of the oxidative degradation of polyethylene according to the Arrhenius equation.

The photochemical factor $K_{UV}$ for the climatic conditions of Northern Kazakhstan was taken to be ≈30. As an approximate industry benchmark for accelerated UVB testing, it was assumed that about 500 h of cyclic UVB-313 exposure in the ASTM G154 mode (irradiance 0.71 W/m^2^ at a control wavelength of 313 nm; cycle of 8 h UV + 4 h condensation) corresponds to one year of outdoor exposure under conditions of high solar load of the South Florida type (dimensionless factor 8760/500 ≈ 17.5). This value was used not as a universal standard factor but as an initial estimate for the construction of the conditional kinetic model. The climatic correction for the annual integral UV dose for the transition Miami → Petropavlovsk (≈1.87 according to data from the World Radiation Data Centre and SURFRAD [73] for a latitude of 54° N) gives $K_{UV}$ ≈ 17.5 × 1.87 ≈ 32.7 for the climate of Northern Kazakhstan; in the further calculations, a conservative value rounded down to $K_{UV}$ ≈ 30 was adopted.

The temperature correction $K_{T}$ was calculated by the Arrhenius equation:

(8) $$K_{T}\;=\;exp\left[\left(\frac{E_{a}}{R}\right)\;\cdot \;\left(\frac{1}{T_{out}}\;-\;\frac{1}{T_{lab}}\right)\right]\times \;(1/T_{out}-\;1/T_{lab}),$$

where $E_{a}$ = 30 kJ/mol is a conservative lower estimate of the activation energy of the combined secondary stages of the photo-oxidative degradation of polyethylene (the literature range is 20–95 kJ/mol for high-density and low-density polyethylene (HDPE/LDPE), depending on the degradation marker); $T_{lab}$ = 333 K corresponds to the black-panel temperature of 60 °C in the UV phase of the test (Table 1); $T_{out}$ = 293 K corresponds to the UV-weighted surface temperature of the material in outdoor service at a latitude of 54° N (averaged over the hours with UVI > 1, weighted by the UVB irradiance; for Northern Kazakhstan, summer values of 35–45 °C under daytime insolation and winter values of about −5…−15 °C give an effective weighted average of ≈20 °C). Under these assumptions,

$$K_{T}=exp\left[\left(\frac{30000}{8.314}\right)\;\cdot \;\left(\frac{1}{293}-\frac{1}{333}\right)\right]\;\approx \;4.4.$$

The final value is $K_{eff}\;=\;K_{UV}\;\cdot \;K_{T}\;\approx \;30\;\cdot \;4.4\;\approx \;132$. The conditional degradation horizon was calculated by the following expression:

(9) $$t_{cond}\;=\;t_{camera}\;\cdot \;\frac{K_{eff}}{8760},$$

where $t_{camera}$ is the total duration of cyclic exposure in the chamber (h) and 8760 is the number of hours in a year. Accordingly, cyclic exposures of 333 and 666 h in the UV chamber form degradation states conditionally correlated with 5- and 10-year service horizons for the climatic conditions of Northern Kazakhstan. For other regions (Almaty, Astana, Shymkent), $K_{eff}$ was recalculated by direct scaling of the base factor 17.5 with respect to the ratio of the annual UV doses of Miami and the corresponding location, and by multiplication by $K_{T}$ = 4.4 (which increases as the average annual natural UV load decreases), without the additional rounding applied for Northern Kazakhstan; the values are given in Table 2.

This comparison is not a direct calendar conversion of the laboratory test time into the service life of the product. The values “5 years” and “10 years” are used as conditional service horizons for the interpretation of the degradation response. Accelerated UVB aging according to ASTM G154 [72] and ISO 4892-3:2024 [74] predominantly reproduces the photo-oxidative, thermal, and hydrothermal components of atmospheric exposure, but not the whole complex of factors of real service (dust erosion, thermomechanical loads, biodegradation, freeze–thaw cycles). The spectrum of the UVB-313 lamps is harsher than the solar one (wavelengths < 295 nm are present that are absent in the solar spectrum at the Earth’s surface); therefore, $K_{eff}$ is applicable for assessing the relative photochemical resistance of the compositions and for forming a predictive degradation horizon, rather than for a literal conversion of “laboratory hours → years of service.”

Correspondence between the duration of exposure in the UV chamber and the conditional service horizon for four climatic locations of Kazakhstan was calculated by the effective acceleration factor $K_{eff}$ = $K_{UV}$ × $K_{T}$ (see Section 2.8). $K_{T}$ = 4.4 was adopted as common for all locations; $K_{UV}$ for each location was recalculated taking into account the annual integral UV dose of the corresponding climatic location and increases as the average annual natural UV load decreases, since the same laboratory dose corresponds to a larger number of years of outdoor exposure in regions with lower insolation.

After exposure of the PE samples to the cyclic UVB-aging mode for 333 and 666 h of total cyclic exposure (including the UV-irradiation and condensation phases, at a ratio of 8:4) (reference documents: Q-Lab Technical Bulletin LU-8160 [68] and ISO 4892-3:2024 [74]), the height of the third harmonic of the ultrasonic signal, the impact strength in terms of the MFE parameter (J/mm), and the IR spectral characteristics (of all 25 samples) were determined. Within the conditional kinetic model $K_{eff}$ = $K_{UV}$ × $K_{T}$ ≈ 30 × 4.4 ≈ 132 (see Section 2.8), the indicated exposure levels form degradation states correlated with 5- and 10-year service horizons for the climatic conditions of Northern Kazakhstan. This comparison is not a direct calendar conversion of laboratory hours into the service life of the product but is used for the interpretation of the degradation response of the polyethylene compositions.

### 2.9. Porosity Counting Method

Samples were cut from the flat walls of the rotationally molded products at room temperature in a single pass with a sharp blade; sawing and milling were avoided in order to limit smearing of the pore edges. The fresh-cut surfaces were examined without additional polishing, in order to preserve the natural morphology of the pores. For each composition, three non-overlapping fields of view with a resolution of 1280 × 960 pixels were registered using a digital optical microscope in reflected light under constant illumination. Pore detection and sizing were performed on digital images, whose native unit is the pixel; the absolute metric scale of the images was not determined, and pore sizes are therefore reported in pixels as relative values. Comparisons between compositions—ratios, trends, and optimal positions—are independent of the conversion factor [75].

Pore detection was performed using an in-house program, “Pore Counter” (Python 3, OpenCV 4); a general view of the interface is shown in Figure 3 [75].

Each image is automatically classified as “gray” or “green” on the basis of the dominance of the green channel and the share of pixels in the green HSV range, after which it is directed into one of two pipelines. Gray Pipeline: 7 × 7 median filtering, background estimation by large-kernel Gaussian blurring, background subtraction, Otsu binarization with a contrast-dependent threshold correction, and morphological opening and closing operations. Green Pipeline: Preliminary contrast correction by CLAHE (clip = 4.0) and two-pass detection: (i) the circular Hough transform (HOUGH_GRADIENT_ALT) with verification of the angular uniformity of the edge and non-maximum suppression; (ii) backup detection by background subtraction for non-circular pores, with the merging of masks by an OR operation. In both pipelines, morphometric filtering of the contours is applied: a minimum area of 60 px^2^ (gray channel)/250 px^2^ (pigmented), circularity ≥ 0.30, solidity ≥ 0.40, aspect ratio ≤ 3.0, and an individual area ≤ 8% of the image area; regions touching the frame boundary are not excluded. The built-in graphical interface allows the masks to be reviewed and corrected manually; the original and corrected masks are saved separately to ensure traceability.

The porosity of the polyethylene samples was described by the following quantitative parameters:

(1) The surface porosity P (mean porosity, %) was calculated by Equation (10):

P = S_pore_/S_image_ × 100% (10)

where S_pore_ is the total area of all detected pores in the image, µm^2^;

S_image_ is the total area of the analyzed image, µm^2^.

(2) The equivalent pore diameter d_eq_ (µm) was calculated by Equation (11):

d_eq_ = √(4 S_i_/π) (11)

where S_i_ is the area of the i-th pore in the image, µm^2^;

i is the ordinal number of the detected pore (i = 1, 2, …, N, where N is the total number of pores in the image);

π is the mathematical constant (the ratio of the circumference of a circle to its diameter).

(3) The mean number of pores N is the number of pores detected in the analyzed field of view with an area of S_image_.

The reported porosity values are the arithmetic mean over *n* = 3 non-overlapping fields of view for each composition; the error was estimated as the standard deviation over these three measurements.

### 2.10. Experimental Design

The modeling of the combined effect of the contents of recycled rotational polyethylene (rPE, %), pigment (Cp, %), and antioxidants (CAO, %), the antioxidant ratio (RAO, dimensionless), and the technological parameter (PIAT, °C) on the mechanical, thermal, and acoustic properties of the composites—the height of the third (β, rel. u.) and fifth (λ, rel. u.) harmonics, the amplitude (A) of the ultrasonic signal, the impact strength (MFEsp., J/mm), the contact angle of wettability (θ°), and the environmental stress cracking resistance (ESCR)—was carried out by the probabilistic–deterministic planning (PDP) method. A detailed description of the PDP method is given in [57]. A five-factor experimental plan at five levels, based on a Greco–Latin square, was developed for the study. If we consider all possible combinations of input factor levels based on a 5 × 5 × 5 × 5 × 5 plan of experiments, this will require conducting 3125 experiments. However, if we use an experimental plan based on an orthogonal Greco–Latin square, only 25 experiments will be sufficient.

When modeling by the PDP method, the following actions were performed:

1. The following factors were defined as input parameters:

– The recycled-polyethylene content (rPE) took the values 0, 25, 50, 75, and 100%;
– The pigment content (Cp) took the values 0, 0.1, 0.2, 0.5, and 1%;
– The antioxidant content (CAO, %): 0, 0.25, 0.5, 0.75, and 1%;
– The antioxidant ratio (RAO), i.e., the ratio of the mass of antioxidant 1010 to antioxidant 168: 0.33, 0.5, 1, 2, and 3;
– The PIAT value took the values 160, 180, 200, 220, and 240 °C.

2. Next, a five-factor experimental plan at five levels was developed. Table 3 presents the plan of experiments and the results of the experimental determination of the characteristics of the rotational composites.

## 3. Results

### 3.1. Ultrasonic Signal Characteristics

The influence of the contents of rPE, Cp, CAO, as well as RAO and the process parameter PIAT, on the ultrasonic parameters β, A, and λ is presented in Figure 4, Figure 5 and Figure 6, respectively.

The results of the study of the ultrasonic characteristics identify the regions of structural stability of the material upon variation in all of the key factors. The established dependences of the spectral characteristics β and of the time parameter A on the contents of rPE and Cp, the concentration of CAO, the ratio RAO, and the value of PIAT determine the optimal technological regimes. Specifically, for products without UV exposure, the acoustic response curves indicate the following optimal values: PIAT ≈ 200 °C, Cp ≈ 0.1–0.2%, CAO ≈ 0.25–0.5%, and RAO = 0.5–1.0, yielding β = 0.076–0.09 rel. u., MFE ≈ 2.6 J/mm, and a porosity of 2.5–3%. The coincidence of the extrema in the plots for β, λ, and A confirms the high sensitivity of the spectral analysis of higher harmonics to changes in the anharmonicity of the intermolecular potential of the polymer.

### 3.2. Mechanical Properties

The influence of rPE content, pigment content, PIAT, content of antioxidants, and the ratio of antioxidants on the maximum tensile stress at break is shown in Figure 7.

The influence of rPE content, pigment content, PIAT, content of antioxidants, and the ratio of antioxidants on the tensile yield stress at yield is shown in Figure 8.

The influence of rPE content, pigment content, PIAT, content of antioxidants, and the ratio of antioxidants on the elongation at break is shown in Figure 9.

The influence of rPE content, pigment content, PIAT, content of antioxidants, and the ratio of antioxidants on the elastic modulus is shown in Figure 10.

The influence of the studied optimization parameters on the mechanical properties of the rotationally molded composites is not straightforward. In general, as the rPE content in the composite increases, the tensile strength, the yield strength, and the elongation at break decrease and stabilize (at rPE contents above 75%). As the pigment content increases, the tensile strength rises up to C_p_ = 0.3%, then falls up to 0.8%, and then levels off; the yield strength rises up to 0.3% and then falls; the elongation at break falls up to 0.3%, remains constant up to 0.75%, and then falls again. With increasing antioxidant content, tensile strength rises to 0.25%, decreases slightly to 0.75%, and then rises above 0.75%; yield strength is stable and decreases above 0.75%; elongation at break increases to 0.25%, drops to 0.75, and then stabilizes. With an increase in the content of antioxidant 1010 in relation to antioxidant 168, tensile strength decreases and stabilizes at a ratio above 2; yield strength decreases and stabilizes above a ratio of 1.5; elongation at break increases up to a ratio of 1, and then decreases and stabilizes at a ratio above 2.

### 3.3. Impact Strength

The influence of rPE content, pigment (Cp), antioxidant content (*C_AO_*), the process parameter PIAT, and antioxidant ratio (*R_AO_*) on the specific mean failure energy (MFEsp., J/mm) is shown in Figure 11.

An increase in the recycled-polyethylene content up to 50% causes a 20% decrease in the impact strength of the composites (as judged by the MFEsp. parameter). However, upon a further increase in the rPE content (from 50 to 100%), the impact strength of the composites stabilizes. The introduction of 0.1% pigment causes a local increase in MFEsp., while with a further increase in its content (from 0.1 to 1%) it stabilizes at the normal level. The influence of PIAT on MFEsp. (Figure 11c) has a classical character and has been described in a large number of works [12,13,15,23]. The antioxidants increase the impact strength to a similar extent across the studied range of the composites, which may be associated with the suppression of thermo-oxidative degradation and the cessation of PE chain scission. An increase in RAO (the ratio of the phenolic and phosphite antioxidants) from 0.33 to 1 causes a small increase in MFEsp.; however, with an increase in RAO from 1 to 2, a sharp decrease in MFEsp. of 15% is observed.

### 3.4. Wettability (Contact Angle θ)

The influence of rPE content, pigment content, the process parameter PIAT, content of antioxidants, and the ratio of antioxidants on the contact angle of wettability (θ) is shown in Figure 12 and Figure 13.

An analysis of the plots shows the influence of the technological parameters on the contact angle of wettability (θ) of the composites. It was established that the θ values in all cases lie in the range of approximately 84–95°, which indicates relatively stable surface properties of the materials. The most noticeable influence on the wettability is exerted by the rPE content and the PIAT temperature, for which small fluctuations in the contact angle are observed. By contrast, the pigment and antioxidant contents have practically no effect on θ. A change in the antioxidant ratio leads only to insignificant changes in the contact angle. Overall, the results show that the parameters under study have a limited influence on the wettability of the composite surface.

### 3.5. Density

The experimentally determined density of the samples is shown in Figure 14.

Samples molded at the low PIAT of 160 °C showed the lowest density (0.922–0.929g/cm^3^). Samples molded at a normal PIAT (180; 200 and 220 °C) or high PIAT (240 °C) attained higher densities (0.938–0.950 g/cm^3^) and are considered acceptable for industrial use.

### 3.6. IR Spectroscopy of the Obtained Samples

The obtained IR spectra of the samples are illustrated in Figure 15.

All twenty-five composite samples showed a common set of absorption bands in the IR spectra, typical of polyethylene and its oxidized derivatives: 3600–3100 cm^−1^—a broad band of O–H stretching vibrations in the hydroxyl and hydroperoxide groups (–OH, –OOH) of the oxidized fragments of polyethylene [64]; 1850–1650 cm^−1^—the general carbonyl region (C=O) of the products of thermal and photo-oxidation, combining the contributions of acids, ketones, aldehydes, and esters [64,65]; 1785–1760 cm^−1^—the high-frequency shoulder of the general carbonyl region, attributed to γ-lactones and peroxycarbonyl fragments; 1750–1735 cm^−1^—ester carbonyl groups (C=O) [76]; 1720–1710 cm^−1^—ketone carbonyl groups (C=O) in oxidized polyethylene [65]; 1500–1420 cm^−1^—the δ(CH_2_) scissoring deformation vibrations of the polyethylene matrix, used in the present work as the reference band in the calculation of the oxidation indices (HI, COI) [66]; 1200–1000 cm^−1^—C–O / C–O–C stretching vibrations in the oxidized fragments of polyethylene (alcohols, ethers, esters, hydroperoxides) [20]; 990–880 cm^−1^—out-of-plane =CH deformation vibrations of vinylene and vinylidene groups (indicators of unsaturation) [15,64]; 730 and 718 cm^−1^—ρ(CH_2_) rocking vibrations; the 730/718 cm^−1^ doublet was used to assess the relative crystallinity index of PE [66].

### 3.7. Thermal Properties

The obtained TG thermogram of the twenty-five investigated samples is presented in Figure 16.

The influence of rPE content, pigment content, the process parameter PIAT, content of antioxidants, and the ratio of antioxidants on the thermal properties of the composites is shown in Figure 17.

The influence of the optimization parameters on the thermal properties of the composites is nonlinear. An increase in the recycled-polyethylene content raises the onset (Tb) and peak (Tmax) degradation temperatures and also reduces the total mass loss M. An increase in the pigment content raises Tb and leads to an initial decrease in M, which then increases at higher pigment concentrations. The higher the PIAT value, the higher the thermal stability of the composites.

### 3.8. Porosity

In the course of the study, the dependences of the porosity characteristics (the mean number of pores, the mean porosity, and the mean pore diameter) on the composition of the composites and on PIAT were determined. The analysis was performed using the “Pore Counter” program on micrographs of cross-sections of the samples [77].

#### 3.8.1. Influence of the Recycled-Polyethylene (rPE) Content

Figure 18 presents the dependences of the porosity characteristics on the recycled-polyethylene (rPE) content in the composite over the range of 0–100%. With an increase in the rPE content, the mean pore count decreases from 55.5 (at 0% rPE) to a minimum of 26.3 (at 75%), after which it rises to 43.1 at 100% rPE. The mean porosity fluctuates within 2.4–4.5% without a pronounced monotonic trend. The mean pore diameter decreases from 31.2 px (at 0%) to 16.9–19.9 px at an rPE content of 74–100% [77].

#### 3.8.2. Influence of PIAT

Figure 19 shows the dependences of the porosity characteristics on the peak internal air temperature (PIAT) over the range of 160–240 °C. The number of pores decreases from 60.8 at 160 °C to 32.3 at 200 °C, then rises to 55.6 at 220 °C, and drops sharply to 20.0 at 240 °C. The porosity decreases from 6.2% (160 °C) to 1.6% (240 °C), demonstrating a general downward trend. The pore diameter varies non-monotonically within 16.6–27.9 px.

#### 3.8.3. Influence of the Pigment Content

Figure 20 presents the dependences on the pigment content Cp (0–1.0%). The number of pores increases from 18.3 (at 0%) to a maximum of 73.1 (at 0.5%), after which it decreases to 39.8 at 1.0%. The porosity changes non-monotonically over the range of 2.8–4.9%. The mean pore diameter decreases sharply from 41.2 px (without pigment) to 15.2–19.5 px upon the addition of even 0.1% pigment, which indicates a refinement of the pores upon the introduction of Cp.

#### 3.8.4. Influence of the Antioxidant Content

Figure 21 shows the dependences on the antioxidant content CAO (0–1.0%). The number of pores remains practically unchanged in the range of 34–38 at CAO from 0 to 0.5% but rises sharply to 60.9 at 0.75%, and then decreases to 34.3 at 1.0%. The porosity varies within a narrow range of 3.1–4.2%. The pore diameter reaches a maximum of 27.7 px at CAO = 0.5%, and upon a further increase in concentration it decreases to 18.6 px.

#### 3.8.5. Influence of the Antioxidant/Stabilizer Ratio

Figure 22 presents the dependences on the CAO/RAO ratio (0.33–3.0). The number of pores increases from 23.3 (CAO/RAO = 0.33) to 67.4 (CAO/RAO = 2.0), and then decreases to 37.5 at CAO/RAO = 3.0. The porosity fluctuates non-monotonically within 2.9–4.8%. The mean pore diameter remains relatively stable (21.2–25.1 px) over the whole range of ratios.

### 3.9. UV Resistance

The influence of the rPE content, the pigment content, PIAT, the antioxidant content, and the antioxidant ratio on the height of the third harmonic after 333 and 666 h of holding in the UV chamber is shown in Figure 23.

The influence of the rPE content, the pigment content, PIAT, the antioxidant content, and the antioxidant ratio on the impact strength (in terms of the MFE parameter, J/mm) after 333 and 666 h of holding in the UV chamber is shown in Figure 24.

After holding in the UV chamber, IR spectra were recorded from the outer PE samples, and three indices were calculated from them: the carbonyl index ${\;CI}_{s}$ (I), the hydroxyl index ${OH}_{h}$ (II), and the crystallinity index ${CR}_{730/718}$ (III), as presented in Figure 25.

The influence of the rPE content (a), the antioxidant concentration CAO (b), and the antioxidant ratio RAO (c) on the carbonyl index ${CI}_{s}$ (I), the hydroxyl index ${OH}_{h}$ (II), and the crystallinity index ${CR}_{730/718}$ (III) is presented in Figure 25.

The test results show that, as the holding time of the PE samples in the UV chamber increases, the height of the third harmonic, CI_integral_area, and OI_3100_3600 increase, while MFE and CRYST_730_718 decrease. Thus, the maximum values of β are recorded at a holding time of 666 h. Consequently, upon holding under atmospheric conditions, the internal stresses in the composites increase.

### 3.10. Development of a Mathematical Model

At the request of the technological service of AVAGRO LLP—the partner organization of this study—and taking into account the main parameters that determine the service life of plastic tanks—the impact strength (the MFE parameter) and the internal stresses in the composites (an indirect indicator of which is the height of the third harmonic of the ultrasonic signal (β))—multifactor mathematical models were developed. These models predict MFE and β at given values of the optimization parameters (rPE, Cp, PIAT, CAO, and RAO). The experimental data for each combination of input factors (see Table 3) were represented as response surfaces in accordance with the standard procedures of experimental design [57,78]. As a result, partial-dependence curves were obtained, as shown in Figure 4, Figure 5, Figure 6, Figure 7, Figure 8, Figure 9, Figure 10, Figure 11, Figure 12, Figure 13 and Figure 14.

To derive a multifactorial statistical mathematical model of the effects of rPE, pigment content (C_p._), antioxidant content (*C_AO_*), antioxidant ratio (*R_AO_*), and PIAT, the generalized equation proposed by M. M. Protodiakonov was used [57]:

(12) $$Y_{0}=\frac{\prod_{i=1}^{n}Y_{i}}{Y_{M}^{n-1}}$$

where $Y_{0}$ is the generalized equation, $Y_{i}$ is the particular function, $\prod_{i=1}^{n}Y_{i}$ is the product of all particular functions, *n* is the number of particular functions equal to the number of input parameters, and $Y_{M}^{n-1}$ is the total average of all of the considered values of the generalized function to a degree one less than the number of particular functions.

Using the obtained partial dependencies, shown in Figure 6 and Figure 11, multifactor mathematical models (13) and (14) were built based on the generalized Protodiakonov Equation (12).

The influence of the input parameters on the value of β is represented as a system of equations:

(13) $$\left\{\begin{array}{l} \beta =\beta \left(rPE\right)\cdot \beta \left(PIAT\right)\cdot \beta \left(Cp\right)\cdot \beta \left(C_{AO}\right)\cdot \beta \left(R_{AO}\right)/\beta_{M}^{4}\\ \beta \left(rPE\right)=-0.00000067\cdot rPE^{3}+0.0001\cdot rPE^{2}-0.0037\cdot rPE+0.115\\ \beta \left(PIAT\right)=-0.000023\cdot PIAT^{2}+0.0105\cdot PIAT-1.064\\ \beta \left(Cp\right)=-0.1584\cdot Cp^{2}+0.1812\cdot Cp+0.0732\\ \beta \left(C_{AO}\right)=0.04\cdot C_{AO}+0.0772\\ \beta \left(R_{AO}\right)=0.08+0.03\cdot {\left(R_{AO}-2.2\right)}^{2}-0.106\cdot e^{-19\cdot {\left(R_{AO}-0.51\right)}^{2}}\\ \beta_{M}=0.0972 \end{array}\right.$$

The correlation coefficient is R = 0.76. The significance of the correlation coefficient is confirmed by the Fisher criterion.

The influence of the input parameters on the value of MFE is represented as a system of equations:

(14) $$\left\{\begin{array}{l} MFE=MFE\left(rPE\right)\cdot MFE\left(PIAT\right)\cdot MFE\left(Cp\right)\cdot MFE\left(C_{AO}\right)\cdot MFE\left(R_{AO}\right)/MFE_{M}^{4}\\ MFE\left(rPE\right)=2.6-0.003\cdot rPE-0.4\cdot e^{-1\cdot {\left(rPE-50\right)}^{2}}\\ MFE\left(PIAT\right)=-0.00026\cdot PIAT^{2}+0.1017\cdot PIAT-7.356\\ MFE\left(Cp\right)=2.36-0.15\cdot {\left(Cp-0.65\right)}^{2}+0.23\cdot e^{-200\cdot {\left(Cp-0.1\right)}^{2}}\\ MFE\left(C_{AO}\right)=0.432\cdot C_{AO}^{2}-0.0376\cdot C_{AO}+2.2408\\ MFE\left(R_{AO}\right)=0.1956\cdot R_{AO}^{3}-0.9541\cdot R_{AO}^{2}+1.1795\cdot R_{AO}+2.0981\\ {MFE}_{M}=2.384 \end{array}\right.$$

The coefficient of nonlinear multiple correlation is R = 0.81. The significance of the correlation coefficient is confirmed by the Fisher criterion.

As a result, equations were obtained that describe the changes in the impact strength (in terms of the MFE parameter) and the internal stresses (in terms of the value of the third harmonic of the ultrasonic signal, β) of the rotational composites. A distinctive feature of the obtained equations is that they take into account not only the content of recycled polyethylene and pigment in the rotational composites but also, simultaneously, the active action of the antioxidants and their ratio (phosphite/phenolic), as well as the temperature effects of the rotational molding cycle (the process parameter PIAT).

## 4. Discussion

An analysis of the body of experimental data shows that the nonlinear acoustic response of the rotational composites is determined not by a single factor but by the superposition of at least three structural contributions: the degree of powder sintering, the level of internal stresses, and the presence of a defective structure, including porosity. This conclusion is in good agreement with the concepts of nonlinear ultrasonic spectroscopy, according to which higher harmonics arise from the anharmonicity of the intermolecular potential [50,53] and from the interaction of the wave with microstructural inhomogeneities, and are therefore substantially more sensitive to internal stresses in polyethylene than linear acoustic parameters, within the tested compositional range. In the interpretation of Cantrell [50] and in the works of Gomes [48,49,56], the third harmonic is associated primarily with the cubic nonlinearity of the medium and with the shift in the operating point of the intermolecular potential under a stressed state, while the contribution of defective boundaries and weak interphase contacts may also enhance the nonlinear response.

In this context, β is a more informative parameter for describing the internal stress of PE (Figure 4). As the rPE content of the studied composites changes, the mean value of β passes through a minimum of 0.076 rel. u. at 25% rPE and then rises to 0.124 at 75% rPE (an increase of ~63%), after which it again decreases to 0.062 at 100% rPE. Such a dependence is not a simple monotonic function and is not explained by the chemical nature of the recyclate alone. At 25% rPE, structural relaxation and stress redistribution appear to improve, as indicated by the simultaneous increase in the tensile strength (Figure 7) to 17.8 MPa (by ~4–5%), the increase in the elongation at break (Figure 9) to 308.4% (by ~5%), the increase in MFE (Figure 11) to 2.6 J/mm (by ~20–25%), and the decrease in the oxidation indices (Figure 25) in the IR spectrum to minimum values (CI = 1.35; OI = 0.31). By contrast, at 75% rPE, despite the decrease in the mean number of pores to 26.3 (by ~55–60%) and the decrease in the mean porosity to 2.4% (by ~25–30%), this produces an apparent contradiction: β reaches a maximum of 0.124 (an increase of ~63%), CI and OI rise by 17–18%, the elongation at break drops to 95.78% (by ~65–70%), the yield stress (Figure 8) decreases to 8.20 MPa (by ~15–20%), the tensile strength decreases to 16.46 MPa (by ~7–8%), and the modulus of elasticity (Figure 10) increases (by ~20–30%), indicating increased stiffness and confirming the direct correlation of β with the growth of internal stresses and the restriction of macromolecular chain mobility. This combination means that a reduction in porosity does not, in itself, lead to an improvement in properties. The correlation among MFE, ESCR, β, and the rPE content established in our earlier work [24] is shown in Figure 26.

At a high rPE content, a dense outer layer forms more rapidly owing to the increased flowability of the degraded fractions, whereas interphase incompatibilities and shrinkage stresses accumulate in the bulk of the material, manifesting in the growth of β. Meanwhile, the signal amplitude A in this region remains high, indicating that the degree of sintering is preserved and is insensitive to internal stresses (Figure 5), whereas λ increases (Figure 6) but predominantly reflects defective inhomogeneity and does not exhibit an unambiguous relation to the mechanical properties. In other words, the decrease in the visible porosity in this region of composition masks the deterioration of the internal stress state, which the third harmonic tracks.

The PIAT factor differentiates the degrees of sintering of PE more clearly. At 160 °C, the system shows the highest mean pore count (Figure 19) of 61.78, a porosity of 6.2%, a low transmitted-signal amplitude of 102.8, and a practically zero fifth harmonic at β = 0.03. In the same region, a minimum elongation at break of 92.74% and a low impact strength of 2.3 J/mm are observed. This is a typical ICS region: the particles have not yet sintered sufficiently, the structure is acoustically inhomogeneous, and strong scattering and attenuation suppress the generation of higher harmonics, especially of higher orders [48,49,50,51,52,53,54]. As PIAT increases to 200 °C, the mean porosity decreases (Figure 19) to 2.8%, and minimum oxidation indices CI and OI are observed in the IR spectrum (Figure 25). Simultaneously, A rises to 132.2 (an increase of ~29%), which indicates the formation of a denser and more uniform structure; β increases relative to the ICS region and takes moderate values, corresponding to a reduction in internal stresses and a transition to a more equilibrium state of the material; and λ becomes stably detectable, reflecting the reduction in signal scattering and the formation of a continuous medium. The impact strength reaches a maximum of 2.7 J/mm (an increase of ~17%). The mechanical properties show a similar positive trend: the elongation at break rises to 236.9% (an increase of ~155%), the yield stress to 10.7 MPa (by ~15–20%), and the tensile strength to 16.88 MPa (by ~3–5%), indicating a reduction in internal stresses and an increase in the relaxation capacity of the material; the moderate values of β are consistent with the increased ductility, and the appearance of λ with the reduction in defective inhomogeneity. A further increase in PIAT to 220 °C causes β to rise to 0.138 (an increase of more than fourfold relative to ICS) and λ to 0.20, with a simultaneous increase in porosity to 4.6% (by ~65%) and a decrease in MFE to 2.4 J/mm (by ~10–11%). This fits well with Gomes’s concept that, in the TDS zone, the third harmonic increases as an early predictor of embrittlement, since chemical changes and internal stresses alter the anharmonicity of the elastic response [51,53,54]. This is consistent with the findings of [24], which demonstrated a close correlation among β, MFE, and the ESCR parameter that, according to international standards, characterizes the internal stresses in polyethylene.

Finally, at 240 °C, composite samples of the TDS quality grade exhibit the following: the mean porosity becomes minimal—1.6% (a decrease of ~65% relative to 220 °C and of ~74% relative to ICS); the number of pores decreases to 20 (by ~68%); MFE drops to 2.1 J/mm (a decrease of ~21% relative to the optimum at 200 °C); β remains high, at 0.128; and the elongation at break is 212% (a decrease of ~10% relative to the optimum). This result has a direct practical consequence: minimum porosity does not mean the best properties, since, upon overheating, the decrease in impact strength is determined not so much by the pores as by thermo-oxidative changes and the growth of residual stresses in the composite [79]. Consequently, for diagnosing the PIAT processing window, the third harmonic appears to be a more informative marker of the transition to TDS than porosity alone.

For the pigment content of the composite (Cp), a pronounced optimum is observed in the region of 0.1–0.2%. At 0.1% Cp, the following indicators are determined: a minimum β of 0.076 and a mean porosity of 2.8% (Figure 20), a maximum MFE of 2.5 J/mm, a tensile strength of 17.08 MPa, a yield stress of 10.9 MPa, and an elongation at break of 210.8%. At 0.2% Cp, MFE remains practically at the same level (2.55 J/mm), β rises to 0.092 (by about 21%), the yield stress decreases to 8.5 MPa (by about 22%), and the porosity increases. Upon a further increase in the pigment concentration to 0.5%, β rises to 0.114 (by about 50% relative to the optimum), the porosity by about 76%, and the number of pores reaches 74. At 0% Cp, the number of pores is minimal (18), but their mean diameter is maximal (42.24 px), and MFE decreases to 2.31 J/mm (by about 9%). Thus, the region Cp = 0.1–0.2% should be considered optimal, since it is there that the best combination of minimum internal stresses, high impact strength, and an acceptable pore structure is achieved.

For the antioxidant concentration CAO, two local optima are also observed, depending on the chosen criterion. At 0.25% CAO, β decreases to 0.076 (a minimum), the elongation at break reaches 315% (an increase of about 56% compared with 0.5% CAO, and of more than 2.3-fold compared with 1% CAO), the tensile strength is 17 MPa, and the yield stress is 10 MPa. This indicates a minimum level of internal stresses and the maximum ductility of the material. By contrast, at 0.5% CAO, the impact strength remains high (2.5 J/mm), the amplitude rises to 129.6, and the modulus of elasticity reaches 304.6 MPa—the maximum value among the investigated CAO concentrations. In this case, β rises to 0.112 (by about 47% relative to 0.25% CAO), and λ increases to 0.018. At 1% CAO, MFE reaches its absolute maximum of 2.7 J/mm (about 8% higher than at 0.5% CAO); however, β also becomes maximal (0.118), which indicates a higher level of internal stresses. At 0.75% CAO, a deterioration of the structure is observed: the number of pores rises to 61.9, the porosity (Figure 21) to 4.3%, and MFE decreases to 2.3 J/mm. Consequently, if the main criterion for the composite is optimal ductility at minimal internal stresses, the region CAO = 0.25% is preferable; if priority is given to the main parameter determining the service life of tanks mounted on agricultural machinery (tractors, sprayers, seeding complexes, etc.)—the impact strength—the region 0.5–1.0% CAO is acceptable, but at the cost of an increase in β and a deterioration of the stress state of the composites.

The ratio of the phenolic and phosphite antioxidants, RAO, also exhibits a pronounced working optimal interval. At RAO = 0.5, the parameter β is minimal (0.072), λ is also low (0.06), the yield stress is 10 MPa, and the elongation at break is 214%, which corresponds to the most equilibrium structural state. In this case, the phosphite antioxidant predominates in the system, promoting a more effective relaxation of internal stresses. At RAO = 1.0, MFE reaches a maximum of 2.5 J/mm (about 15% higher than at RAO = 2), and the mean porosity decreases (Figure 22 and Figure 27) to 2.9%; however, β rises to 0.102. At an equal ratio, a balance is realized between the phenolic and phosphite components that is favorable for the impact strength. At RAO = 2, the number of pores reaches 68, the porosity reaches 4.8%, and MFE decreases to 2.2 J/mm, which is associated with the predominance of the phenolic antioxidant and the deterioration of structural uniformity. Thus, the interval from 0.5 to 1.0 should be considered optimal. The value 0.5 is preferable in terms of the level of internal stresses and acoustic uniformity, whereas RAO = 1 is a local optimum in terms of impact strength and porosity. The relationship between the mean porosity and β under the influence of rPE, CAO, and RAO is shown in Figure 27.

In accordance with the experimental results, λ reflects the nonlinearity of the ultrasonic signal associated with defective boundaries and interphase contacts, and therefore characterizes not the total number of pores but the degree of structural inhomogeneity of the material. At PIAT = 160 °C, despite the maximum porosity, λ is practically zero owing to the strong attenuation of the ultrasound in the unformed structure. Upon the transition to PIAT = 200–240 °C, λ rises to 0.14–0.20 against a background of decreasing porosity down to 2.8%, which indicates the formation of a continuous medium and the appearance of stable nonlinear contacts. Similarly, at 75% rPE, λ remains high (0.12) at low porosity (2.4%), whereas at 100% rPE, λ decreases to 0.02 against a background of structural deterioration. Thus, λ characterizes the degree of development of the defective structure and is sensitive to the presence of interphase inhomogeneities in the already-formed material.

A comparison of the values of λ and A with the porosity and mechanical-property parameters reveals their different physical sensitivities. The fifth harmonic λ does not show a correlation with the total porosity but increases upon the transition from ICS to NS (0 → 0.14–0.20), which coincides with a decrease in porosity of ~55–75% and an increase in MFE of ~17%. However, upon the transition to TDS (PIAT 220–240 °C), λ remains at an elevated level (up to 0.20), despite the decrease in MFE of ~10–21%, which indicates its correlation not with porosity but with the development of defective inhomogeneity in the already-formed structure, in agreement with the findings of [56]. Similarly, for the rPE factor, as the porosity increases from 2.4% to 4.3%, λ decreases from 0.12 to 0.02, which confirms the absence of a correlation between λ and the number of pores.

The amplitude A, by contrast, exhibits a stable correlation with the degree of sintering and the acoustic conductivity of the material. Upon the transition from PIAT 160 °C to 200 °C, A rises from 102.8 to 132.2 (by ~29%), accompanied by a decrease in porosity of ~55% and an increase in MFE of ~17%. A appears to correlate with the microbubble content of the material. However, upon a further increase in PIAT to 240 °C, the amplitude remains high (132.4–133.4) despite the decrease in MFE to 2.1 J/mm (by ~21%), which indicates the absence of a correlation between A and the mechanical properties in the TDS region.

The parameter β exhibits a direct correlation with the modulus of elasticity and an inverse correlation with the elongation at break; this dependence is most pronounced at 75% rPE, where β reaches its maximum. In this region, the material simultaneously shows increased stiffness and minimal deformability, indicating a critical restriction of macromolecular chain mobility. Thus, it is precisely at 75% rPE that a state is realized in which the growth of β corresponds directly to the maximum accumulation of internal stresses, manifested in the combination of a high modulus and a sharp drop in ductility.

It should be noted that although the relationship between β and the internal stress state of the composites is correlational in nature, several lines of evidence support its use as a diagnostic indicator within the tested compositional range. First, the PDP models yield statistically significant correlation coefficients (R = 0.76 for β, R = 0.81 for MFE; Fisher-criterion-validated), confirming the reproducibility of the observed trends. Second, in the earlier work [24], a cross-correlation among β, MFE, and the ESCR parameter—which, according to ASTM D1693, directly characterizes internal stresses in polyethylene—was demonstrated (Figure 26), providing independent mechanical validation. Third, the trends in β are fully consistent with the independent IR-spectral (CI, OI), TGA, and porosity data: whenever β increases, the oxidation indices and porosity change in the predicted direction, while the impact strength and ductility decrease. Moreover, accelerated UV-aging experiments (333 and 666 h) confirmed that β continues to track internal stresses under photo-oxidative degradation, correlating with changes in CI, OI, and MFE over time. Fourth, the theoretical framework of Cantrell [50] and Gomes [48,49,56] provides a physical mechanism linking the third harmonic to the cubic nonlinearity of the intermolecular potential under a stressed state, supporting the interpretation that β reflects genuine material changes rather than measurement artifacts. Nevertheless, a fully quantitative calibration of β as an absolute stress indicator would require independent stress measurements (e.g., X-ray diffraction or hole-drilling), which remains a direction for future work.

The optimal formulation is therefore the following combination: 25% rPE; PIAT ≈ 200 °C; Cp ≈ 0.1–0.2%; CAO ≈ 0.25–0.5%; RAO = 0.5–1.0. In this region, the parameter β is at or close to its minimum level (0.076–0.09), MFE reaches 2.6 J/mm, and the porosity is 2.5–3%, which corresponds to the best combination of mechanical properties and structural uniformity. In this region, the elongation at break reaches 230–300%, the yield stress is 10–11 MPa, and the tensile strength is 16.8–17.8 MPa, which indicates high ductility and adequate strength of the material at moderate stiffness. This combination is realized in a well-formed structure (high amplitude A ≈ 130) and a low level of internal stresses (moderate values of β and λ), corresponding to the NS state.

The contact angle (θ°) measurements of the composites (Figure 12 and Figure 13) showed that the addition of recycled polyethylene (rPE) does not have a technically significant effect on the hydrophilicity of the surface. Thus, in the composition with 25% rPE, θ decreased by only 5° relative to vPE. The introduction of antioxidants (CAO) in the range of 0.25–1.00% and the variation in RAO within 0.33–3.00 likewise did not lead to a statistically significant change in the hydrophobicity of the composites. Consequently, the combination of rPE, CAO, and RAO in the studied ranges has no substantial effect on the hydrophobicity of the PE-composite surface, and the introduction of these components should not adversely affect the performance characteristics of the products.

The thermogravimetric data (Figure 16 and Figure 17) show how the rPE, CAO and RAO contents affect the thermal stability of the composites. The lowest mass loss was observed at 0% rPE (M = 90.4%), reflecting the highest thermal stability of the composite without rPE addition. As the rPE share increased to 100%, the mass loss reached 96.26%, demonstrating a slight decrease in thermal resistance at high recycled-polyethylene concentrations. The variation in CAO and RAO had a moderate effect on M. The antioxidant concentration adjusts the thermal stability of the composite, but no clearly pronounced stabilizing effect is observed. RAO does not affect M and does not fully compensate for the decrease in thermal stability at high rPE shares. The onset temperature of thermal degradation (T_b_, °C) also shows a dependence on the rPE content and on the parameters of the antioxidant system. At 25% rPE, T_b_ decreased to 375.5 °C relative to pure vPE (Tb = 405.9 °C), indicating a slight decrease in the thermal resistance of the composite. Overall, the influence of CAO and RAO on T_b_ can be considered moderate, since the total variation does not exceed ±6% of the minimum value and does not lead to a significant deterioration of the stability of the composites. At 25% rPE, T_max_ decreased to 470.0 °C relative to pure vPE (T_max_ = 487.3 °C), reflecting a slight decrease in thermal resistance. The influence of CAO and RAO on T_max_ can be characterized as moderate, since the temperature fluctuations do not exceed ±6% of the minimum value and do not cause a noticeable change in the thermal resistance of the composites. The thermogravimetric analysis showed that the introduction of rPE does not lead to a technically significant decrease in the thermal stability of the composites, whereas the variation in CAO and RAO has only a moderate effect on T_b_ and T_max_.

After UV aging for 333 and 666 h in the artificial-weathering chamber, corresponding to the conditional 5- and 10-year service horizons defined in Section 2.8, two processes take place in the composite: photo-oxidative degradation (scission of C–C bonds) and a reduction in the content of amorphous regions caused by the shortening of chain lengths—that is, an increase in crystallinity (restriction of chain mobility and local densification). As a result, the MFE and the third harmonic β change in opposite directions, since they reflect different processes. In accordance with the ultrasonic analysis methodology, the parameter β is sensitive to the anharmonicity of the intermolecular potential, the bond stiffness, and the level of internal stresses in the polymer. MFE, by contrast, reflects the ability of the material to absorb impact energy and is determined by the mobility of the macromolecules and the content of the amorphous fraction. The IR-spectral parameter CRYST reflects the degree of crystallinity and is directly related to the strength of the composite.

In general, upon the transition from the initial state to composite samples aged to the conditional 10-year horizon (666 h), an increase in β (Figure 23) and a simultaneous decrease in or stabilization of MFE (Figure 24) are observed. This is confirmed by the data of Section 3.10, which noted an increase in β as well as a rise in the IR indices (Figure 25) CI and OI, with a simultaneous decrease in impact strength and a change in crystallinity after UV exposure. Thus, the increase in β at the conditional 10-year horizon of Northern Kazakhstan reflects the accumulation of internal stresses and the increase in structural stiffness as a result of oxidative processes, whereas the decrease in MFE is associated with a partial loss of the ability of the chains to deform plastically. In this case, the change in CRYST (Figure 25) plays a fundamental role: the preservation or growth of crystallinity indicates the formation of a stronger and more stable structure.

An analysis of the influence of the rPE content shows that the optimal region after UV aging does not lie at the maximum recycled-polyethylene content. At 25% rPE, the MFE value at the conditional 10-year horizon (666 h) is 2.47 J/mm, practically at the level of the initial material (2.49 J/mm), whereas at 50% rPE it decreases to 2.07 J/mm. At the same horizon, β increases to 0.208 at 25% rPE and is 0.194 at 50% rPE, which indicates a growth of internal stresses. The IR data confirm the intensification of oxidation: CI increases to 1.63 at 25% and to 1.75 at 50% rPE. Meanwhile, the crystallinity increases with increasing rPE and reaches values of about 0.82 at 100% rPE, indicating an increase in strength and a decrease in the impact strength of the composite. However, MFE decreases, indicating excessive stiffness and a loss of impact strength. Thus, an increase in the share of recycled polyethylene intensifies the oxidative degradation and reduces the impact strength, despite the increase in strength according to CRYST; therefore, the optimal rPE content is determined by a compromise between these parameters.

The influence of the pigment content on the properties of the composite under UV exposure (666 h, conditional 10-year horizon) shows that the optimal Cp shifts toward ≈0.5%. At a low pigment content (0–0.1%), a relatively high MFE is retained (2.35–2.36 J/mm), but the UV stabilization is insufficient. At Cp = 0.5%, MFE remains at the level of 2.32 J/mm, while β at the conditional 10-year horizon decreases to 0.19 compared with the maxima at Cp = 0.1–0.2% (up to 0.20–0.21). Upon a further increase in Cp to 1%, a deterioration of MFE (to 2.13 J/mm) and a rise in OI to 0.54 are observed, indicating an excessive pigment concentration and a deterioration of the microstructure of the composite. CRYST, however, reaches maximum values (up to ~0.85–0.87), indicating an increase in strength; however, this is already accompanied by a loss of impact strength (owing to the reduction in the number of amorphous regions). Thus, Cp = 0.5% provides the best balance among strength, UV resistance, and the impact strength of the composites (in terms of the MFE parameter).

The antioxidant concentration has a key influence on the studied aging processes of the composites during the outdoor service of the products under the open sky of Northern Kazakhstan (the action of UV radiation). At CAO = 0.5%, the maximum MFE value after UV aging is achieved (2.39 J/mm), at β (666 h) ≈ 0.20. Upon an increase in CAO to 0.75%, a decrease in β to 0.17 is observed, indicating a reduction in internal stresses; however, MFE simultaneously decreases to 2.22 J/mm. The IR indices (OI ≈ 0.38–0.39, moderate CI) show that effective suppression of oxidation is ensured in this range. CRYST is retained at a high level (~0.80–0.82), which indicates the preservation of structural strength. Thus, the range CAO = 0.5–0.75% provides the optimal balance among the strength, stability, and impact strength of the composites under the service conditions of the products—tanks for the storage of pesticides, road blocks, etc.—under the open sky.

The antioxidant ratio (RAO) also has a substantial influence on the stability of the composites under UV radiation. At RAO = 1.0, the best combination of properties is achieved: MFE (UV, 666 h) = 2.37 J/mm and β (UV, 666 h) ≈ 0.17. Upon deviation from this ratio (RAO = 2–3), MFE decreases to 2.22–2.07 J/mm, despite the presence of stabilizers. This indicates a disruption of the synergistic effect of the phenolic and phosphite antioxidants. In addition, at RAO = 1.0, CRYST reaches high values (~0.82), which additionally confirms the formation of a strong and stable structure after UV aging.

The increase in impact strength observed after 666 h of UV aging at CAO = 0.25% and RAO = 2 is most plausibly attributed to the combined action of three concurrent effects: the UV-screening effect of carbon black, the radical-scavenging effect of the antioxidant package, and a structural rearrangement of the polyethylene matrix. Over the exposure interval studied, photodegradation at CAO = 0.25% and RAO = 2 is accompanied by a decrease in the crystallinity of the composites, as evidenced by the reduction in the crystallinity index CRYST (Figure 25, IIIb and IIIc). The corresponding increase in the amorphous fraction promotes a more ductile fracture mechanism. Since UV exposure of rotationally molded articles predominantly affects the surface layer, while the bulk of the wall thickness remains essentially unaltered, the net effect of ultraviolet radiation, carbon black, and the antioxidants and their ratio is an increase, rather than a decrease, in impact strength—in agreement with [80,81].

Thus, for products operating under direct solar radiation over the conditional 10-year horizon considered here (tanks for the storage of pesticides, road blocks, etc.), the optimal composite composition is determined not by the maximization of a single parameter but by a balance among them: a high level of impact strength (in terms of the MFE parameter), a moderate value of β as an indicator of internal stresses, and a high level of CRYST as an indicator of strength, at a controlled level of thermal degradation (the IR markers CI and OI). On the basis of the overall analysis, it was established that, for the outdoor service of products (tanks for the storage of liquid mineral fertilizers), the following composition is optimal: an rPE content of 25–50% (preferably 25%), Cp ≈ 0.5%, CAO = 0.5–0.75%, and RAO = 1.0. Such a composition provides a combination of high strength (owing to the increased crystallinity), resistance to UV degradation [82], and the preservation of impact strength under prolonged exposure to solar radiation.

At the request of AVAGRO LLP—the partner organization of this study—a nomogram was developed for the proposed formulation (for service without UV radiation—underground septic tanks), presented in Figure 28.

The nomogram in Figure 28 gives, without calculation, the theoretical height of the third harmonic (β) for specific formulations, taking into account their contents of rPE, pigment, and antioxidants, as well as the antioxidant ratio (RAO) in the composite. The nomogram also solves the inverse problem—that is, to select a formulation in terms of the contents of rPE, pigment, antioxidants, and their ratio (RAO) in the composite as a function of the internal stresses (in terms of the height of the third harmonic (β)) for a specific product according to the customer’s technical specification. Once refined in production, the models derived here can support rapid quality control of finished articles.

The revealed correlation between the height of the third harmonic (β) of the ultrasonic baseband signal and the antioxidant content during atmospheric and UV aging of the composites warrants further study by X-ray diffraction (XRD) and scanning electron microscopy (SEM) in order to identify the causes of this effect at the level of the microstructure of the plastics.

## 5. Conclusions

(1) The influence of the contents of pigment (Cp) and antioxidants (CAO), as well as their ratio (phenolic/phosphite) (RAO), on the properties of rotational composites containing recycled PE—without accounting for solar/UV radiation, with potential application in the manufacture of underground septic tanks, containers, etc.—was studied. It was found that the influence of the antioxidants has a complex character, and two local optima were revealed (the first at CAO = 0.25% and the second at 0.5%). At the first optimum (CAO = 0.25%), the minimum of internal stresses (β = 0.076) and the maximum ductility of the composite are achieved. At the second optimum (CAO = 0.5%), the maximum of the parameter most important for the service of tanks—the impact strength (in terms of MFEsp.)—is observed, but at the cost of an increase in β and a deterioration of the structural state. For the antioxidant ratio (phenolic/phosphite), the optimal range is RAO = 0.5–1.0. The value RAO = 0.5 is preferable by the criterion of minimum internal stresses, whereas RAO = 1.0 provides the best impact strength. At RAO above 1.0, the internal stresses grow and the impact strength decreases. Therefore, for the manufacture of underground septic tanks and containers, on the basis of the analysis of the impact strength (MFEsp.), the strength (tensile strength, yield stress, etc.), and the thermal (Tb, Tmax, °C), acoustic (height of the third harmonic), and surface (porosity, IR spectroscopy, and θ°) properties of the composites, the optimal component content is as follows: rPE = 25%, PIAT = 200 °C, Cp = 0.1–0.2%, CAO = 0.25–0.5% (for products without UV exposure, representing a compromise between minimum internal stresses at 0.25% and maximum impact strength at 0.5%; for UV-exposed products; see point 2), and RAO = 0.5–1.0. In this region, a minimum level of internal stresses, a high MFE = 2.6 J/mm, a porosity of 2.5–3%, and high ductility of the material are achieved, consequently leading to the maximum possible service life of the products.
(2) A series of studies was carried out on the influence of UV radiation (333 and 666 h in the artificial-weathering chamber), interpreted as conditional 5- and 10-year service horizons for Northern Kazakhstan within the acceleration model of Section 2.8, on the impact strength (MFEsp.), the height of the third harmonic, and the IR surface markers of the composites. On the basis of the analysis of the data obtained, for products operating under solar radiation (i.e., tanks for process water, road barriers, etc.), the optimal formulation is rPE = 25%, Cp = 0.5%, CAO = 0.5–0.75%, and RAO = 1.0. Such a composition provides a combination of high strength (owing to the increased crystallinity), resistance to UV degradation, and the preservation of consistently high impact strength under prolonged exposure to solar radiation. The introduction of more than 25% rPE into the composite leads, under UV aging, led to a significant decrease in the impact strength of the composites.
(3) The introduction of rPE, the antioxidants, and their ratio has no technically significant effect on the thermal properties of the composites and practically does not change the hydrophobicity of the surface. Therefore, the recycling of up to 50% recycled PE in the studied rotational composites will yield engineering products with adequate thermal resistance. The service horizons quoted throughout are conditional: they follow from the acceleration model of Section 2.8 and are intended for the relative ranking of the formulations, not as a prediction of the calendar service life of a specific article.
(4) At the request of the partner organization AVAGRO LLP, a nomogram CAO = f(β, rPE) was developed for the formulation, without accounting for the action of UV radiation. The nomogram allows a composition to be selected quickly to the specified requirements for the level of internal stresses, without resorting to complex, cumbersome calculations.

## Figures and Tables

**Figure 1:**
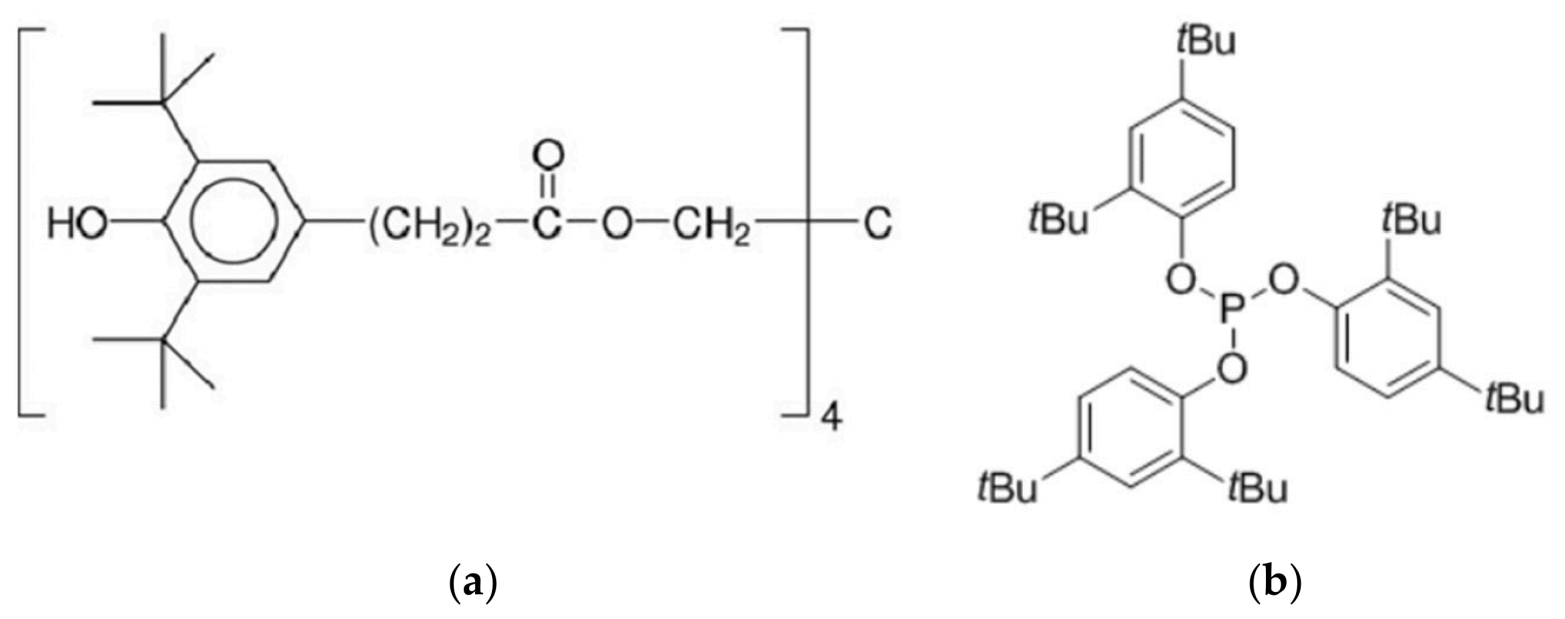
Structural formulae of the antioxidants used: (**a**) phenolic antioxidant 1010; (**b**) phosphite antioxidant 168.

**Figure 2:**
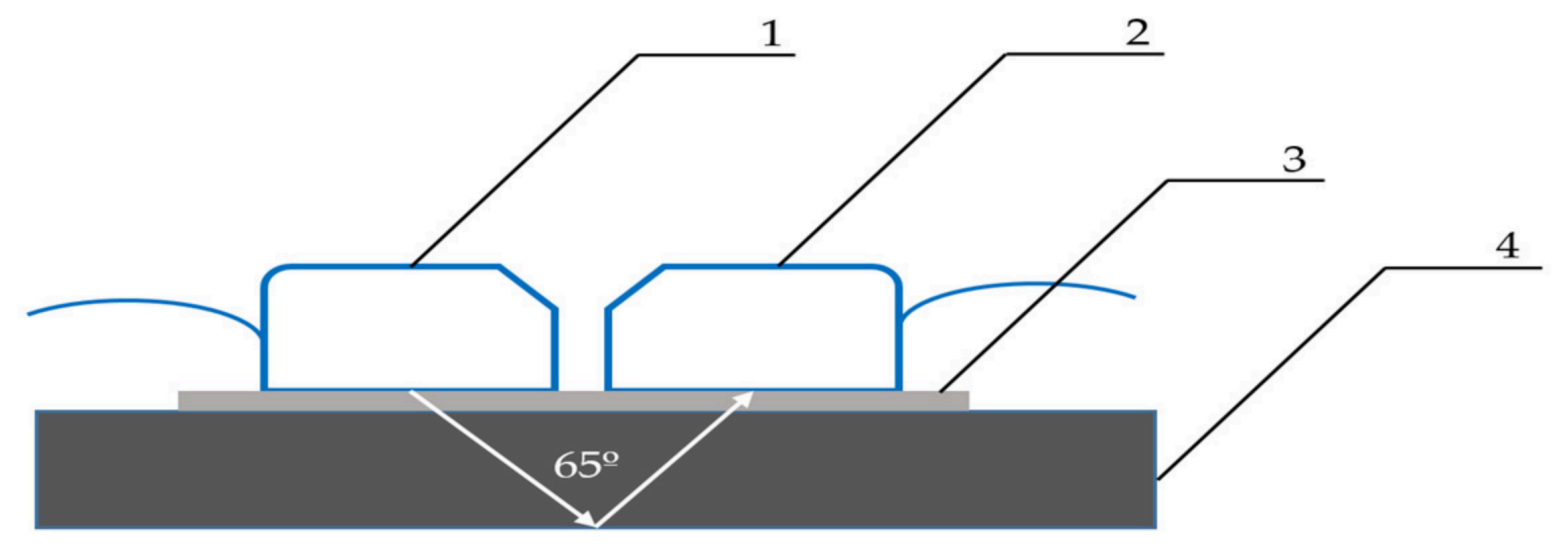
Mirror-shadow flaw-detection scheme: 1—transmitting inclined piezoelectric sensor; 2—receiving inclined piezoelectric sensor; 3—glycerin layer; 4—plastic sample, 2 mm thick.

**Figure 3:**
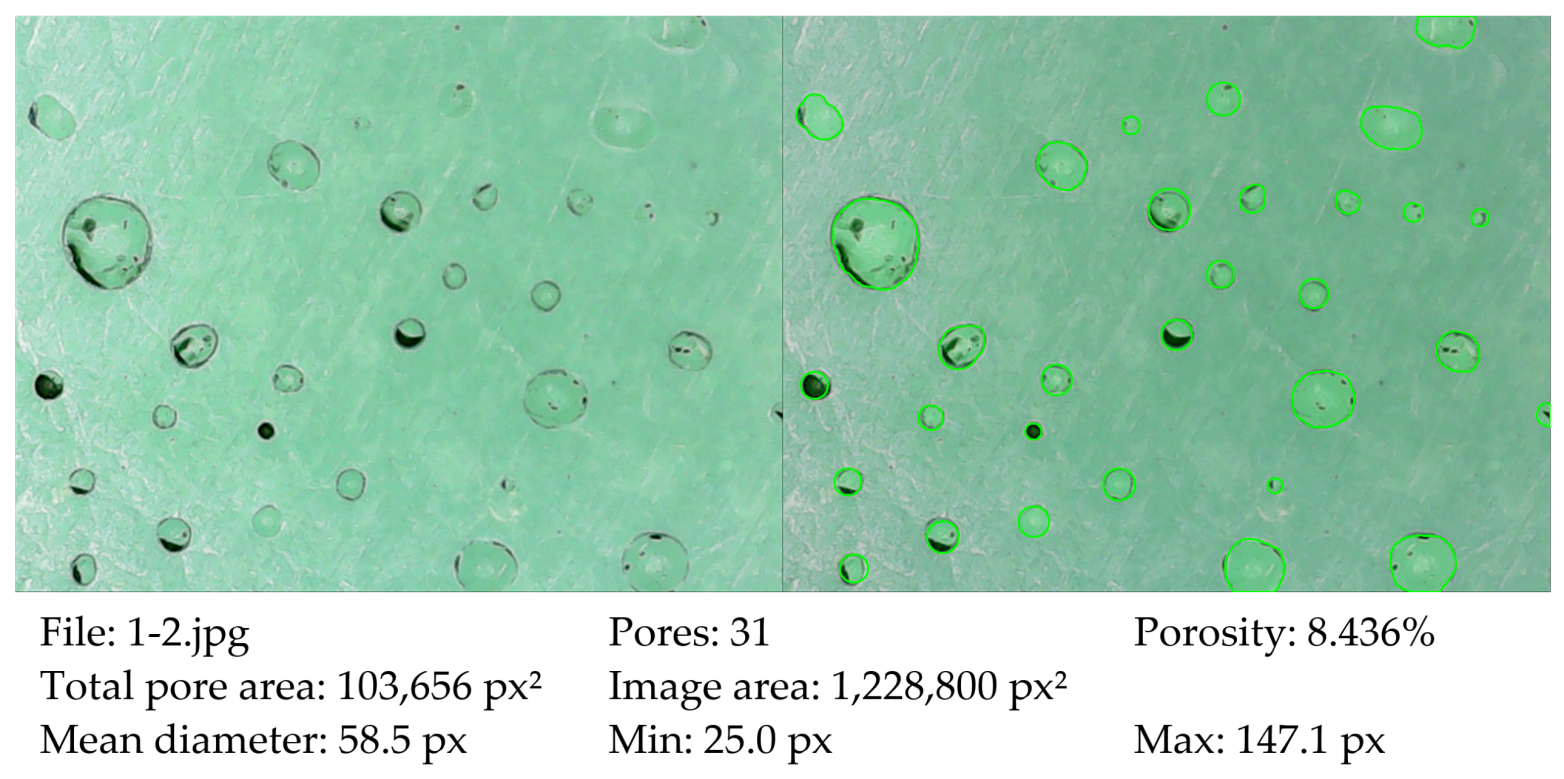
Photo of the Pore Counter program in action.

**Figure 4:**
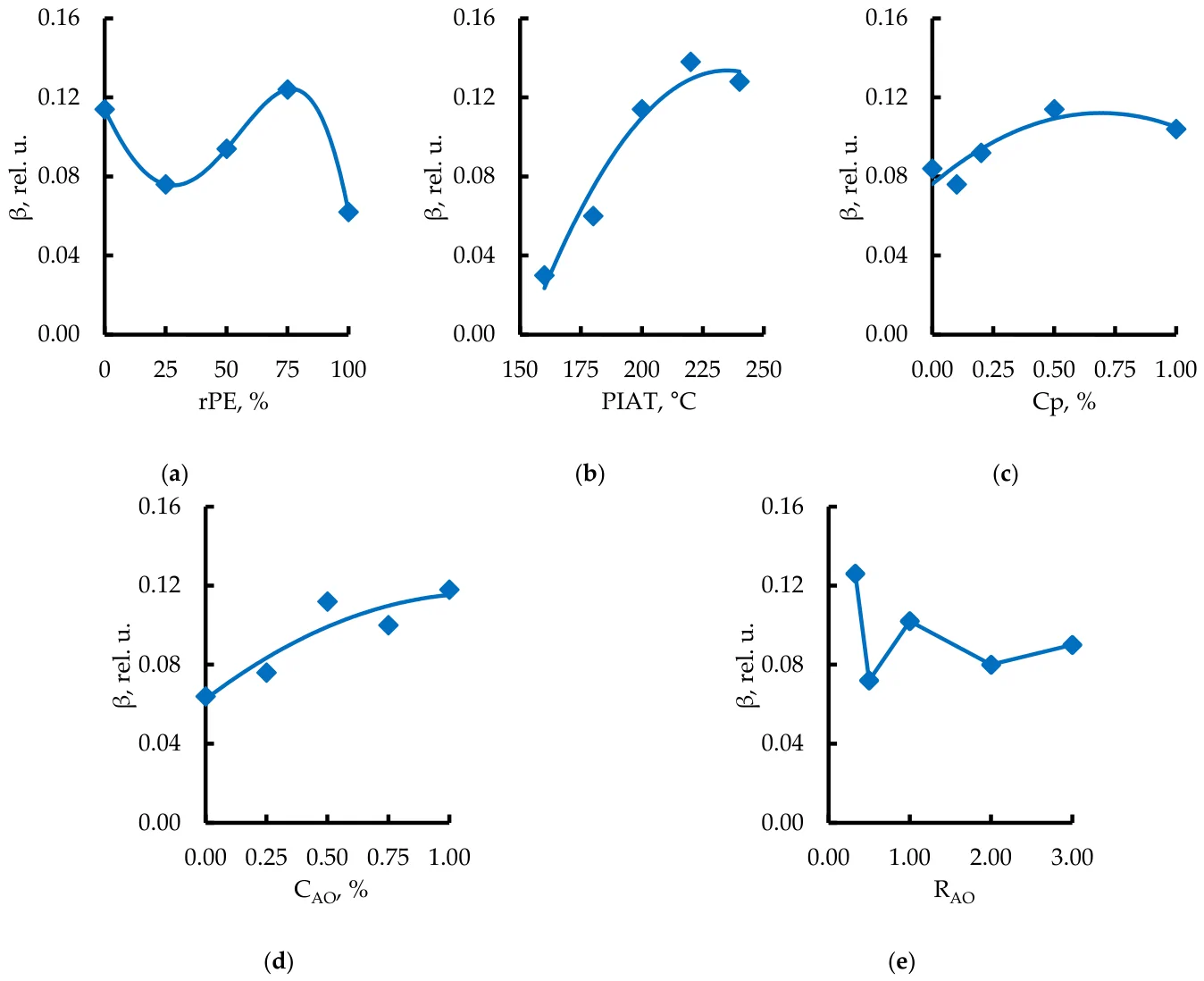
The influence of rPE (**a**), PIAT (**b**), Cp (**c**), CAO (**d**), and RAO (**e**) on β.

**Figure 5:**
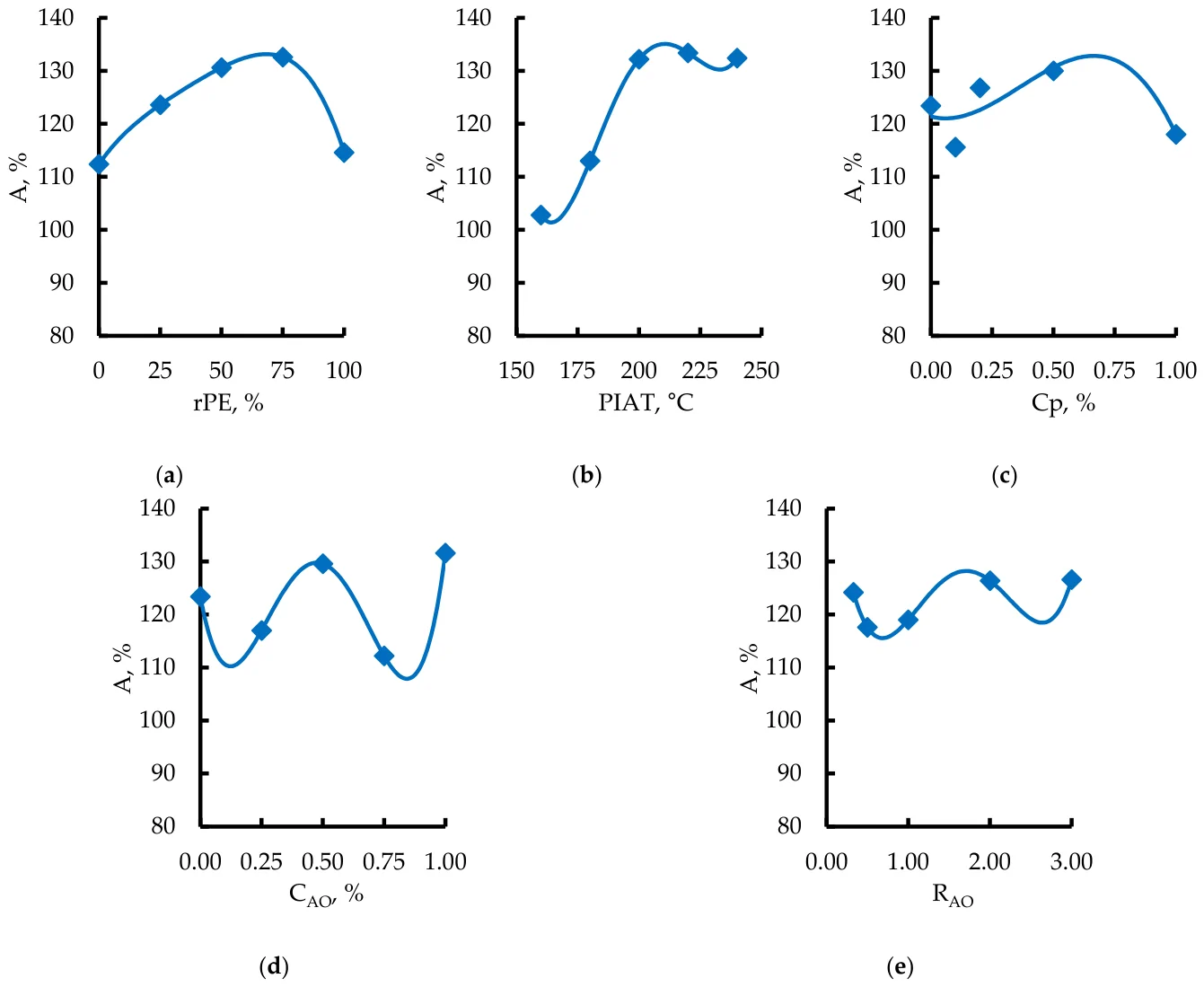
The influence of rPE (**a**), PIAT (**b**), Cp (**c**), CAO (**d**), and RAO (**e**) on A.

**Figure 6:**
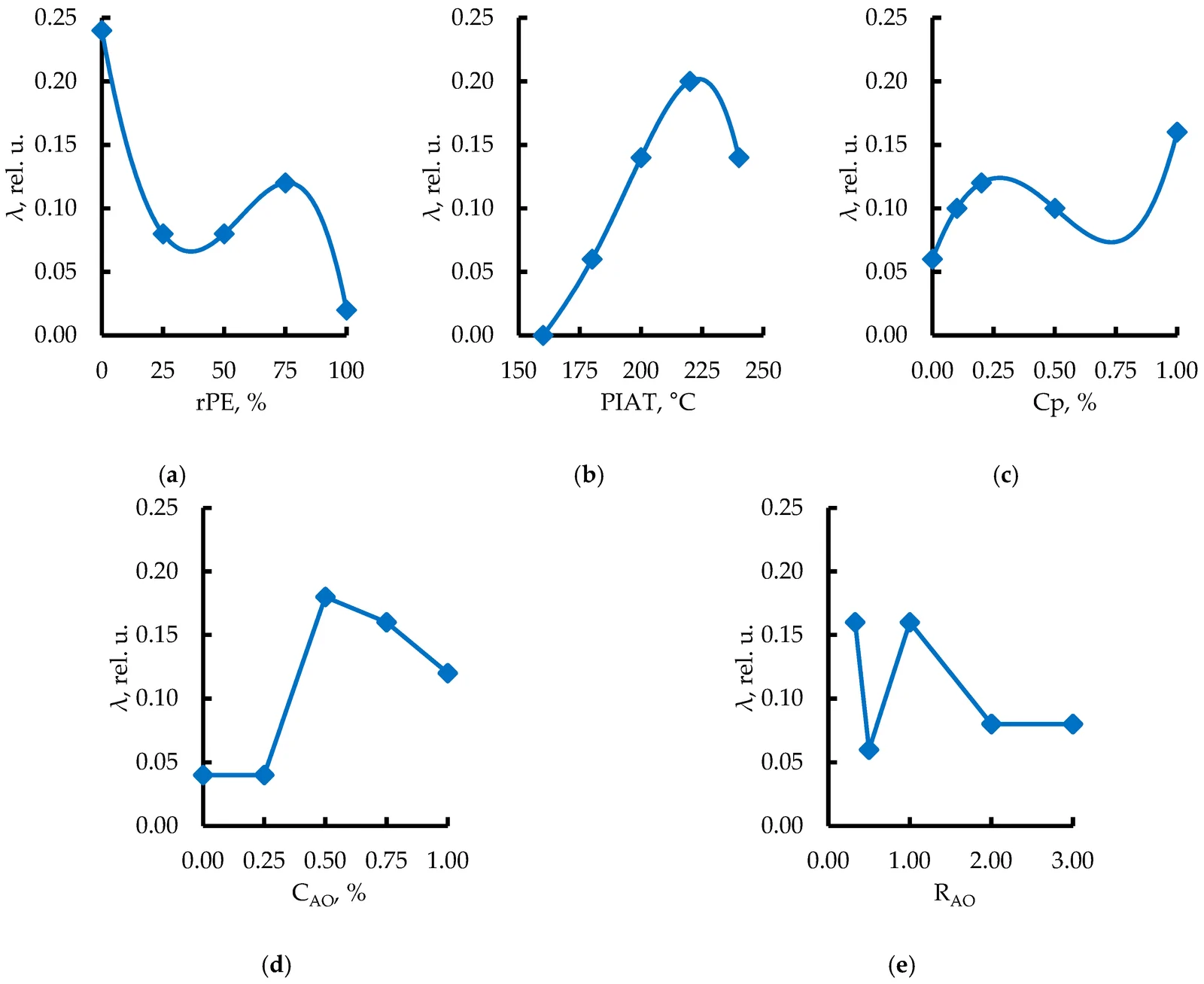
The influence of rPE (**a**), PIAT (**b**), Cp (**c**), CAO (**d**), and RAO (**e**) on λ.

**Figure 7:**
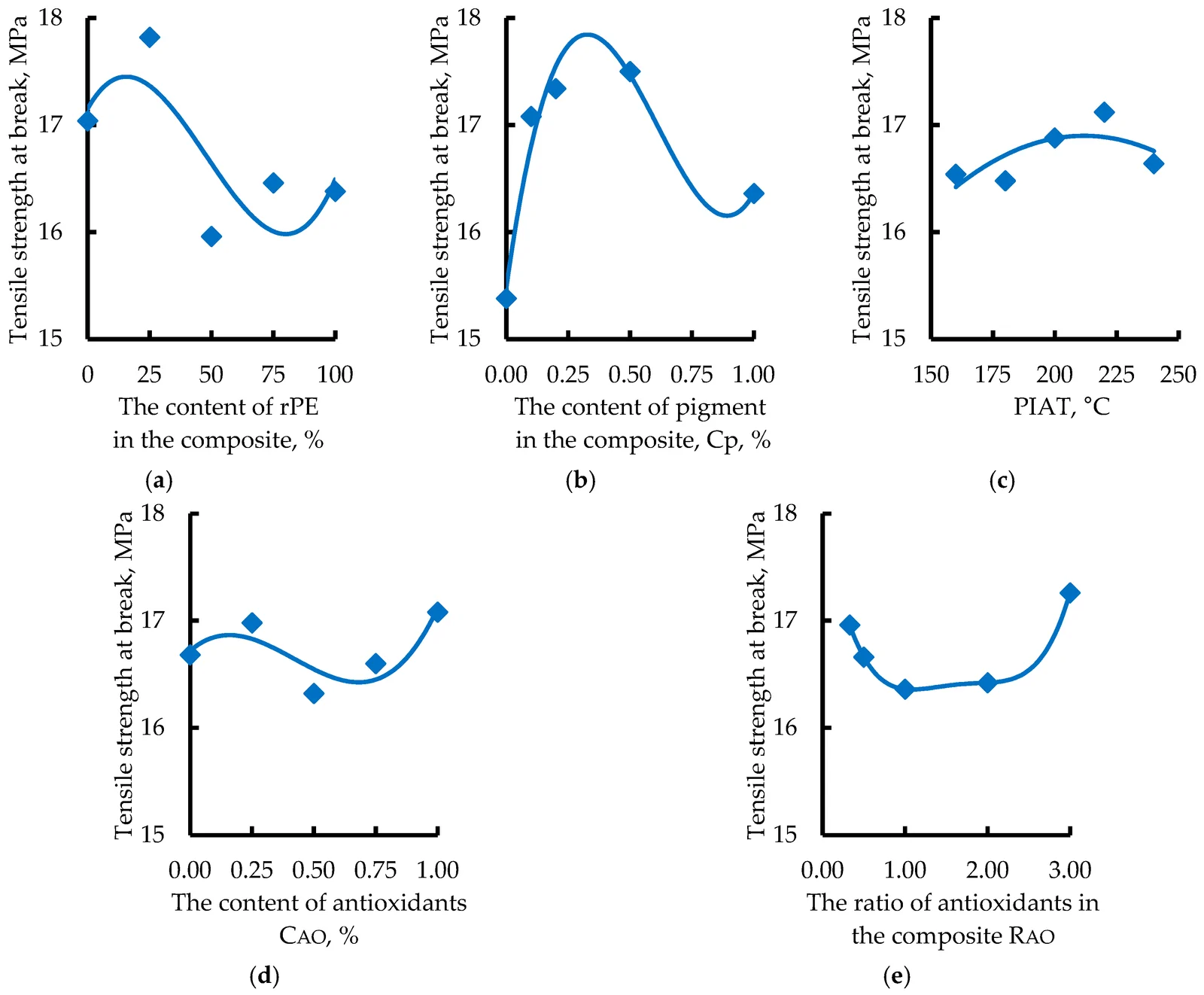
Effects of (**a**) rPE content, (**b**) Cp, (**c**) PIAT, (**d**) CAO, and (**e**) RAO on the tensile strength at break.

**Figure 8:**
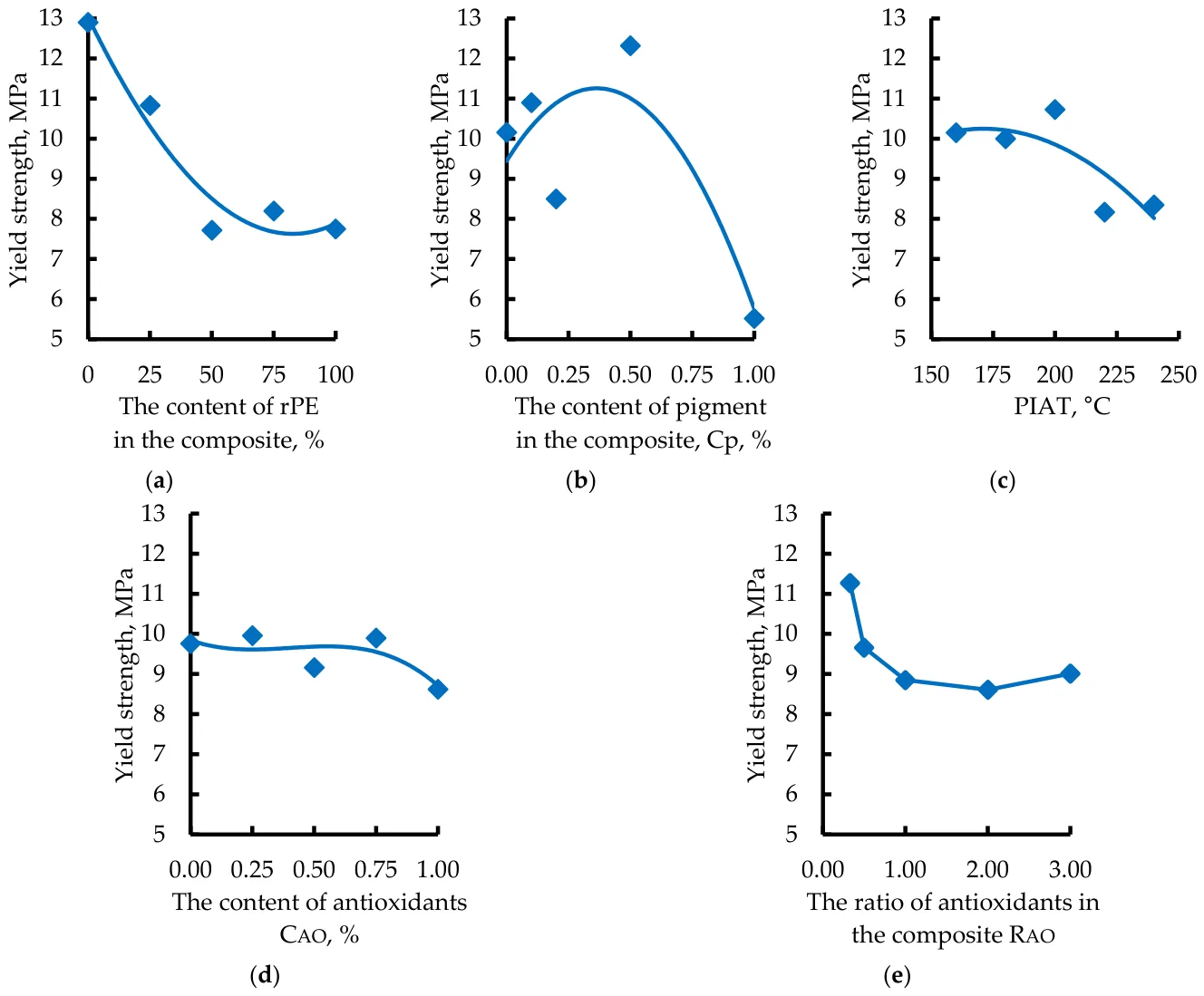
Effects of (**a**) rPE content, (**b**) Cp, (**c**) PIAT, (**d**) CAO, and (**e**) RAO on the yield strength.

**Figure 9:**
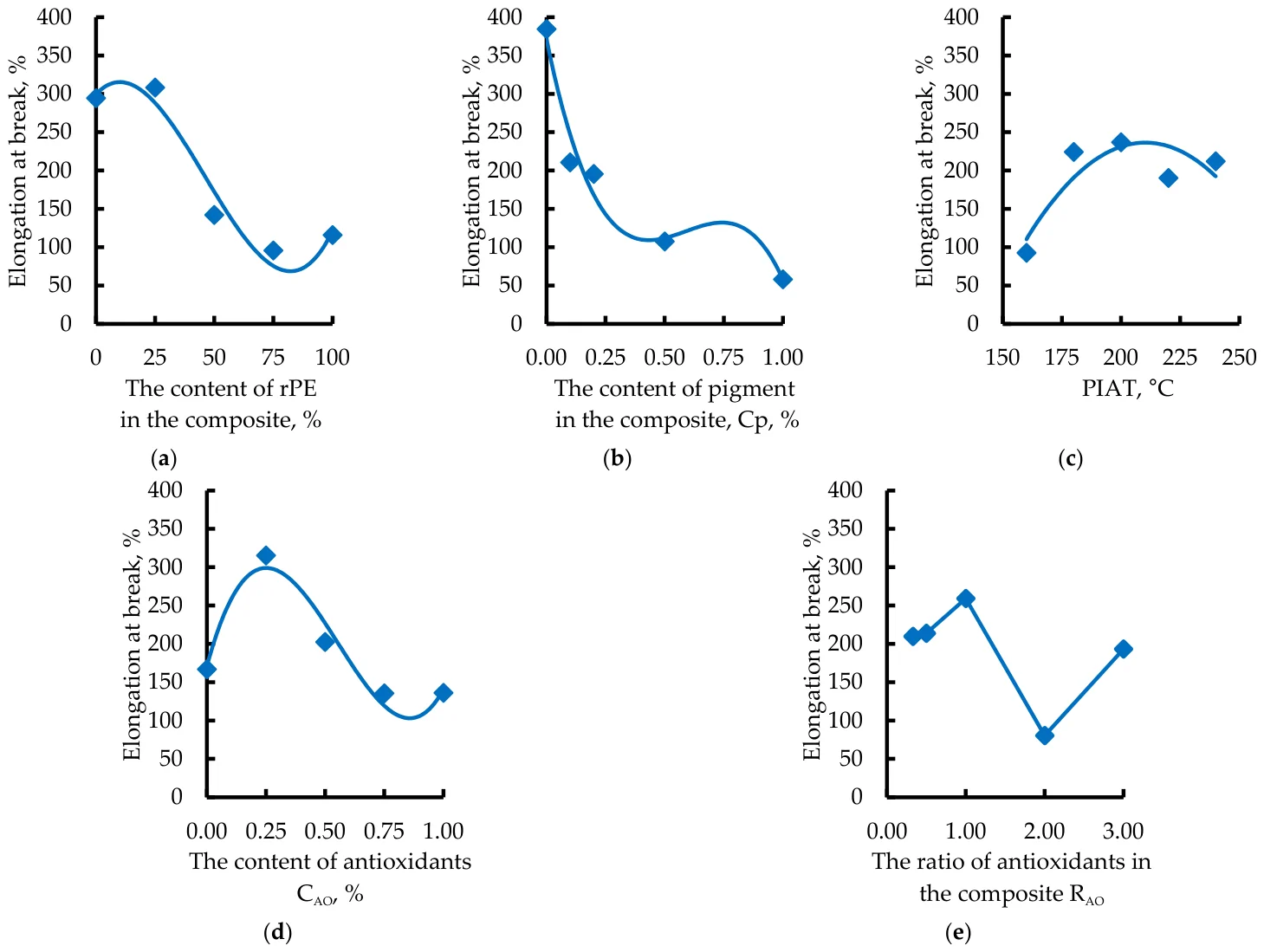
Effects of (**a**) rPE content, (**b**) Cp, (**c**) PIAT, (**d**) CAO, and (**e**) RAO on the elongation at break.

**Figure 10:**
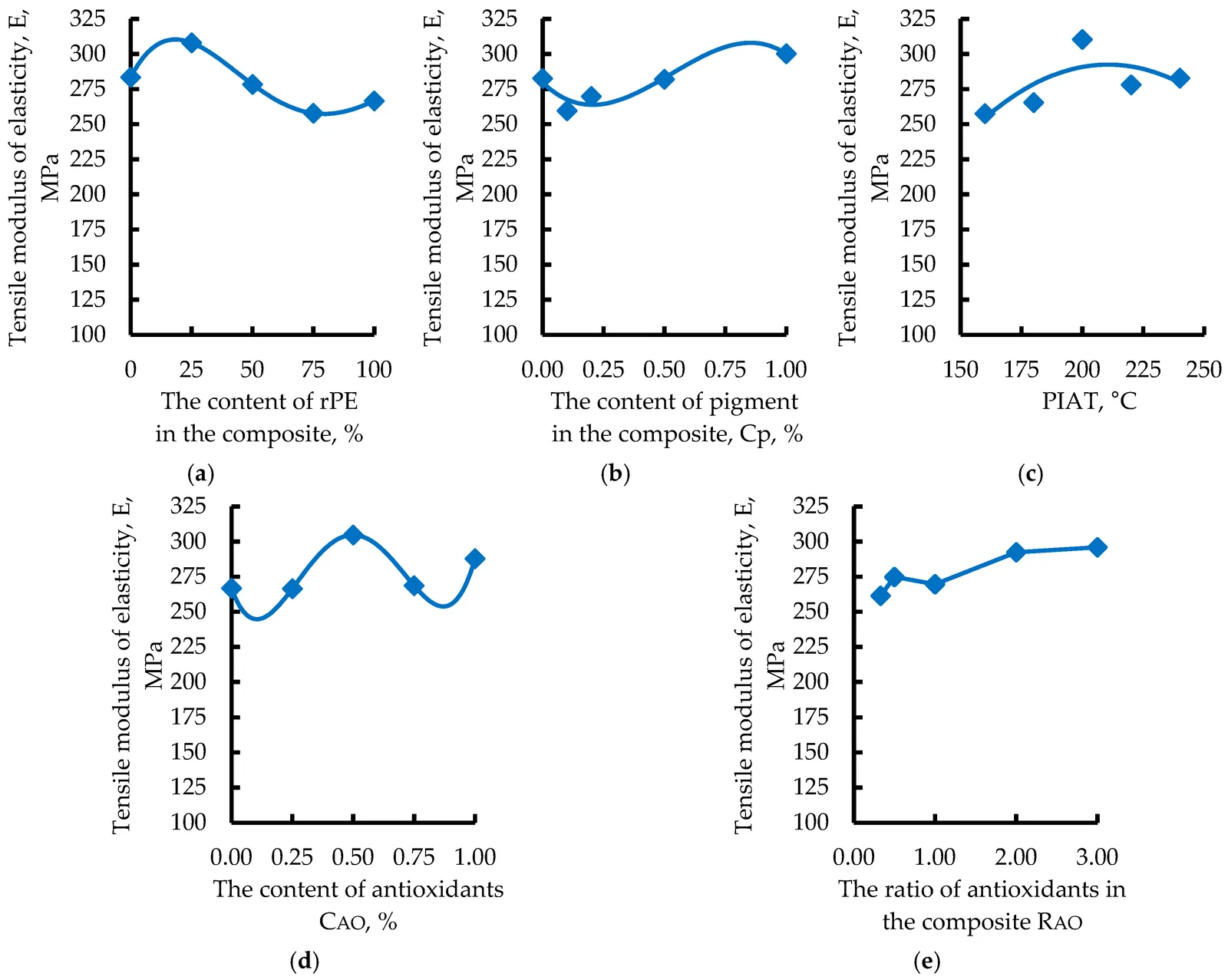
Effects of (**a**) rPE content, (**b**) Cp, (**c**) PIAT, (**d**) CAO, and (**e**) RAO on the tensile modulus of elasticity, E (MPa).

**Figure 11:**
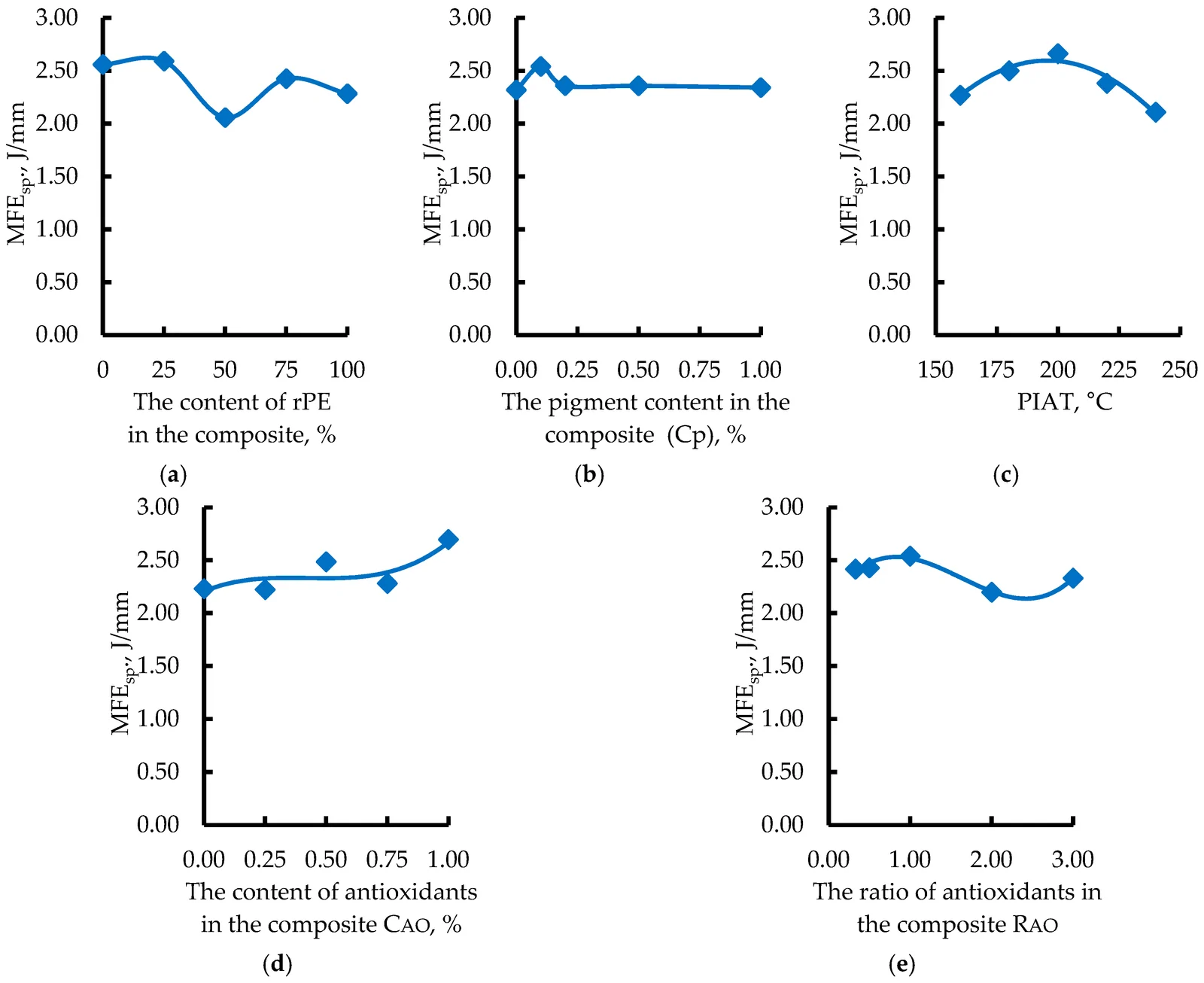
Effects of (**a**) rPE content, (**b**) Cp, (**c**) PIAT, (**d**) CAO, and (**e**) RAO on the specific mean failure energy (MFEsp., J/mm).

**Figure 12:**
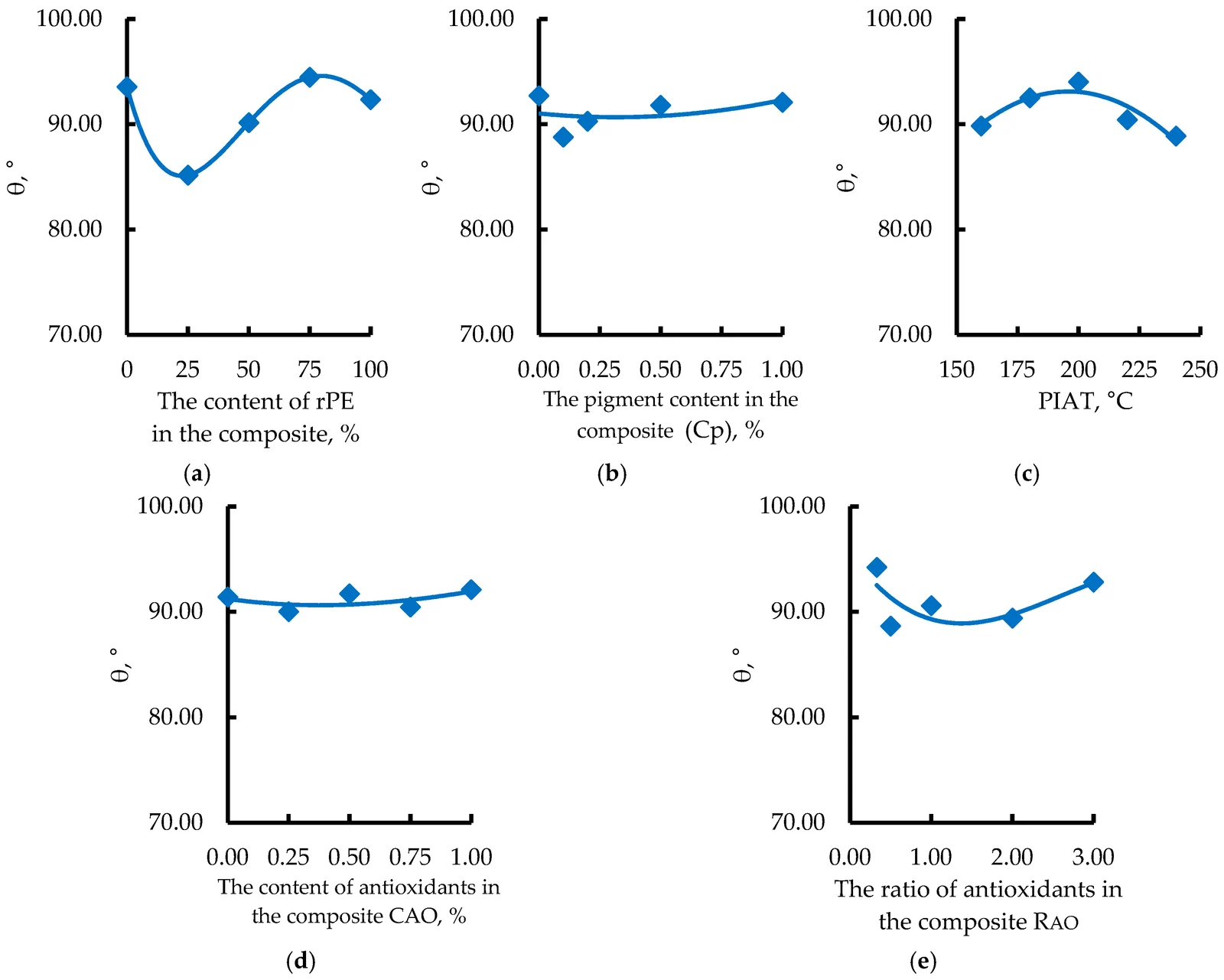
Effects of (**a**) rPE content, (**b**) Cp, (**c**) PIAT, (**d**) CAO, and (**e**) RAO on the contact angle of wettability (θ).

**Figure 13:**
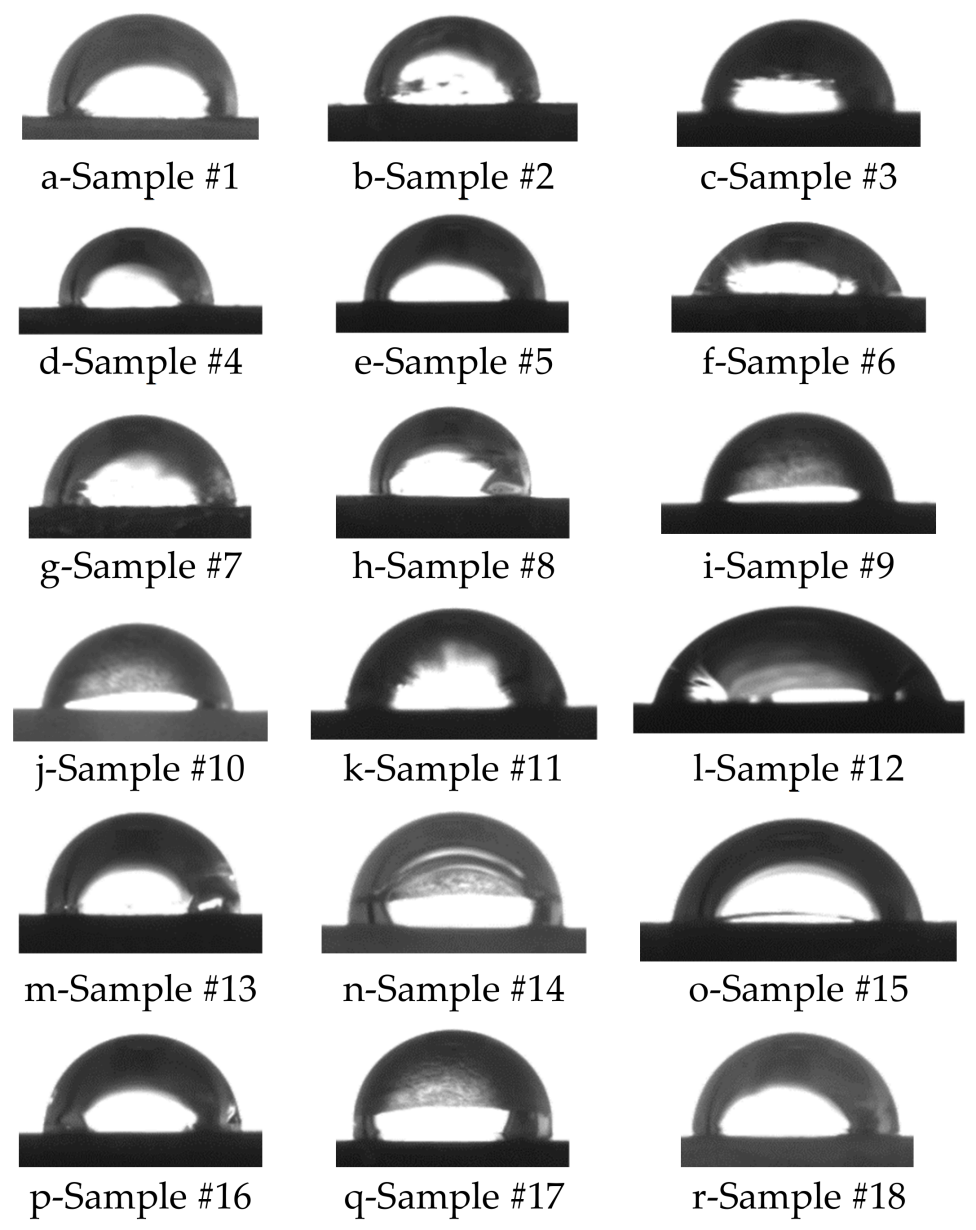

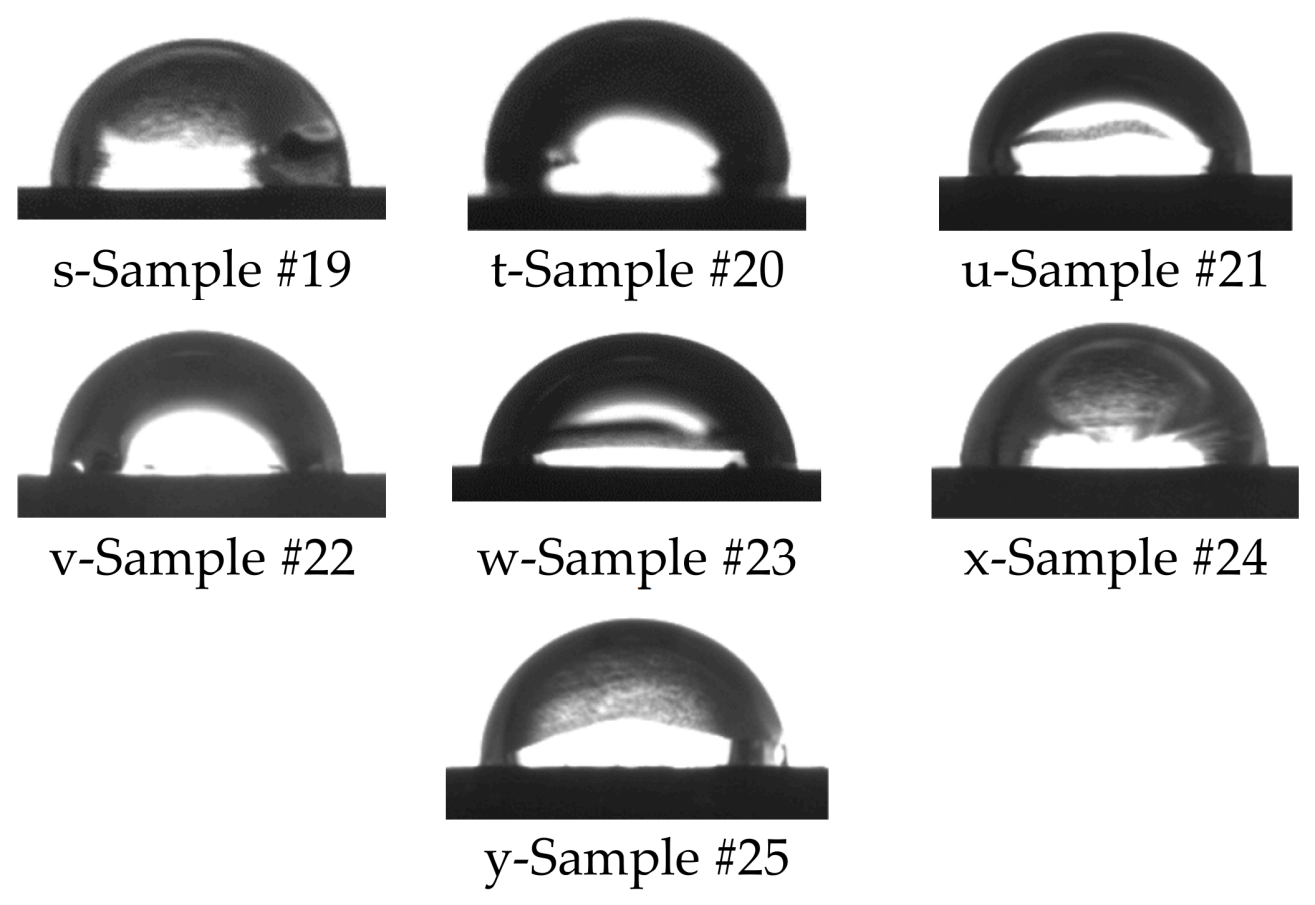
Photographs of distilled water droplets on the twenty-five investigated composite formulations.

**Figure 14:**
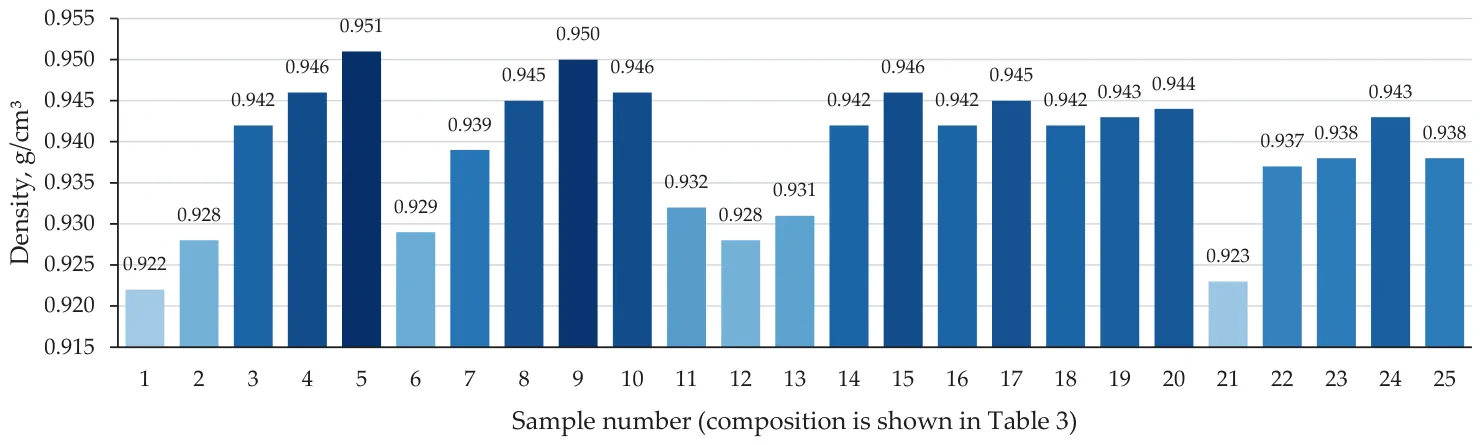
Experimentally determined density of the investigated composites (at 20 ± 1 °C).

**Figure 15:**
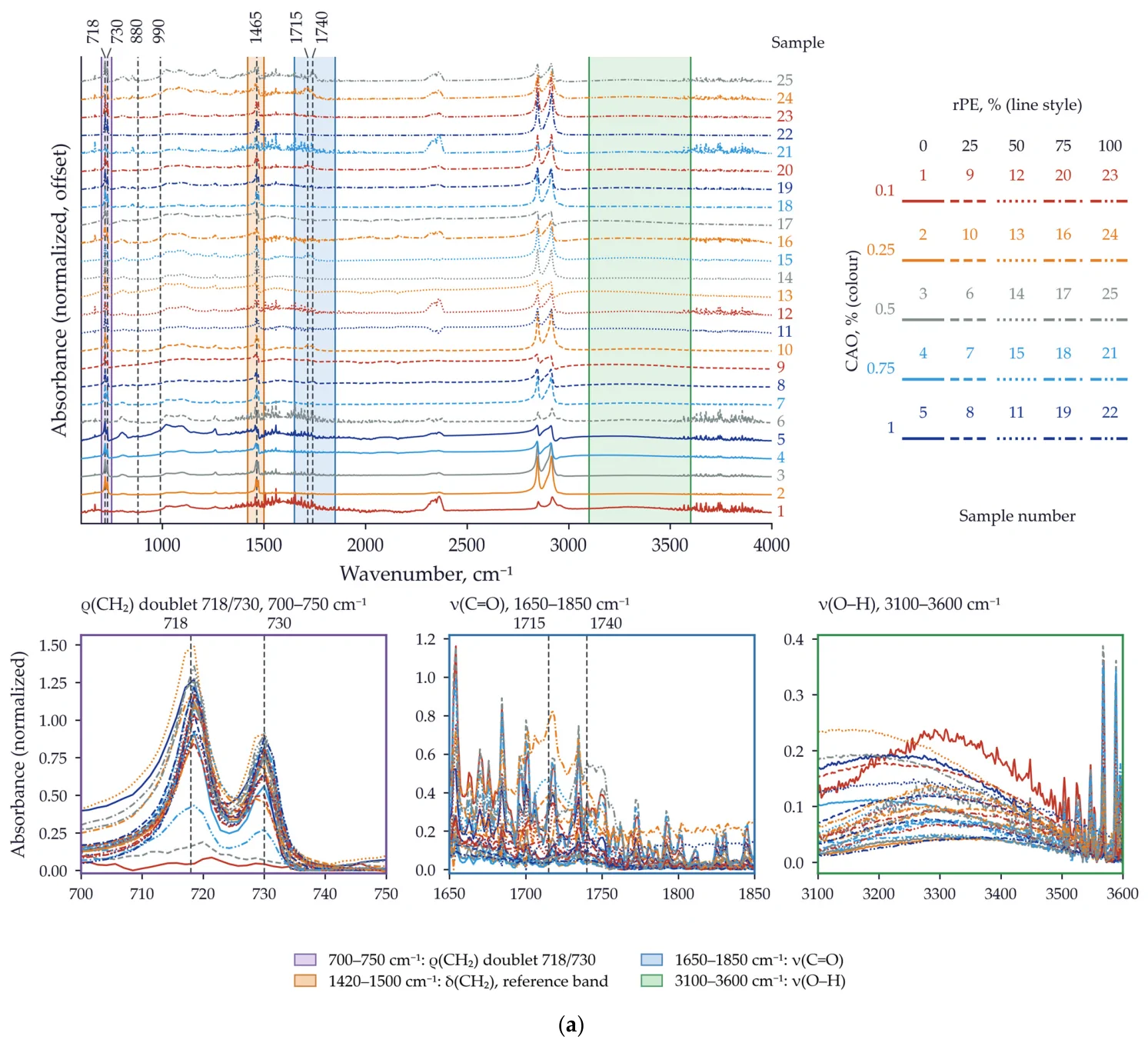

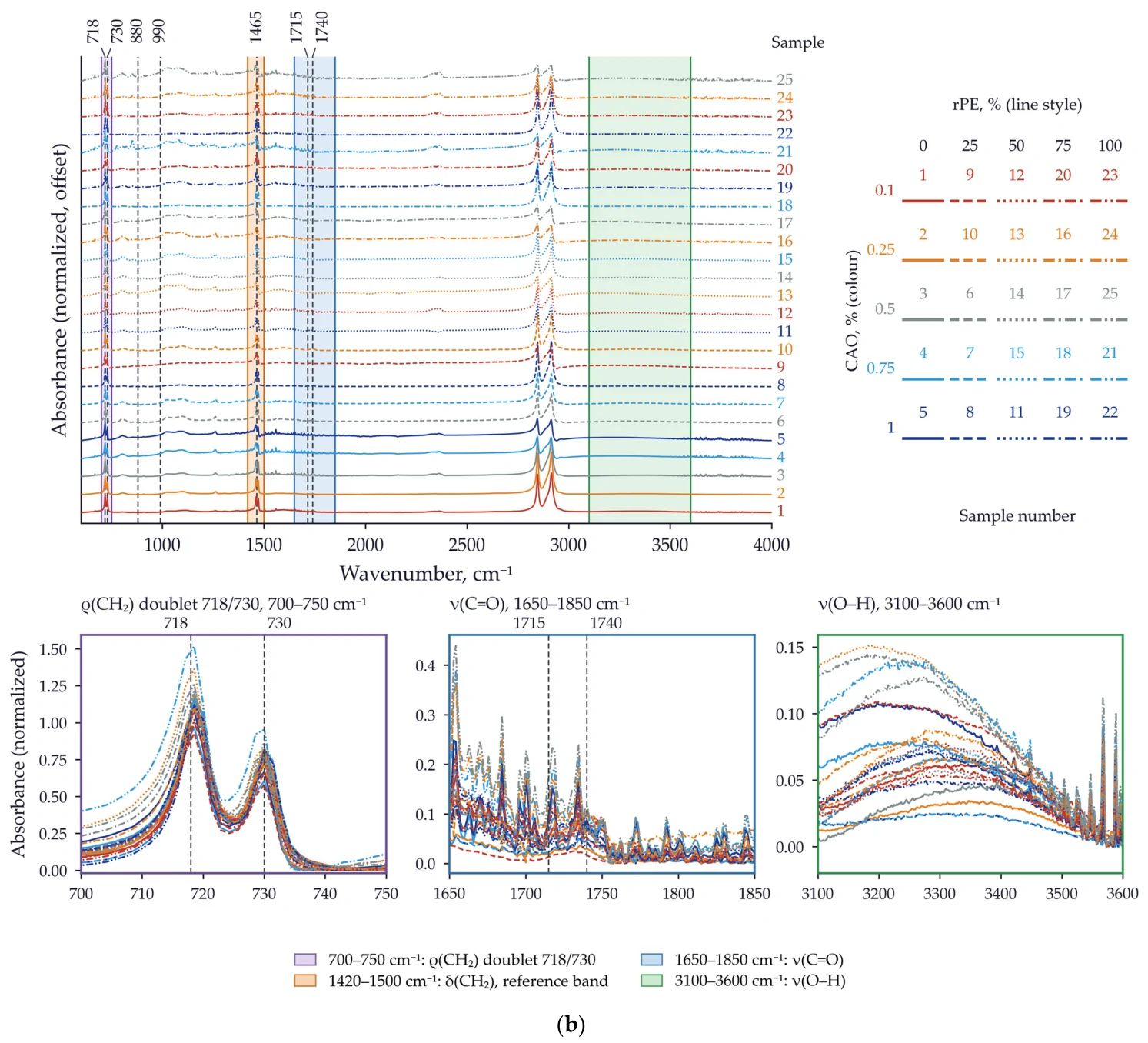

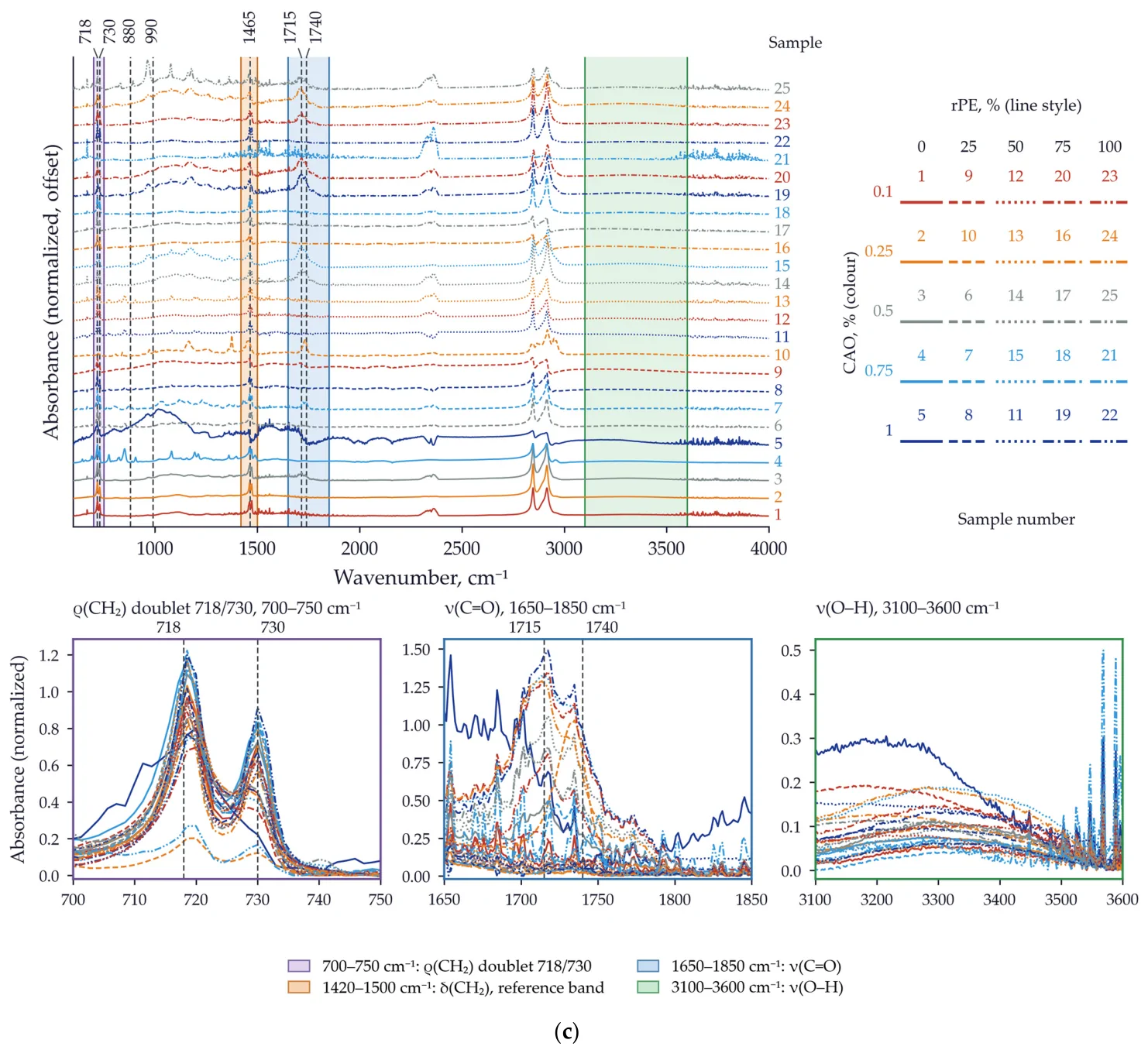

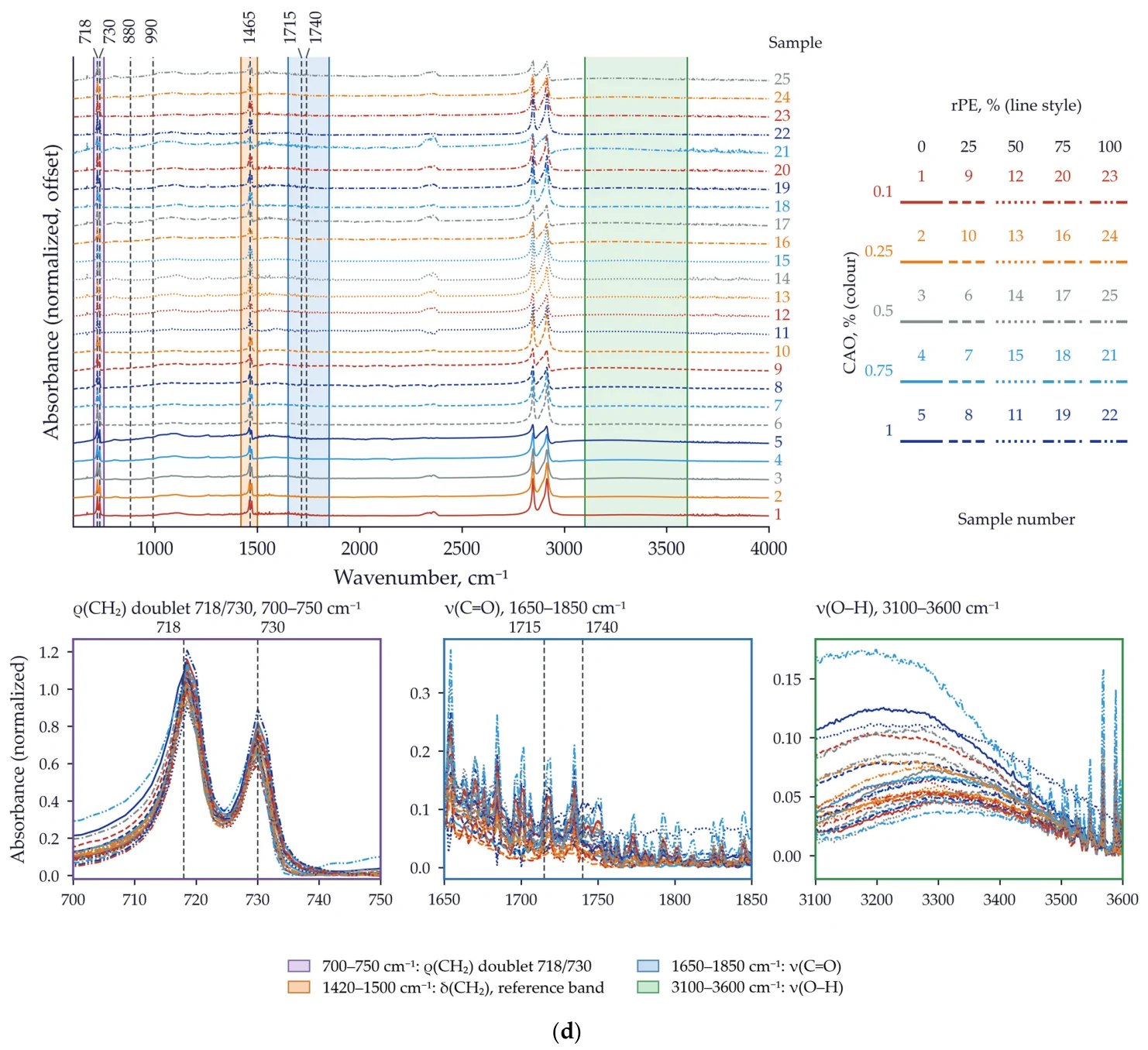

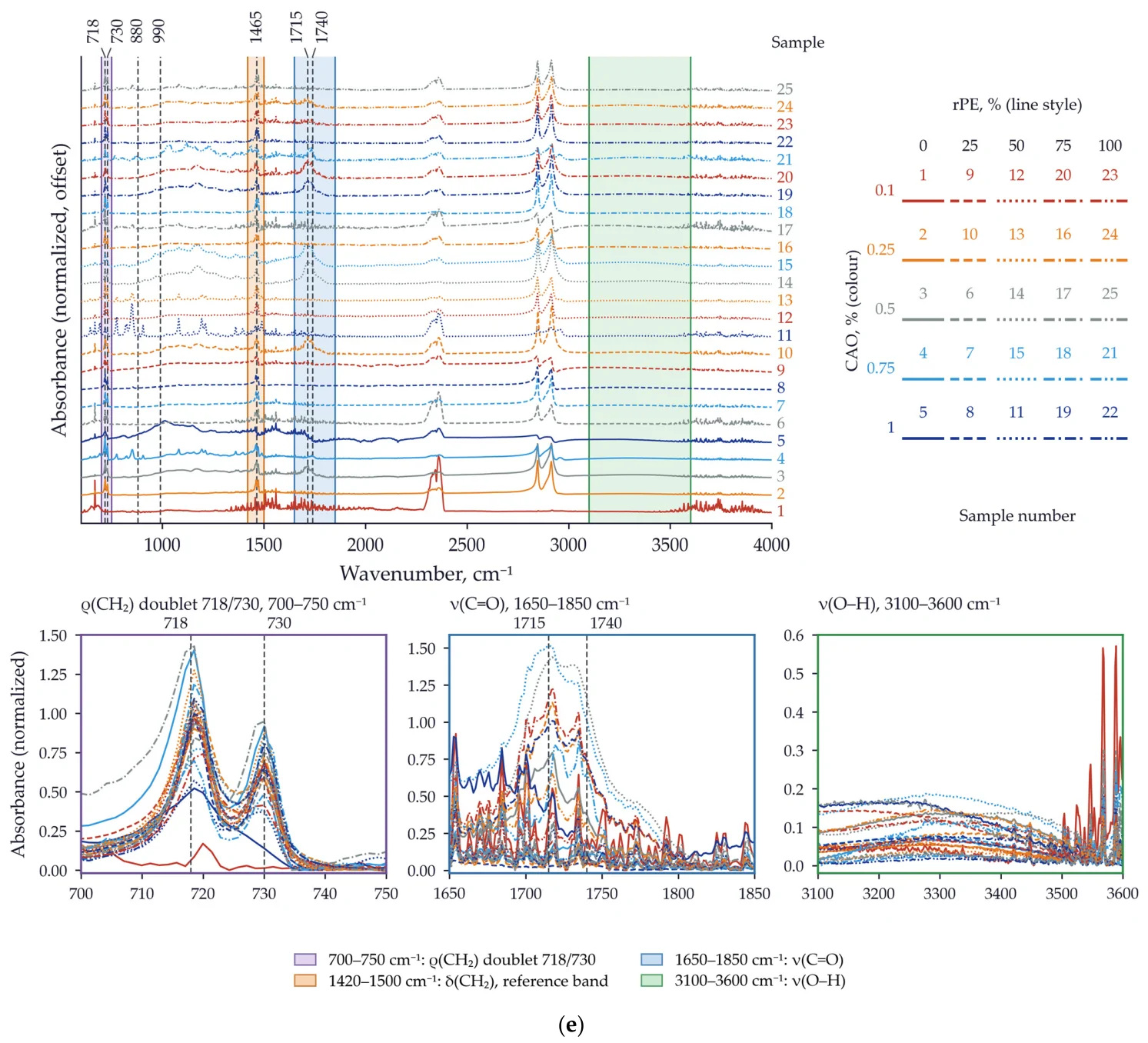

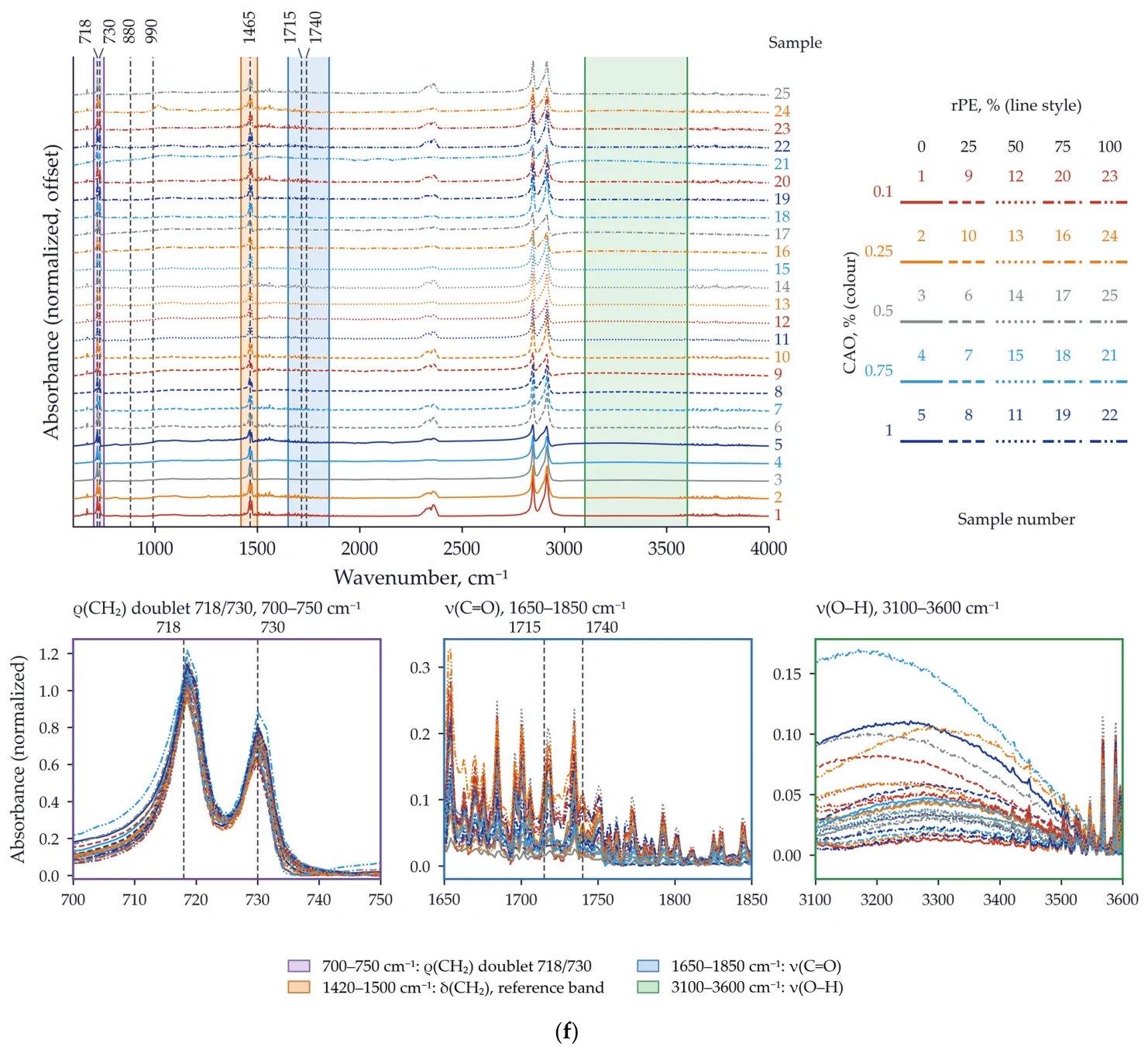
IR spectra of the twenty-five formulations: (**a**,**b**) before testing; (**c**,**d**) after 333 h and (**e**,**f**) after 666 h in the UV chamber; (**a**,**c**,**e**) inner region; (**b**,**d**,**f**) outer region. In each panel, colour denotes CAO and line style denotes rPE (Table 3), and the matrix at the right gives the sample number for each combination. Spectra are baseline-corrected and normalized to the 1420–1500 cm^−1^ band; they are offset vertically in the order of Table 3 in the upper plot and overlaid in the lower plots, which enlarge the 700–750, 1650–1850 and 3100–3600 cm^−1^ regions.

**Figure 16:**
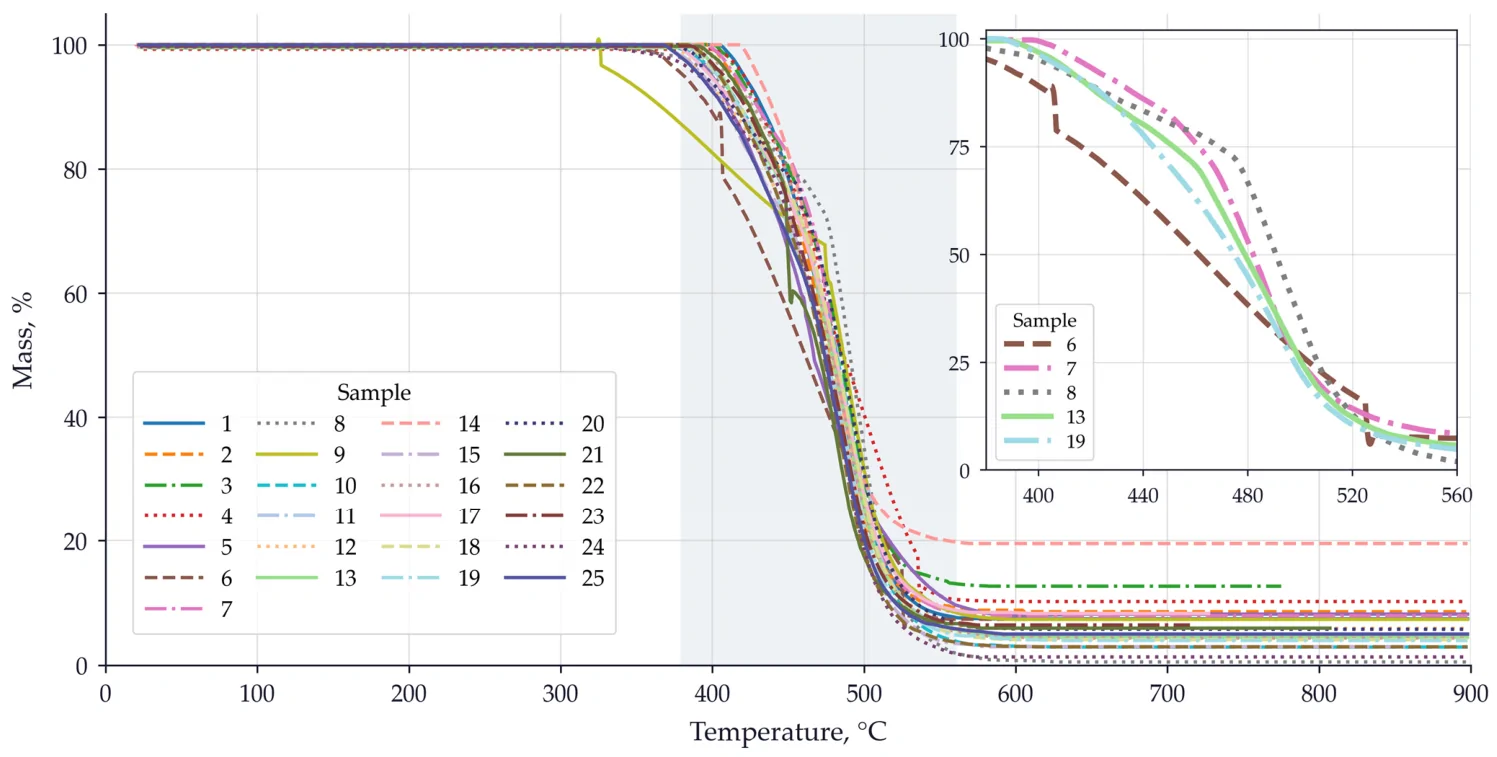
TG thermogram of the twenty-five investigated samples (Table 3). Inset: representative curves in the decomposition range (380–560 °C), samples 6, 19, 13, 7 and 8, which have the lowest, lower-quartile, median, upper-quartile and highest temperature of 50% mass loss, respectively.

**Figure 17:**
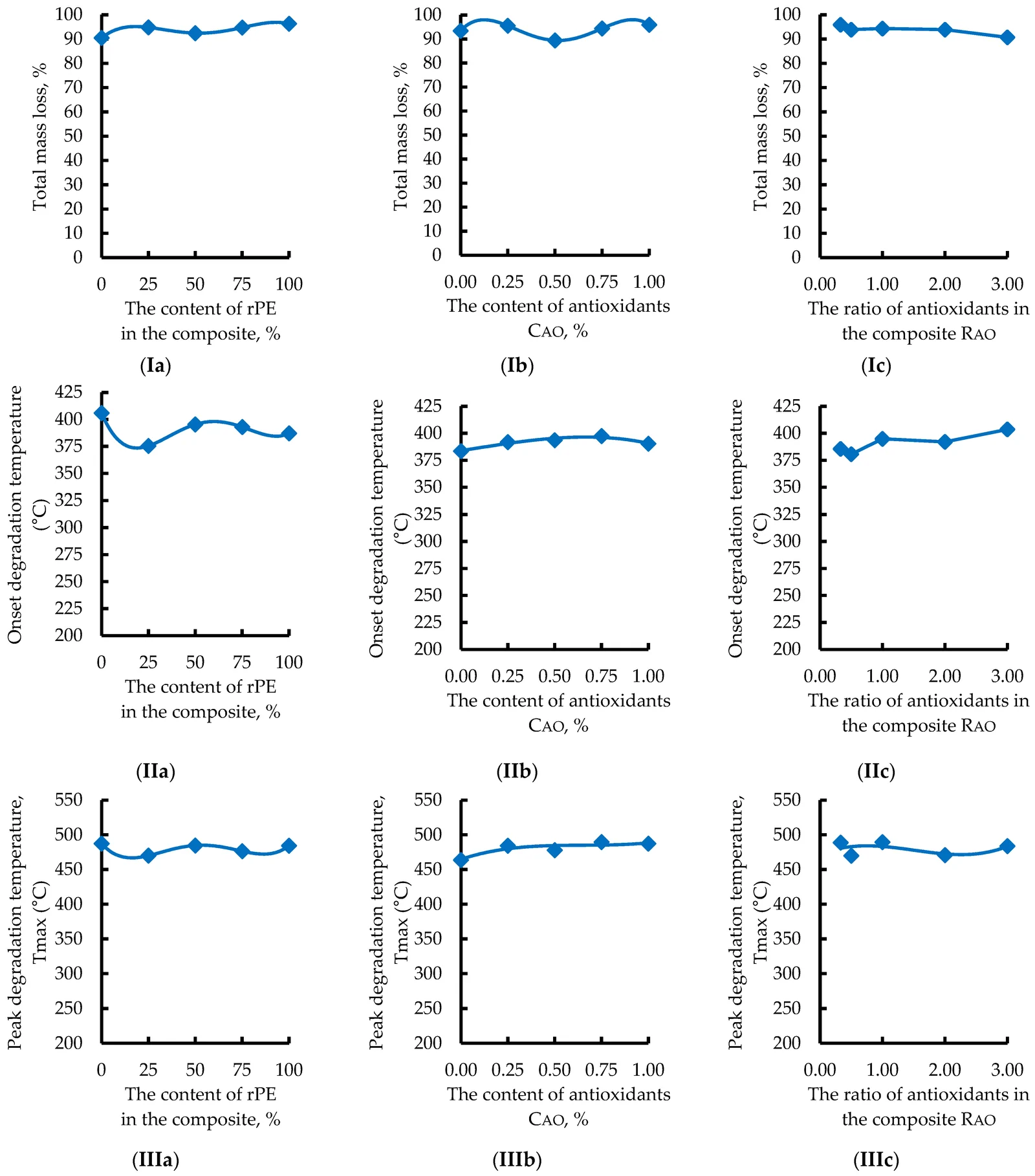
Effects of (**a**) rPE content, (**b**) CAO, and (**c**) RAO on the total mass loss M (**I**), the onset degradation temperature (**II**), and Tmax (**III**).

**Figure 18:**
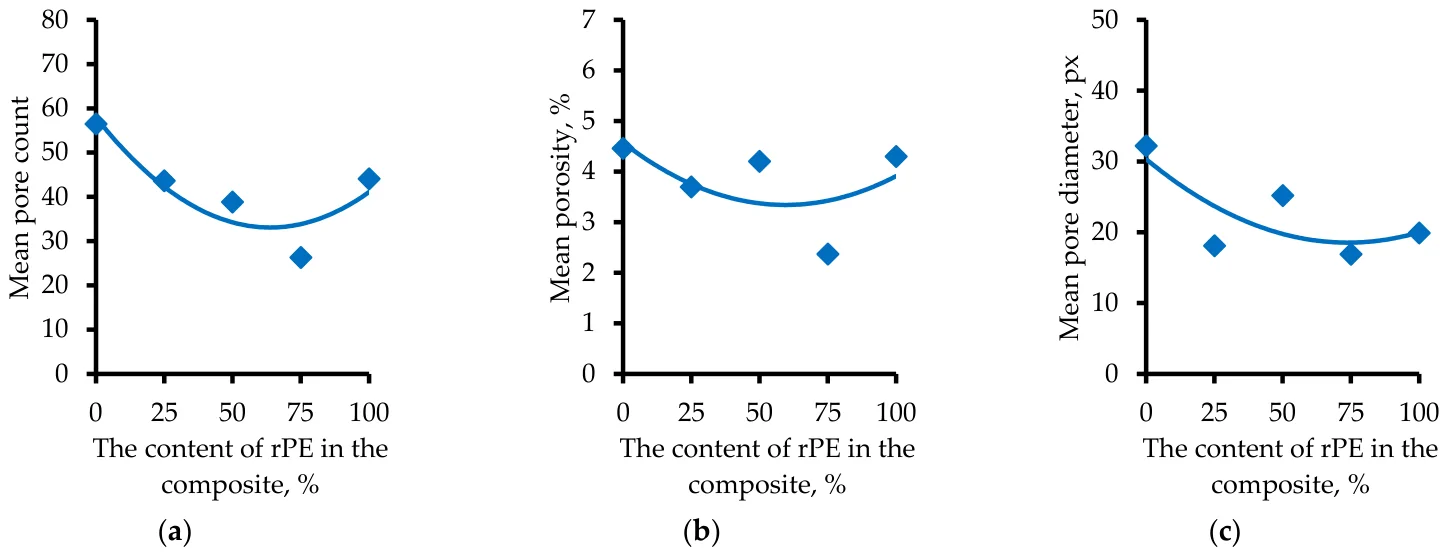
Effect of the rPE content on the mean pore count (**a**), the mean porosity (**b**), and the mean pore diameter (**c**).

**Figure 19:**
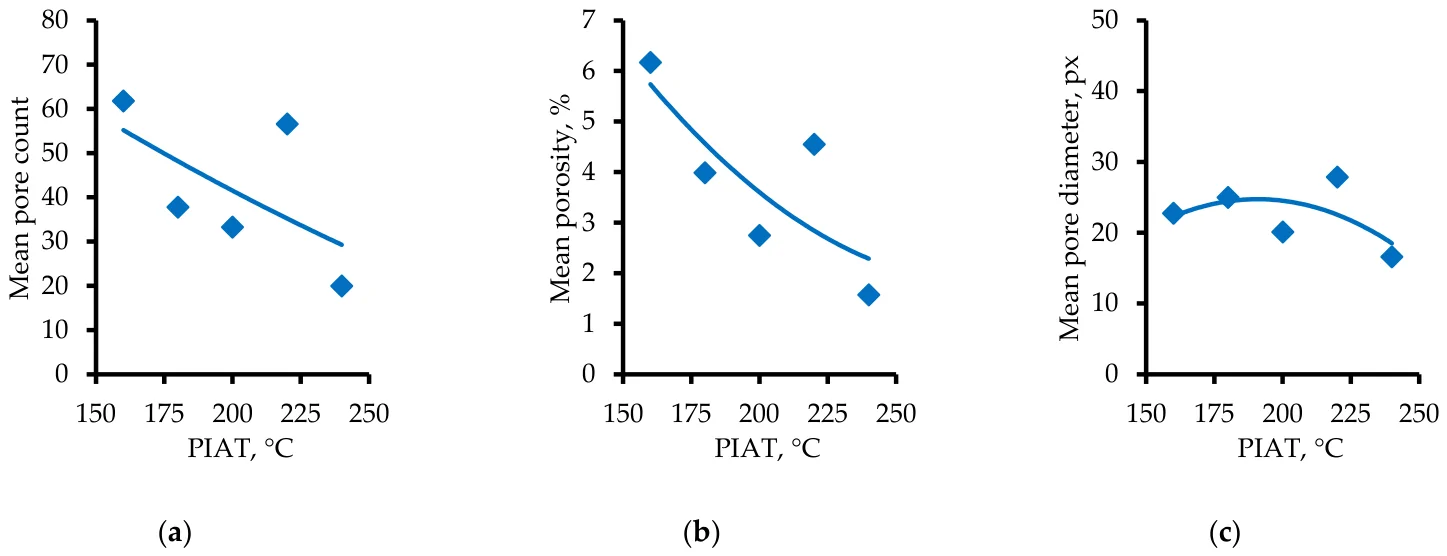
Influence of PIAT on the mean pore count (**a**), the mean porosity, % (**b**), and the mean pore diameter (**c**).

**Figure 20:**
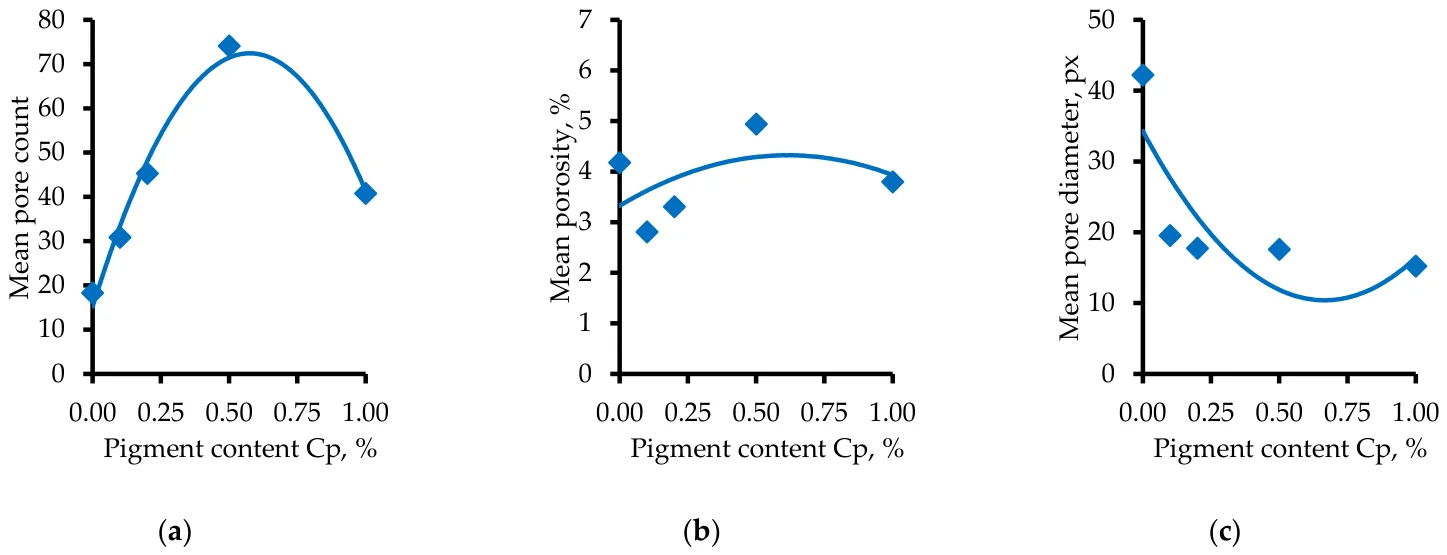
Influence of pigment content (Cp) on the mean pore count (**a**), the mean porosity, % (**b**), and the mean pore diameter (**c**).

**Figure 21:**
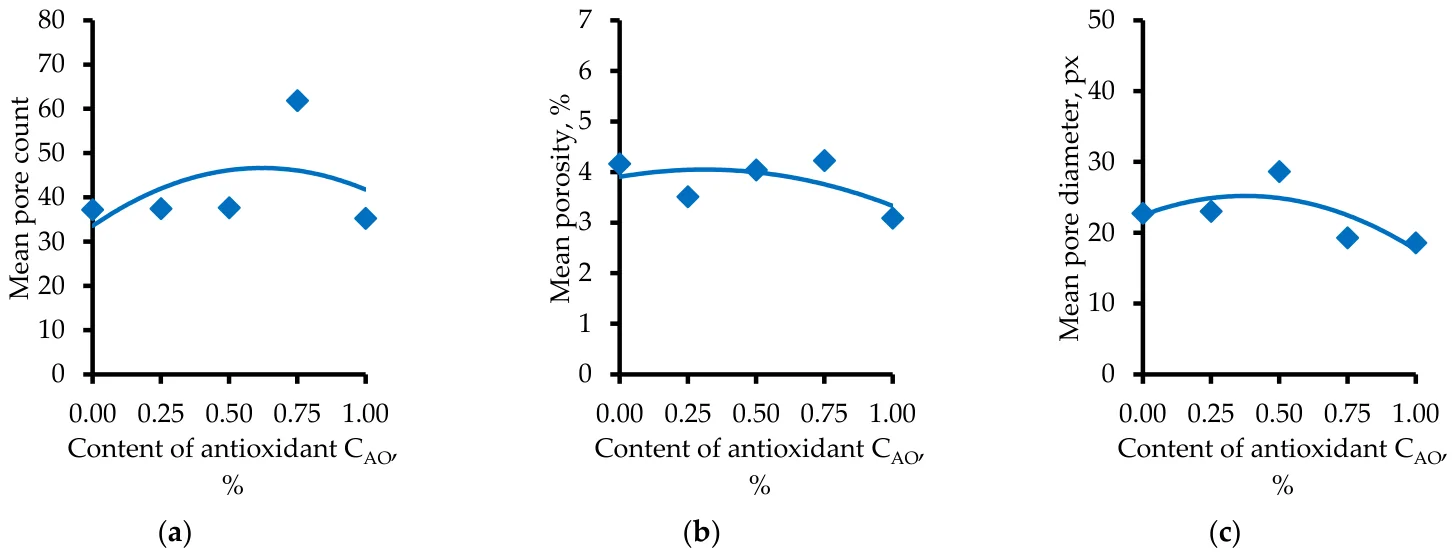
Influence of antioxidant content (CAO) on the mean pore count (**a**), the mean porosity, % (**b**), and the mean pore diameter (**c**).

**Figure 22:**
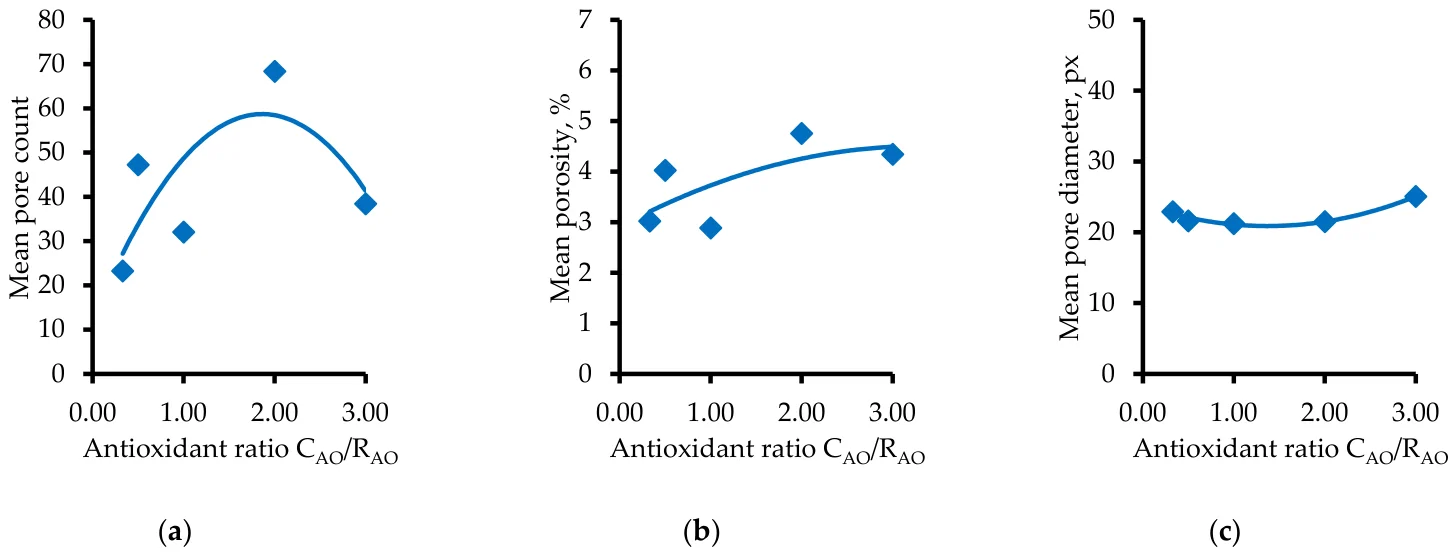
Influence of the antioxidant ratio (CAO/RAO) on the mean pore count (**a**), the mean porosity, % (**b**), and the mean pore diameter (**c**).

**Figure 23:**
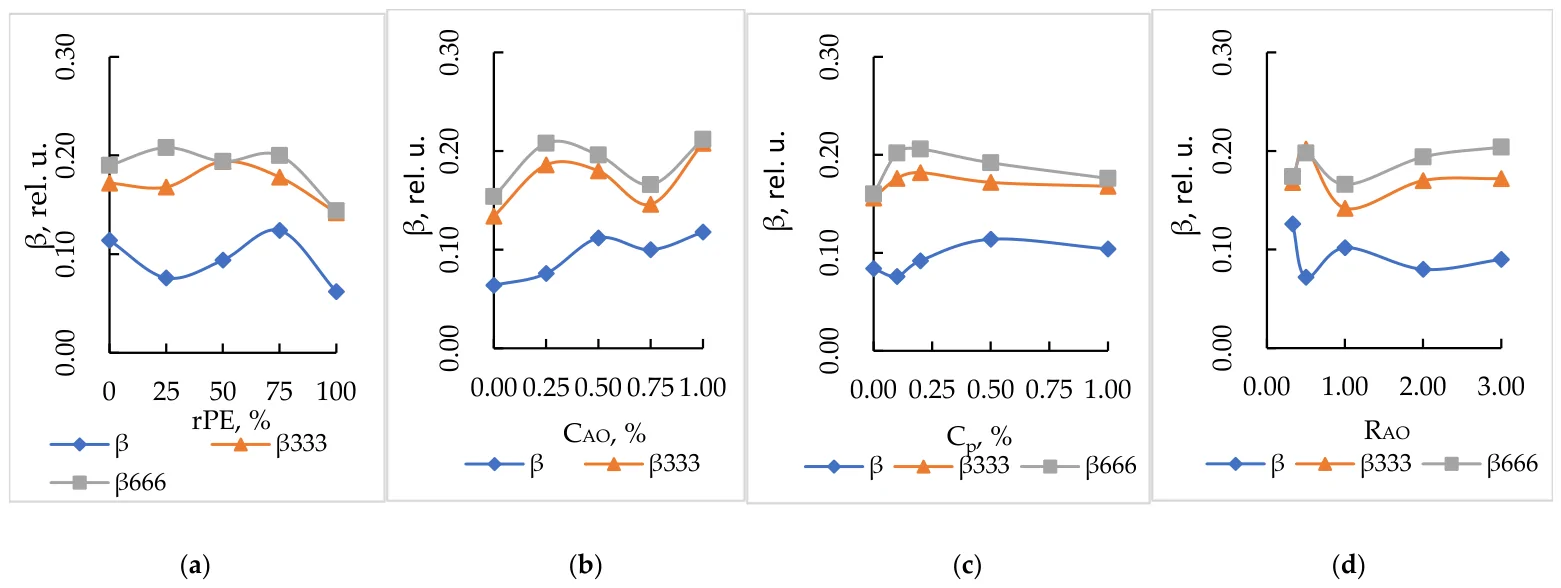
The influence of rPE (**a**), CAO (**b**), Cp (**c**), and RAO (**d**) on β after 333 and 666 h of holding in the UV chamber.

**Figure 24:**
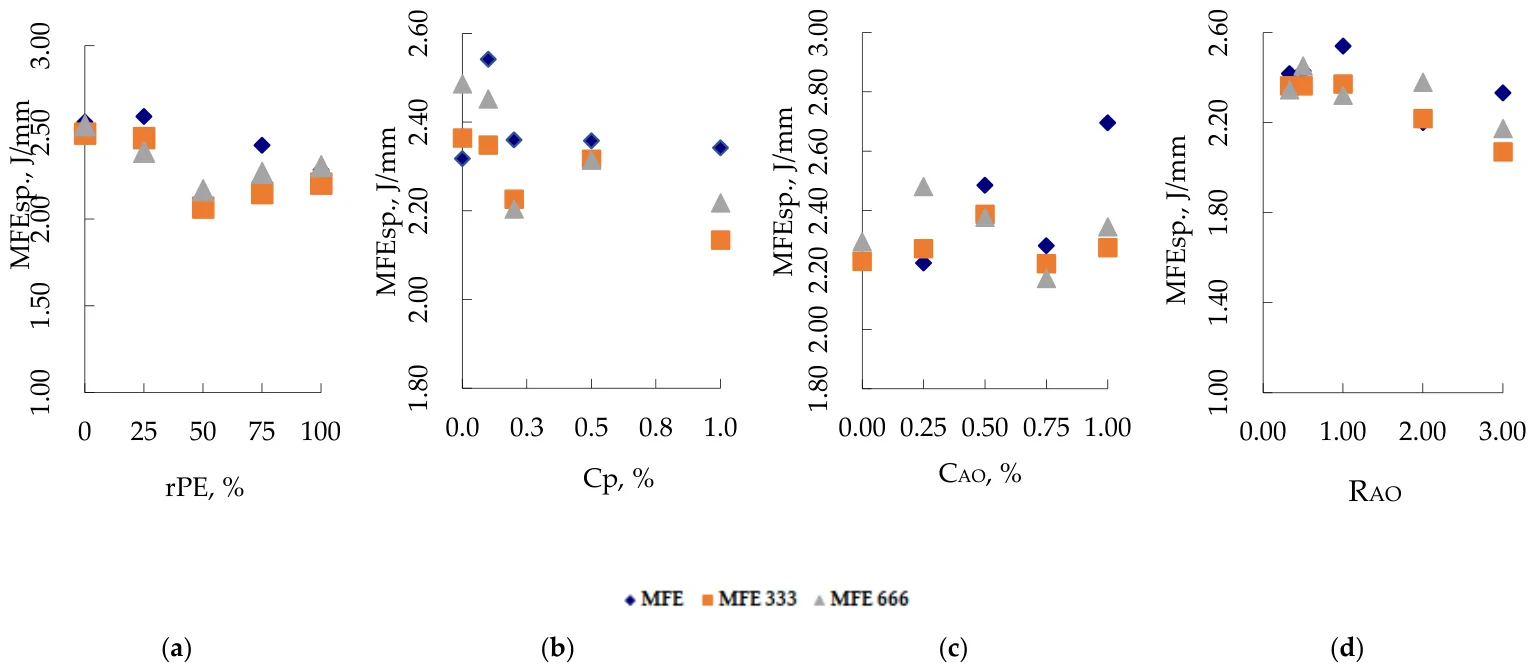
The influence of rPE (**a**), Cp (**b**), CAO (**c**), and RAO (**d**) on MFE (J/mm), after 333 and 666 h of holding in the UV chamber.

**Figure 25:**
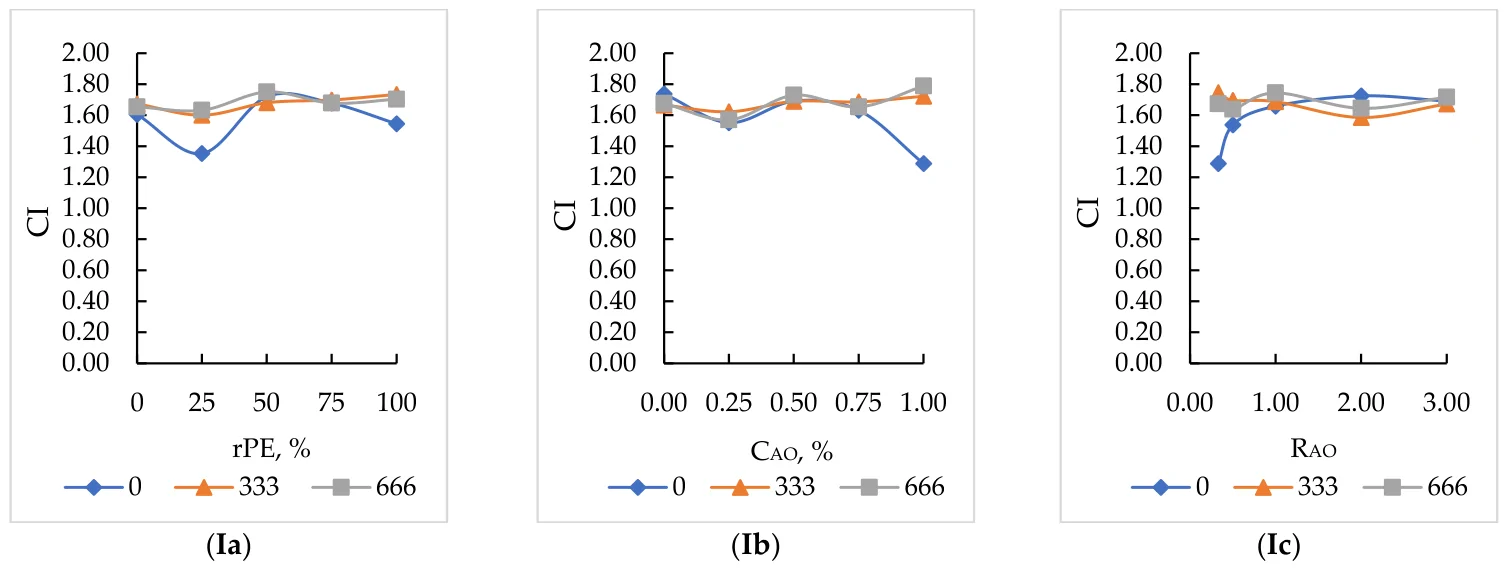

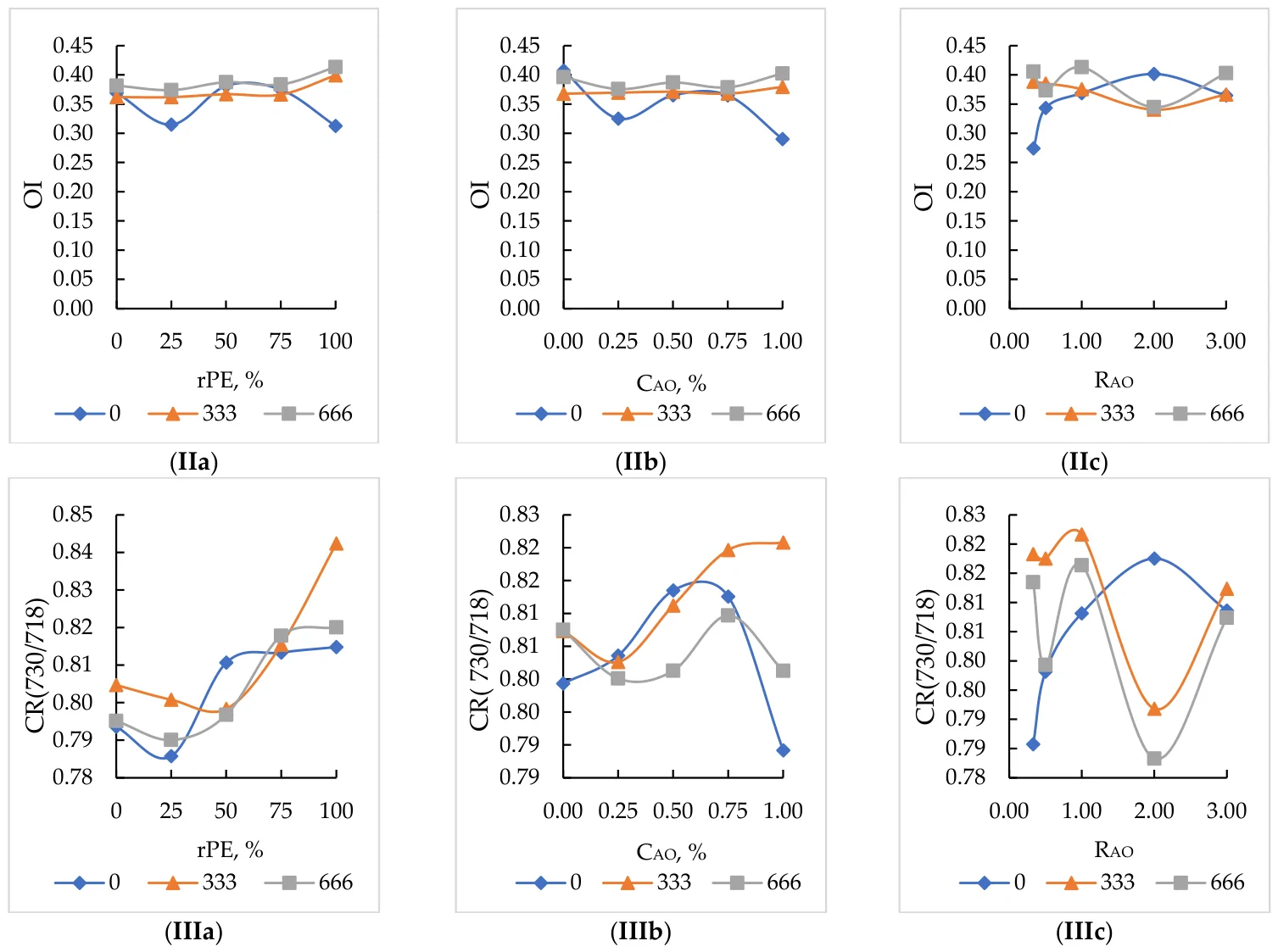
Effects of (**a**) rPE content, (**b**) CAO, and (**c**) RAO on the carbonyl (**I**), hydroxyl (**II**), and crystallinity (**III**) indices.

**Figure 26:**
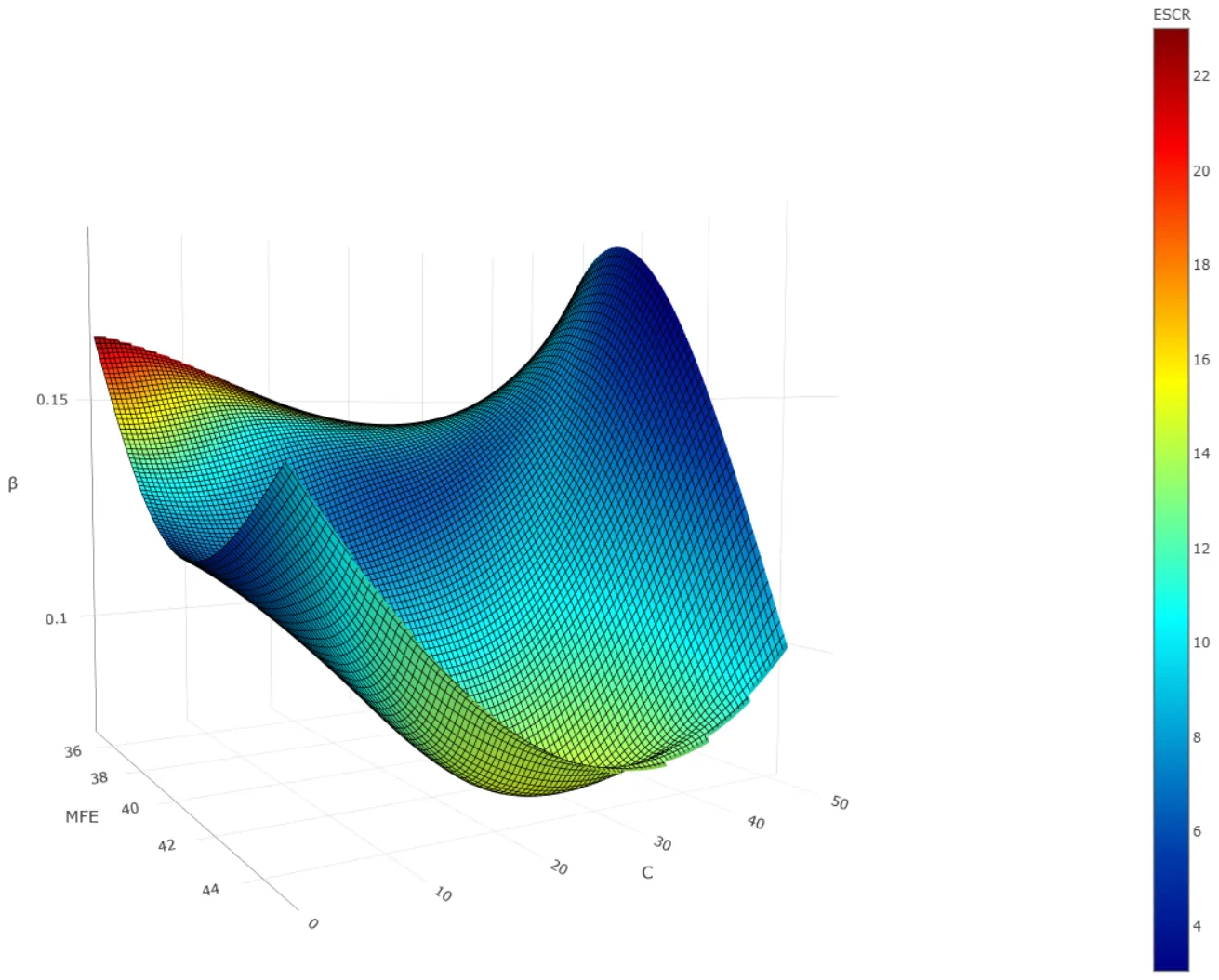
Correlation among MFE, ESCR, β, and the content of recycled polyethylene (rPE) in the studied rotational composites. Axes: C—rPE content, %; MFE—mean failure energy, J; β—third-harmonic amplitude, rel. u.; color scale—environmental stress cracking resistance (ESCR), h. Reproduced without changes from [24] (© 2025 by the authors; licensee MDPI, Basel, Switzerland), licensed under CC BY 4.0 (https://creativecommons.org/licenses/by/4.0/).

**Figure 27:**
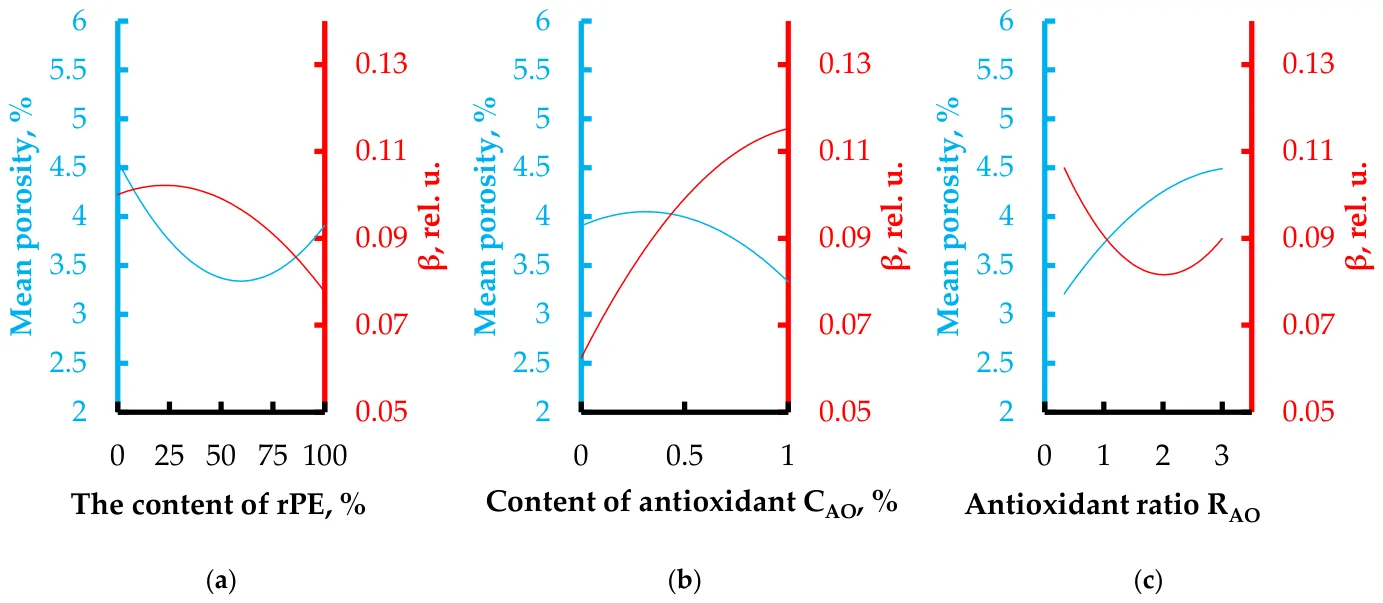
Correlation plot among mean porosity, %, and β, rel. u., under the different influence of the factors (**a**) rPE, (**b**) CAO, and (**c**) RAO.

**Figure 28:**
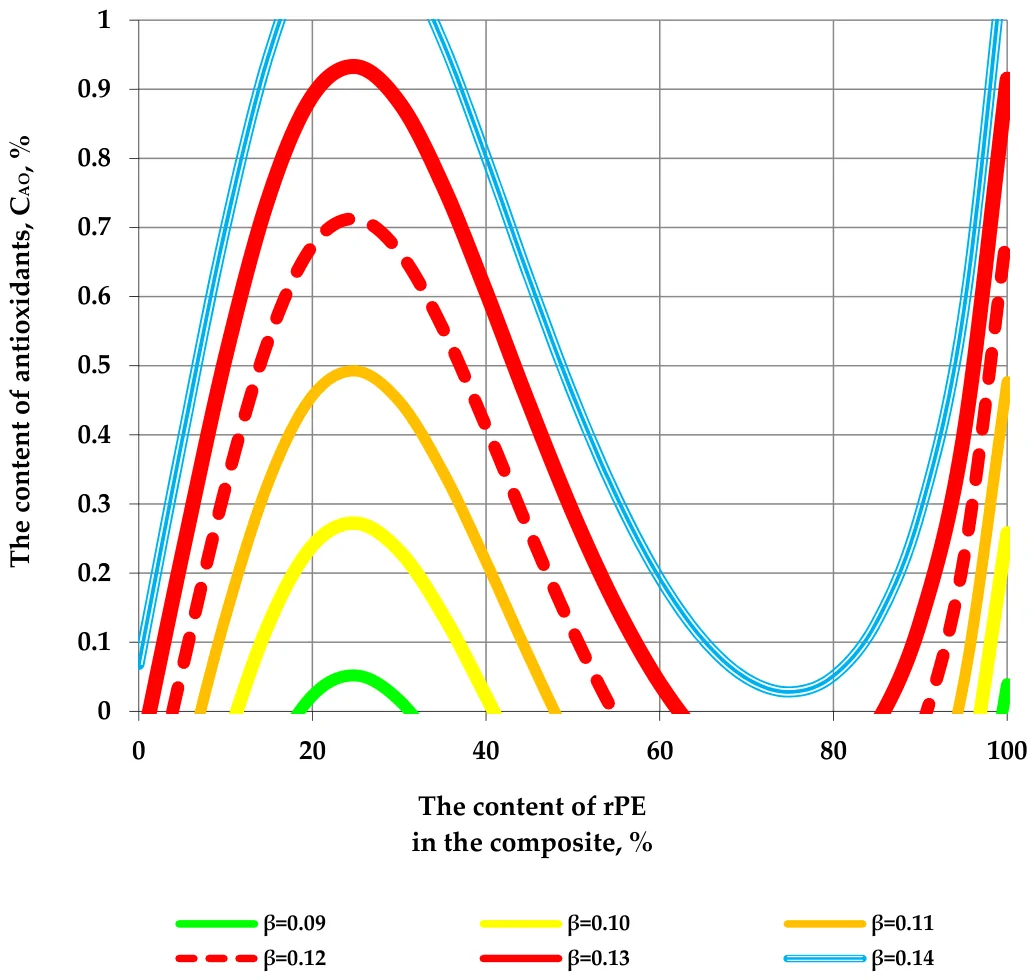
Two-factor nomogram: CAO = f(β, rPE) for the recommended formulation—for service without UV radiation / underground septic tanks (Cp = 1%, RAO = 1).

**Table 1:** Stages of the operating cycle of the UV-aging chamber.

Parameter	Stage 1: UV Irradiation	Stage 2: Condensation
Duration	8 h	4 h
Lamp type	UVB-313EL	—
Irradiance at 313 nm	0.71 W/m^2^	0 (lamps off)
Black-panel temperature (BPT)	60 ± 3 °C	50 ± 3 °C
Humidity	Not controlled	≥90% RH (condensation)
Total cycle	12 h (8 + 4)	Repeated continuously

**Table 2:** Correspondence between the duration of exposure in the UV chamber and the conditional service horizon for four climatic locations of Kazakhstan.

Total Cyclic Time in the Chamber, h	incl. UV Phase, h	Conditional Degradation Horizon			
		Almaty ($K_{eff}$≈ 106), Years	Astana ($K_{eff}$≈ 127), Years	Shymkent ($K_{eff}$≈ 97), Years	Petropavlovsk ($K_{eff}$≈ 132), Years
333	222	4.0	4.8	3.7	5.0
666	444	8.1	9.7	7.4	10.0

**Table 3:** Probabilistic–deterministic experimental design for the factors rPE, Cp, CAO, RAO, and PIAT, and the mechanical, thermal, and acoustic responses of the composites.

	Optimization Parameters					Experimental Characteristics Obtained for the Composites											
						Acoustic Properties			Mechanical Properties					Contact Angle of Wettability (θ), Degrees	Thermal Properties of the Obtained Composites		
	The Content of Secondary Rotational Polyethylene (rPE) in the Composite, %	The Content of Pigment in the Composite, %	The Content of Antioxidants in the Composite, %	The Ratio of Antioxidants, RAO	PIAT, °C	Third Harmonic Crest Factor, β, rel. u.	Amplitude of the Transmitted Signal, A	Amplitude Coefficient of the Fifth Harmonic, λ	Tensile Strength at Break, MPa	Yield Strength, MPa	Elongation at Break, %	Tensile Modulus of Elasticity, E, MPa	Specific Mean Failure Energy, MFE_sp._, J/mm		Onset Temperature of Thermal Degradation, T_b_ (°C)	Temperature of Maximum Mass Loss, T_max_ (°C)	Percentage of Mass Loss of the Sample, M (%)
1	0	0	0.1	1:3	160	0	90	0.000	15.0	15	330	264	2.58	84.25	413.00	486.30	92.56
2	0	0.1	0.25	1:2	180	0.23	65	0.000	17.0	17	617	260	2.73	92.89	403.80	475.50	90.56
3	0	0.2	0.5	1:1	200	0.25	140	0.500	17.8	13	413	310	3.01	92.24	410.50	488.10	87.32
4	0	0.5	0.75	2:1	220	0.21	133	0.400	17.8	14	66	275	2.03	92.9	410.80	494.60	89.78
5	0	1	1	3:1	240	0.26	134	0.300	176	6	47	308	2.45	90.8	391.40	491.90	91.80
6	25	0.1	0.5	2:1	160	0.15	111	0.000	17.4	11	47	280	2.5	76.24	367.60	454.10	92.62
7	25	0.2	0.75	3:1	180	0.19	106	0.000	17.9	9	239	320	2.85	88.44	406.50	484.90	92.06
8	25	0.5	1	1:3	200	0.25	131	0.100	19.0	17	357	360	3.2	96.7	378.00	492.90	99.48
9	25	1	0.1	1:2	220	0.23	136	0.200	18.5	7	162	300	2.38	84.65	335.30	424.90	92.64
10	25	0	0.25	1:1	240	0.22	134	0.100	16.3	10	737	280	2.03	84.59	389.90	493.20	97.08
11	50	0.2	1	1:2	160	0.16	128	0.000	16.7	7	38	239	2.06	88.26	392.10	477.90	95.08
12	50	0.5	0.1	1:1	180	0.14	129	0.000	16.2	10	52	220	2.1	77	383.30	489.30	94.07
13	50	1	0.25	2:1	200	0.21	128	0.000	15.0	3	15	360	2.27	92.9	396.10	488.80	95.52
14	50	0	0.5	3:1	220	0.22	133	0.100	14.8	8	471	350	2.05	97.19	423.90	477.70	80.47
15	50	0.1	0.75	1:3	240	0.24	135	0.300	17.1	11	135	223	1.81	84.58	381.20	489.80	97.03
16	75	0.5	0.25	3:1	160	0.18	129	0.000	18.6	13	29	232	2.01	91.03	400.30	478.80	95.64
17	75	1	0.5	1:3	180	0.16	136	0.300	15.7	6	48	260	2.42	102.24	386.70	490.20	91.69
18	75	0	0.75	1:2	200	0.19	131	0.100	15.2	10	219	252	2.53	91.63	392.90	491.80	95.89
19	75	0.1	1	1:1	220	0.22	136	0.200	16.5	6	74	265	3.37	93.58	394.80	489.50	96.03
20	75	0.2	0.1	2:1	240	0.23	131	0.000	16.3	7	109	280	1.8	104.16	390.00	431.80	94.22
21	100	1	0.75	1:1	160	0	56	0.000	15.0	6	20	273	2.19	94.69	395.70	486.80	97.05
22	100	0	1	2:1	180	0.17	129	0.000	15.6	8	166	267	2.4	91.15	395.70	484.70	97.05
23	100	0.1	0.1	3:1	200	0.17	131	0.000	17.4	10	181	270	2.3	89.42	396.10	485.50	93.55
24	100	0.2	0.25	1:3	220	0.2	129	0.100	18.0	7	179	200	2.08	88.65	369.00	484.90	98.67
25	100	0.5	0.5	1:2	240	0.18	128	0.000	15.9	8	34	323	2.45	90.57	379.10	479.60	95.00

## Data Availability

The original contributions presented in this study are included in the article. Further inquiries can be directed to the corresponding author.

## References

[1] PlasticsEurope (2026). The Circular Economy for Plastics — A European Analysis 2026.

[2] OECD (2024). Policy Scenarios for Eliminating Plastic Pollution by 2040.

[3] OECD (2022). Global Plastics Outlook: Economic Drivers, Environmental Impacts and Policy Options.

[4] Zhaparova S.B., Bayazitova Z.E., Bekpergenova Z.B. (2020). Use of plastic waste in the production of polymer-sanded tiles. Vestnik of M. Kozybayev North Kazakhstan Univ..

[5] Khassenova A.K., Kuantkan B. (2023). From waste to wealth: International experience in improving the processing of production and consumption waste. Vestnik of M. Kozybayev North Kazakhstan Univ..

[6] PlasticsEurope (2023). Plastics—the Fast Facts 2023.

[7] Geyer R., Jambeck J.R., Law K.L. (2017). Production, Use, and Fate of All Plastics Ever Made. Sci. Adv..

[8] Andrady A.L. (2015). Plastics and Environmental Sustainability.

[9] Eriksen M., Lebreton L.C.M., Carson H.S., Thiel M., Moore C.J., Borerro J.C., Galgani F., Ryan P.G., Reisser J. (2014). Plastic Pollution in the World’s Oceans: More than 5 Trillion Plastic Pieces Weighing over 250,000 Tons Afloat at Sea. PLoS ONE.

[10] Amadei A., Ardente F. (2022). Modelling Plastic Flows in the European Union Value Chain.

[11] World Economic Forum (2021). Global Plastic Action Partnership: 2021 Annual Impact Report.

[12] Crawford R.J., Throne J.L. (2002). Rotational Molding Technology.

[13] Sharifi P., Henwood N., Liauw C.M., Lees G., Quaratino A. (2012). Studies of degradation effects during rotational moulding. SPE ANTEC Tech. Pap..

[14] Grand View Research (2023). Recycled Polyethylene Market Size, Share & Trends Analysis Report, 2023–2030.

[15] Kelly-Walley J., Martin P., Ortega Z., Pick L., McCourt M. (2024). Recent Advancements towards Sustainability in Rotomoulding. Materials.

[16] Crawford R.J., Kearns M.P. (2012). Practical Guide to Rotational Moulding.

[17] Liu S.-J., Ho C.-Y. (2000). Optimizing the impact strength of rotationally moulded parts. Polym. Eng. Sci..

[18] Ghosh A. (2021). Performance modifying techniques for recycled thermoplastics. Resour. Conserv. Recycl..

[19] Songwon Industrial Co., Ltd. (2013). Processing Additives and Uses of Same in Rotational Molding. EP.

[20] Campanale C., Savino I., Massarelli C., Uricchio V.F. (2023). Fourier Transform Infrared Spectroscopy to Assess the Degree of Alteration of Artificially Aged and Environmentally Weathered Microplastics. Polymers.

[21] Chaisrichawla S., Dangtungee R. (2018). The Usage of Recycled Material in Rotational Molding Process for Production of Septic Tank. Mater. Sci. Forum.

[22] Pick L., Hanna P.R., Gorman L. (2022). Assessment of processibility and properties of raw post-consumer waste polyethylene in the rotational moulding process. J. Polym. Eng..

[23] Tyukanko V., Demyanenko A., Semenyuk V., Dyuryagina A., Alyoshin D., Tarunin R., Voropaeva V. (2023). Development of an Ultrasonic Method for the Quality Control of Polyethylene Tanks Manufactured Using Rotational Molding Technology. Polymers.

[24] Tyukanko V., Tarunin R., Demyanenko A., Semenyuk V., Dyuryagina A., Merkibayev Y., Bakibaev A., Alpyssov R., Alyoshin D. (2025). Optimization of Composite Formulation Using Recycled Polyethylene for Rotational Molding. Polymers.

[25] Mihelčič M., Oseli A., Huskić M., SlemenikPerše L. (2022). Influence of stabilization additive on rheological, thermal and mechanical properties of recycled polypropylene. Polymers.

[26] Pielichowski K., Njuguna J., Majka T.M. (2022). Thermal Degradation of Polymeric Materials.

[27] (2008). Plastics—Differential Scanning Calorimetry (DSC)—Part 6: Determination of Oxidation Induction Time (Isothermal OIT) and Oxidation Induction Temperature (Dynamic OIT).

[28] Plastics Technology Melt Flow Rate Testing—Part 6: Increase in MFR as Indicator of Polymer Degradation. Plastics Technology Online, December 2013. https://www.ptonline.com/articles/melt-flow-rate-testingpart-6-.

[29] Hinsken H., Moss S., Pauquet J.-R., Zweifel H. (1991). Degradation of polyolefins during melt processing. Polym. Degrad. Stab..

[30] Zweifel H., Maier R.D., Schiller M. (2009). Plastics Additives Handbook.

[31] Allen N.S., Hoang E., Liauw C.M., Edge M., Fontan E. (2001). Influence of processing aids on the thermal and photostabilisation of HDPE with antioxidant blends. Polym. Degrad. Stab..

[32] Pospíšil J., Nešpůrek S., Hamid S.H. (2000). Highlights in the inherent chemical activity of polymer stabilizers. Handbook of Polymer Degradation.

[33] Cramez M.C., Oliveira M.J., Crawford R.J. (2002). Optimisation of rotational moulding of polyethylene by predicting antioxidant consumption. Polym. Degrad. Stab..

[34] Emaldi I., Liauw C.M., Potgieter H. (2015). Stabilization of Polypropylene for Rotational Molding Applications. J. Res. Updates Polym. Sci..

[35] Weick B.L., Al-Zubi R.S. (2004). Antioxidant Presence in Thick-Walled High-Density Polyethylene Materials after Rotational Molding. J. Appl. Polym. Sci..

[36] Kelly-Walley J., Quaratino A., Jayawardane D. (2024). Enhancing Sustainability in Rotational Molding: Evaluating Stabilization Effects on PCR/Virgin Polyethylene Blend Performance. Proceedings of the SPE ANTEC 2024.

[37] Fatnes A.M., Øysæd H., Frøhaug A., Jamtvedt S., Hoffmann K. (2004). Process for Addition of Additives to Polymer Particles. EP.

[38] Pospíšil J., Habicher W.-D., Pilař J., Nešpůrek S., Kuthan J., Piringer O.-G., Zweifel H. (2002). Discoloration of polymers by phenolic antioxidants. Polym. Degrad. Stab..

[39] BASF SE (2010). Irganox B 215: Synergistic Processing and Long-Term Thermal Stabilizer System (Blend of Irgafos 168 and Irganox 1010).

[40] Hallstein J., Metzsch-Zilligen E., Pfaendner R. (2024). Enhancing the Hydrolytic Stability of Poly(lactic acid) Using Novel Stabilizer Combinations. Polymers.

[41] (2021). Standard Test Method for Environmental Stress-Cracking of Ethylene Plastics.

[42] Dou Y., Rodrigue D. (2022). Morphological, thermal and mechanical properties of recycled HDPE foams via rotational molding. J. Cell. Plast..

[43] Pfaendner R. (2022). Restabilization — 30 years of research for quality improvement of recycled plastics: Review. Polym. Degrad. Stab..

[44] Kartalis C.N., Papaspyrides C.D., Pfaendner R., Hoffmann K., Herbst H. (2000). Mechanical recycling of post-used HDPE crates using the restabilization technique. II: Influence of artificial weathering. J. Appl. Polym. Sci..

[45] Budisa N., Vlachopoulos J., Thompson M.R. (2025). Sustainability in rotational molding: A study on recycling and the influence of additives. Int. Polym. Process..

[46] Sorasan C., Ortega-Ojeda F.E., Rodríguez A., Rosal R. (2022). Modelling the Photodegradation of Marine Microplastics by Means of Infrared Spectrometry and Chemometric Techniques. Microplastics.

[47] Lowe P.S., Lais H., Paruchuri V., Gan T.-H. (2020). Application of Ultrasonic Guided Waves for Inspection of High Density Polyethylene Pipe Systems. Sensors.

[48] Gomes F.P.C. (2018). Nonlinear Ultrasonics for In-Line Quality Monitoring of Polymer Processing Methods. Ph.D. Thesis.

[49] Gomes F.P., West W.T.J., Thompson M.R. (2017). Effects of annealing and swelling to initial plastic deformation of polyethylene probed by nonlinear ultrasonic guided waves. Polymer.

[50] Cantrell J.H. (2004). Substructural organization, dislocation plasticity and harmonic generation in cyclically stressed wavy slip metals. Proc. R. Soc. Lond. A.

[51] Nagy P.B. (1998). Fatigue damage assessment by nonlinear ultrasonic materials characterization. Ultrasonics.

[52] Gomes F.P., Thompson M.R. (2017). Analysis of Mullins effect in polyethylene using ultrasonic guided waves. Polym. Test..

[53] Jhang K.-Y., Lissenden C.J., Solodov I., Ohara Y., Gusev V. (2020). Measurement of Nonlinear Ultrasonic Characteristics.

[54] Darmon M. (2024). Ultrasonic Modelling for Non-Destructive Testing.

[55] Liu C., Wang Q., Wu L., Song Y., Chen S., Liu C., Feng J. (2025). Nonlinear Ultrasonic Nondestructive Evaluation Method for Natural Aging Degree of Buried Medium Density Polyethylene Gas Pipeline During Long-Term Service. Polym. Eng. Sci..

[56] Gomes F.P.C., Thompson M.R. (2018). Nondestructive evaluation of sintering and degradation for rotational molded polyethylene. Polym. Degrad. Stab..

[57] Malyshev V.P. (1981). Veroyatnostno-Determinirovannoe Planirovanie Eksperimenta [Probabilistic-Deterministic Planning of Experiment].

[58] Zhu Y., Liu X., Tang Y., Xu K., Tang X., Zhu L., Xiong B. (2025). Recent Advances in the Synthesis of Commercially Available Phosphite Antioxidants. ChemistryOpen.

[59] Kartalis C.N., Papaspyrides C.D., Pfaendner R., Hoffmann K., Herbst H. (1999). Mechanical recycling of postused high-density polyethylene crates using the restabilization technique. I. Influence of reprocessing. J. Appl. Polym. Sci..

[60] Kube C.M., Arguelles A.P. (2017). Ultrasonic harmonic generation from materials with up to cubic nonlinearity. J. Acoust. Soc. Am..

[61] (2012). Plastics - Determination of Tensile Properties - Part 2: Test Conditions for Moulding and Extrusion Plastics.

[62] (2019). Plastics - Determination of Tensile Properties - Part 1: General Principles.

[63] Association of Rotational Molders International (ARM) (2003). Low Temperature Impact Test, Version 4.0.

[64] Almond J., Sugumaar P., Wenzel M.N., Hill G., Wallis C. (2020). Determination of the carbonyl index of polyethylene and polypropylene using specified area under band methodology with ATR-FTIR spectroscopy. e-Polymers.

[65] Rouillon C., Bussiere P.-O., Desnoux E., Collin S., Vial C., Therias S., Gardette J.-L. (2016). Is carbonyl index a quantitative probe to monitor polypropylene photodegradation?. Polym. Degrad. Stab..

[66] Zerbi G., Gallino G., Del Fanti N., Baini L. (1989). Structural depth profiling in polyethylene films by multiple internal reflection infra-red spectroscopy. Polymer.

[67] (2020). Standard Test Method for Compositional Analysis by Thermogravimetry.

[68] Q-Lab Corporation Technical Bulletin LU-8160: A Choice of Lamps for the QUV Accelerated Weathering Tester. https://www.q-lab.com/document-library/lu-8160-choice-lamps-quv-accelerated-weathering-tester.

[69] (2021). Standard Practice for Fluorescent Ultraviolet (UV) Lamp Apparatus Exposure of Plastics.

[70] (2019). Standard Practice for Fluorescent UV-Condensation Exposures of Paint and Related Coatings.

[71] (2022). Accelerated Exposure of Automotive Exterior Materials Using a Fluorescent UV and Condensation Apparatus.

[72] (2023). Standard Practice for Operating Fluorescent Ultraviolet (UV) Lamp Apparatus for Exposure of Materials.

[73] Augustine J.A., DeLuisi J.J., Long C.N. (2000). SURFRAD—A National Surface Radiation Budget Network for Atmospheric Research. Bull. Am. Meteor. Soc..

[74] (2024). Plastics—Methods of Exposure to Laboratory Light Sources—Part 3: Fluorescent UV Lamps.

[75] Spence A.G., Crawford R.J. (1996). The effect of processing variables on the formation and removal of bubbles in rotationally molded products. Polym. Eng. Sci..

[76] Gugumus F. (1999). Formation of Ester Functional Groups in Oxidizing Polymers. Polym. Degrad. Stab..

[77] Yunus S., Sefa-Ntiri B., Anderson B., Kumi F., Mensah-Amoah P., Sonko Sackey S. (2019). Quantitative Pore Characterization of Polyurethane Foam with Cost-Effective Imaging Tools and Image Analysis: A Proof-Of-Principle Study. Polymers.

[78] Dyuryagina A., Byzova Y., Ostrovnoy K., Demyanenko A., Tyukanko V., Lutsenko A. (2024). The Effect of the Microstructure and Viscosity of Modified Bitumen on the Strength of Asphalt Concrete. Polymers.

[79] Ahmad H., Rostami-Tapeh-Esmaeil E., Rodrigue D. (2024). The Effect of Chemical Crosslinking on the Properties of Rotomolded High-Density Polyethylene. J. Appl. Polym. Sci..

[80] Williams F.R., Jordan M.E., Dannenberg E.M. (1965). Effect of the chemical and physical properties of carbon black on its performance in polyethylene. J. Appl. Polym. Sci..

[81] Kovács E., Wolkober Z. (1976). The effect of the chemical and physical properties of carbon black on the thermal and photooxidation of polyethylene. J. Polym. Sci. Polym. Symp..

[82] Songwon Industrial, Sabo S.p.A. (2018). UV Stabilization of Polyethylene-Based Rotational Molding.

